# Electrolyte-Dependent, “Microscopically Irreversible” H‑Atom Transfer Kinetics of Ce-Based Metal–Organic Framework, Ce-MOF-808

**DOI:** 10.1021/acsami.5c21367

**Published:** 2026-01-05

**Authors:** Miguel A. Liuzzi-Vaamonde, Zaheer Masood, Bin Wang, Nikolay V. Tkachenko, Hyunho Noh

**Affiliations:** † Department of Chemistry and Biochemistry, 6187 The University of Oklahoma, Norman, Oklahoma 73019, United States; ‡ School of Sustainable Chemical, Biological and Materials Engineering, University of Oklahoma, Norman, Oklahoma 73019, United States

**Keywords:** proton-coupled electron transfer, H-atom transfer, Ce-based MOF, redox reaction, electrochemistry

## Abstract

Redox reactions at the interface of metal oxides and protic electrolytes almost always involve protons and electrons in equal amounts. Given the stoichiometry, these proton-coupled electron transfer (PCET) reactions are thermochemically equivalent to net H-atom transfer (HAT) reactions. The correlation between the chemical nature of solid catalysts and HAT kinetics has been employed for decades as the design principle for energy-relevant reactions (e.g., reactions of 2H^+^/H_2_). More recently, chemists have experimentally determined that a change in liquid electrolytes that alters the microenvironment at the redox-active sites has an equally profound impact on electrocatalysis involving PCET/HAT. Yet, precise correlations between the chemical nature of electrolytes and the PCET kinetics are, to date, rare in the literature. Herein, we report our findings using the Ce-based metal–organic framework, Ce-MOF-808, as a model system. Each Ce_6_(μ_3_–O)_4_(μ_3_–OH)_4_(OH)_6_(H_2_O)_6_ node of this MOF undergoes a 1H^+^/1e^–^ redox reaction. Using chronoamperometry and the Cottrell analysis, we have determined that the PCET hopping kinetics within the pores of Ce-MOF-808 can change by orders of magnitude by altering the buffer species and the proton activity of the electrolyte. Furthermore, in all buffers, reductive reactions were ∼3–10 times faster in kinetics than the reverse oxidative reaction with the same electrochemical driving force, suggesting that the system, at first glance, violates the principle of microscopic reversibility. Isothermal titration calorimetry (ITC) and computational simulations corroborated that the buffer-node binding thermodynamics are quite distinct, depending on the chemical nature of the buffer and the oxidation state of the node. Together, these results suggest that the substrate and the product during the oxidative vs reductive reaction of Ce-MOF-808 are chemically different species, which explains the apparent ‘microscopic irreversibility.’ Thus, the rational modulation of electrolytes can dramatically enhance PCET kinetics, even though the solid electrodes remain identical. Implications of these findings are contrasted with the electrochemical/electrocatalytic behavior of other redox-active MOFs, heterogeneous catalysts, and enzymatic systems at the solid–liquid interface.

## Graphical abstract

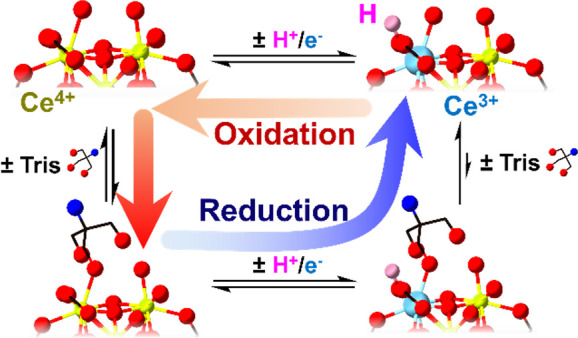

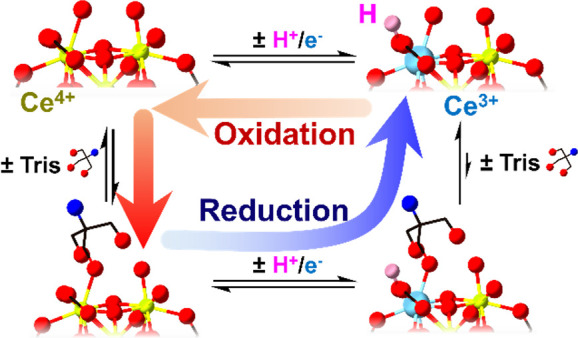

## Introduction

Hydrogen-atom transfer (HAT) reactions at solid–gas and solid–liquid interfaces lie at the center of reactions relevant to renewable energy and industrial-scale synthesis of commodity chemicals.^1–4^ Electrochemically, surface-bound H-atoms are often a product of proton-coupled electron transfer (PCET) reactions with equimolar amounts of protons (H^+^’s) and electrons (e^–^’s); this reaction is called the Volmer reaction (eq 1).^2,5,6^ The yielded surface–H bonds essentially serve as a ‘reservoir’ of chemical energy to drive small molecule activations, including but not limited to H_2_ evolution reaction (HER), CO_2_ reduction to chemical fuels, and N_2_ reduction to ammonia (eqs 2–4).^2,3,5,7^

$$\mathrm{Surface}+H^{+}+e^{-}⇄\mathrm{Surface}-H$$ 1

$$2H^{+}+2e^{-}⇄H_{2}$$ 2

$${\mathrm{CO}}_{2}+2H^{+}+2e^{-}⇄\mathrm{CO}+H_{2}O$$ 3

$$N_{2}+6H^{+}+6e^{-}⇄2{\mathrm{NH}}_{3}$$ 4

The design of optimal catalysts for small molecule activations, like those shown in eqs 2–4, commonly stem from an understanding of the Sabatier Principle.^8^ In HER, for example, highly active catalysts must present H-atom binding energy (Δ*G*°_H_) that is nearly identical to the free energy necessary to homolytically cleave the H–H bond in H_2_. Thus, plotting Δ*G*°_H_ of various catalysts against their HER activities result in a volcano-shaped plot, where optimal catalysts like Pt exhibit Δ*G*°_H_ neither too strong nor too weak.^9^

While undoubtedly, the Sabatier Principle and the resulting volcano plot have propelled catalyst discovery for decades, there still remains a challenge to apply these concepts to HAT reactions at *chemically complex interfaces*.^10,11^ The Δ*G*°_H_ values are most commonly computed on solid-vacuum interfaces using a single-crystalline, defect-free model. Experimentally, however, these catalysts form surface–H bonds at solid–liquid interfaces, and thus can undergo dynamic interactions with solvents, buffers, ions, and many other components within the electrolyte.^12–15^ Even for a single-crystalline Pt(111) surface, for example, there are at least two distinct H-atom binding sites that can present distinct reactivity, particularly in the presence of coordinating anions like phosphate.^16,17^ Yet, in a typical volcano plot, both the Δ*G*°_H_ value and the catalytic rate of a given catalyst are often implicitly ascribed to a catalytic motif from crystallographic data, with no liquid in contact.^2,8–10^

The above complications are further amplified when catalytic motifs exist within porous materials like metal–organic frameworks (MOFs), the focus of this article. MOFs have been extensively employed as catalysts or their support.^18,19^ In these cases, the pores are as critical as the catalyst itself; within the pores, solvents and other liquid components can be arranged in a fashion distinct from the bulk solution, inducing changes to its physical and chemical properties.^20–22^

To enhance the quantitative accuracy of the Sabatier Principle, here, we report our findings on the PCET reaction of a Ce-based MOF, Ce-MOF-808 (Figure 1).^23^ The hexanuclear, Ce_6_(μ_3_–O)_4_(μ_3_–OH)_4_(OH)_6_(H_2_O)_6_ nodes of this MOF have been shown to be redox active;^24^ furthermore, we have recently demonstrated that this redox reaction involves a Ce^4+/3+^ redox couple and (de)­protonation of either the bridging μ_3_–O­(H) or the terminal −OH_(2)_ group with a 1:1 proton-to-electron stoichometry.^25^ Because this is a net HAT reaction, we referred to the free energy of this reaction as the Ce^3+^O–H bond dissociation free energy (BDFE).^7^ The average BDFE was experimentally measured to be 78(2) kcal mol^–1^ ^25^; see eqs 5–9 in Scheme 1.

**Scheme 1:**
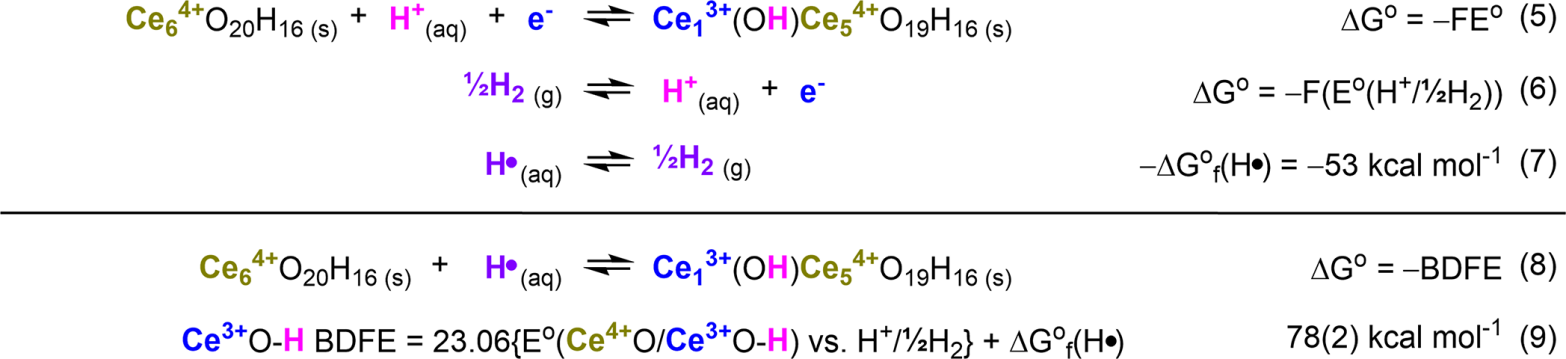
Derivation of Ce^3+^O–H BDFE from Electrochemical Standard Potential (*E*°) of the Ce^4+^O/Ce^3+^OH Redox Reaction

**Figure 1:**
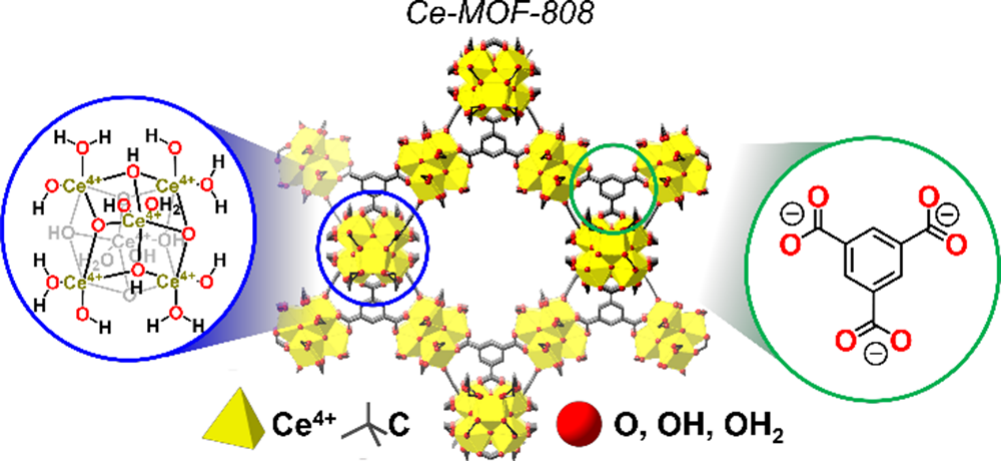
Structures of Ce-MOF-808 and its hexanuclear, Ce-oxo node, and organic linker.

We build on these findings to demonstrate that the kinetics of transferring 1H^+^/1e^–^ within Ce-MOF-808 are highly dependent on the exact chemical nature of the electrolyte, including buffer identity and proton activity. As described below, the difference in PCET kinetics is intimately correlated to the interaction of buffer molecules with the Ce_6_ node of Ce-MOF-808. These findings quantitatively demonstrate that electrolytes have equally important contributions in defining the correlation between thermodynamics and kinetics of PCET reaction. Implications of these results that directly challenge implicit assumptions within the Sabatier principle are discussed.

## Experimental Section

### Synthesis and Characterization of Ce-MOF-808

Nanocrystalline Ce-MOF-808 was synthesized, and the solvent was removed from the MOF pores using a previously reported procedure.^24^ The MOF was stored under an N_2_ atmosphere at room temperature until further use. Physical characterization of this MOF, including the N_2_-adsorption–desorption isotherm, powder X-ray diffraction (PXRD) pattern, and scanning electron microscopy (SEM) image, can be found in the Supporting Information (SI; Figures S1–S3).

### Brief Elaboration on the Electrochemical Measurements Using Ce-MOF-808

The Ce-MOF-808-based electrode was prepared through simple drop-casting onto a fluorine-doped tin oxide (FTO) surface, following our previous report.^25^ Here onward, this electrode will be referred to as Ce-MOF-808|FTO.

All electrochemical measurements were performed in aqueous electrolytes with buffers such as 3-(N-morpholino)­propanesulfonic acid (MOPS), tris­(hydroxymethyl)­aminomethane (Tris), and boric acid (H_3_BO_3_), and 0.1 M of NaCl. The concentration of the buffers used, denoted as [buffer], ranged from 0.1 to 1.0 M (vide infra for more details). The pH values of these electrolytes were adjusted between 7 and 10, depending on the buffer identity. For all measurements, Pt wire and Ag/AgCl (3 M KCl) were used as counter and reference electrodes, respectively. Unless otherwise noted, cyclic voltammograms (CVs) were measured at a scan rate (υ) of 25 mV s^–1^. Further details can be found in the SI. Using the same experimental setup as that used for CVs, controlled potential electrolyses (CPEs) of the same films were used to further understand the kinetics of redox reactions within Ce-MOF-808.

## Results

### Surface Charges of Ce-MOF-808

Surface charges of Ce-MOF-808 crystals were probed using zeta-potentials (*E*(ζ)) in buffers and pHs used in the electrochemical measurements (vide infra). *E*(ζ) of Ce-MOF-808 in buffers like MOPS and H_3_BO_3_ (0.1 M in concentration) that are anionic upon deprotonation, were generally negative. In Tris buffer, at pH 7 and 8, *E*(ζ) of the MOF was positive, but became negative at pH 9, likely due to deprotonation of otherwise cationic [TrisH]^+^. For all buffers and pHs,

We note that the low colloidal stability of Ce-MOF-808 proved difficult to measure *E*(ζ), and thus, we prefer not to interpret these values excessively.

### Brief Elaboration on the CVs of Ce-MOF-808|FTO with Varying Scan Rates

This section describes how the anodic and cathodic peak current densities (*j*_p,a_ and *j*_p,c_) scale with the υ in CVs of Ce-MOF-808|FTO. We have previously conducted these measurements in a pH 8-adjusted 0.1 M Tris buffer. Here, we have expanded on our previous work to include varying buffer species and concentrations with pH values adjusted between 7 and 10 (vide supra). As noted in the next section, these electrolytes were further employed to determine PCET kinetics within Ce-MOF-808.

The plots showing log of *j*_p,a_ or *j*_p,c_ values vs log­(υ) are shown in Figures S7–S11. Regardless of the buffer identity, its concentration, or the proton activity, the two parameters linearly scaled to each other with slopes ranging between $\frac{1}{2}$ to 1; slopes are listed in Table S4. In Tris and borate-based electrolytes, regardless of the pH, the slopes were always $>\frac{1}{2}$ for both anodic and cathodic processes, suggesting that the reaction rate is controlled by the kinetics of H^+^/e^–^ addition or removal at the Ce_6_ node. In contrast, in MOPS-containing electrolytes, the slopes were closer to $\frac{1}{2}$ , suggesting that at least in a CV time scale, the reaction is diffusion-controlled. At a glance, this is intuitive as MOPS is more sterically demanding than Tris or borates. Yet, an increase in MOPS concentration resulted in a slope approaching 1, suggesting that the reaction becomes more kinetically controlled; this trend holds for Tris and borate-based electrolytes as well.

### CPE Using Ce-MOF-808|FTO and Apparent Diffusion Coefficients

We begin this section by describing CPE measurements of Ce-MOF-808|FTO at pH 8-adjusted 0.1 M Tris buffer as a case study. CVs of Ce-MOF-808|FTO in this electrolyte exhibited one reversible Faradaic couple, which can be attributed to the Ce^4+^O/Ce^3+^OH redox reaction. Here, we emphasize that this H^+^/e^–^ stoichiometry has been established in all buffers employed here by us previously, and we direct the readers to the following reference.^25^ CPEs were conducted at two applied potentials, which are either cathodic or anodic of the peak potentials (*E*_p,c_ and *E*_p,a_) by 240 mV; these potentials are denoted *E*_CPE,c_ or *E*_CPE,a_, respectively, in the inset of Figure 2A. This ensures that all electrochemically active Ce cations are in either the reduced or the oxidized state.

**Figure 2:**
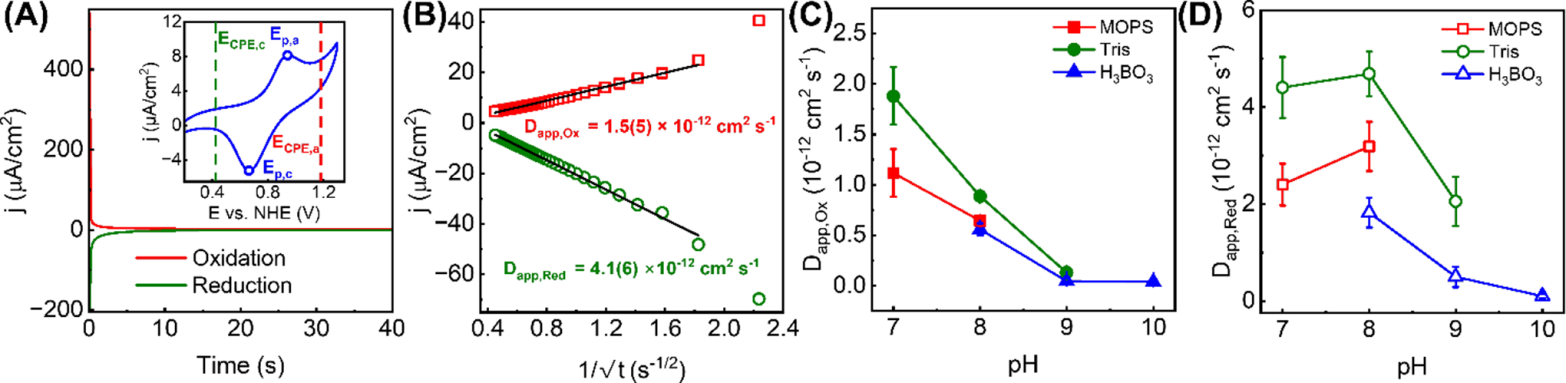
(A) Representative chronoamperometric curves of Ce-MOF-808|FTO measured at *E*_CPE,a_ and *E*_CPE,c_. The inset shows representative quasi-reversible CVs with positions of *E*_CPE,a_ and *E*_CPE,c_ with respect to the peak potentials, *E*_p,a_ and *E*_p,c_. (B) Representative Cottrell plots derived from the chronoamperometric curves shown in (A) with *D*_app,Ox_ and *D*_app,Red_ derived from the slopes. The errors in the *D*_app_ values represent 1σ of linear regressions. For (A,B), 0.1 M Tris buffer at pH = 8 was used. Plots of (C) *D*_app,Ox_ or (D) *D*_app,Red_ vs pH of Ce-MOF-808|FTO using various buffers as electrolytes. All buffer concentrations are set at 0.1 M; values at other buffer concentrations can be found in the SI. Errors in these two plots represent 1σ of triplicate measurements. An increase in buffer concentration caused *E*(ζ) to approach 0 mV, which suggests that buffer species are indeed ‘screening’ the MOF surface charge. These results can be found in Figure S4 and Table S1 in the SI.

Upon conducting CPEs at these potentials, we observed a rapid decrease in measured current density (*j*) as a function of time; at *t* = 5 s and beyond, the measured *j* was <5% of that at *t* = 1 s (Figure 2A). Using the Cottrell equation (eq 10), these chronoamperometric curves yield the apparent diffusion coefficients in oxidative or reductive reactions, denoted *D*_app,Ox_ or *D*_app,Red_, respectively.^27–32^ In eq 10, *C*_Ce_ refers to the ‘effective concentration’ of Ce cations within the MOF film, which was estimated from the dimensions of the film and the total amount of charge passed during each CPE measurement; see the following references and the SI for details.^27,32^ In this equation, *F* denotes the Faraday constant (96485 C mol^–1^).

$$j=\frac{{\mathrm{FC}}_{\mathrm{Ce}}\sqrt{D_{\mathrm{app}}}}{\sqrt{\pi t}}$$ 10

During CPE, the node-bound H^+^/e^–^ ‘hop’ to the adjacent node, and thus the PCET hopping kinetics throughout the MOF lattice can be mimicked using diffusion coefficients; as discussed later, these diffusion coefficients can be directly translated to apparent rate constants (*k*_app_) using the distance at which the bound H^+^/e^–^ would have to hop.

As shown in Figure 2B and Table 1, *D*_app,Red_ is ca. 3–10 times higher than *D*_app,Ox_; in other words, with the same amount of thermodynamic driving force, the addition of H^+^/e^–^ to the Ce_6_ node is kinetically more favorable than the reverse oxidation reaction. PXRD patterns of Ce-MOF-808|FTO before and after CPE measurements were identical, suggesting the retention of crystallinity (see Figure S2).

**Table 1:** *D*_app_ Values of Ce-MOF-808|FTO Determined from Cottrell Equations in Electrolytes with Distinct pHs and Buffers^a^

	*D*_app.Ox_ (10^–12^ cm^2^ s^–1^)			*D*_app.Red_ (10^–12^ cm^2^ s^–1^)		
pH	MOPS	Tris	H_3_BO_3_	MOPS	Tris	H_3_BO_3_
7	1.1(2)	1.5(2)		2.4(4)	3.6(4)	
8	0.64(5)	0.89(4)	0.56(6)	3.2(5)	4.7(5)	1.8(3)
9		0.13(1)	0.05(3)		2.1(5)	0.5(2)
10			0.04(2)			0.10(4)

a The errors on this table represent 1σ of at least triplicate measurements.^26^ All buffer concentrations are at 0.1 M. *D*_app_ values with various concentrations of buffers can be found in the SI.

The concentration of the Tris buffer was altered while retaining the pH at 8 to examine its impact on *D*_app_ values. As shown in Figure S15 and Table S5, *D*_app,Ox_ was nearly constant when the Tris concentration varied between 0.1 and 1 M, ranging between 0.8(1)–1.1(3) × 10^–12^ cm^2^ s^–1^. *D*_app,Red_ had an apparent decrease as the concentration of Tris increased; these results are further discussed later.

### Diffusion Coefficients and PCET Hopping Kinetics of Ce-MOF-808|FTO at Various pHs and Buffers

Using the identical experimental protocol as described above, we conducted a series of CPE measurements in electrolytes using buffers beyond Tris, and at various pHs. As shown in Figure 2C and Table 1, *D*_app,Ox_ seemingly decreased asymptotically as pH increases, and this trend was largely consistent over all three buffers, MOPS, H_3_BO_3_, and Tris. In contrast, *D*_app_,_Red_ was *highly dependent on the electrolyte pH and buffer identity* (Figure 2D and Table 1). In general, *D*_app_,_Red_ measured in Tris buffer were consistently the largest at any given pH, followed by those measured in MOPS, then in H_3_BO_3_ (see the SI). Notably, H_3_BO_3_ or its deprotonated form, [H_2_BO_3_]^−^ is sterically the least demanding, and yet exhibited lower *D*_app,Red_ than more sterically demanding MOPS or Tris. Thus, we believe the measured differences in *D*_app_ values between different buffers are not simply due to diffusive complications. Instead, buffers with different functional groups must be interacting in a distinct fashion with the Ce_6_ node of Ce-MOF-808 such that, at least for the reduction of Ce^4+^O to Ce^3+^OH, its hopping kinetics are highly sensitive to the exact chemical composition of the electrolyte.

Figure 2D further highlights an intriguing trend in *D*_app,Red_ of Ce-MOF-808, where its reduction kinetics are seemingly the highest at pH 8, regardless of the buffers. We will return to this trend in the Discussion section.

We have further measured *D*_app_ values at various concentrations of Tris buffers when the pH values of the electrolytes were adjusted to 7–9. Representative Cottrell plots are shown in Figures S14 and S16. As noted above, *D*_app_ values exhibited complex dependence that was unique to the pH and the buffer identity. For example, in a pH 7-adjusted Tris buffer, the *D*_app,Red_ seemingly increased from 3.6(4) to 8(1) when the Tris concentration increased from 0.1 to 0.4 M. However, at higher concentrations, *D*_app,Red_ decreased (see Table S5 and Figure S21). *D*_app,Ox_ stayed nearly identical to those observed in pH 8-adjusted Tris buffer (vide supra). In contrast, in pH 8-adjusted H_3_BO_3_ buffer, the *D*_app,Ox_ decreased with the increase in buffer concentration, while *D*_app,Red_ remained nearly identical (Table S5 and Figure S22).

### Diffusion Coefficient vs Amount of Available Ce^4+/3+^ at Electrode Surface

Up to this point, all *D*_app_ values have been measured by applying *E*_CPE,a_, then *E*_CPE,c_, following the direction of CVs. To examine if this caused any effect on *D*_app_ values, we conducted similar CPE measurements, but starting with the reductive reaction. Here, we employed pH 8-adjusted 0.1 M MOPS buffer as the measured *D*_app,Ox_ and *D*_app,Red_ under this condition is quite distinct from each other.

As shown in Figure S23, *D*_app,Red_ did not change significantly when the cathodic potential (*E*_CPE,c_) was first applied. This suggests that the apparent anisotropy in *D*_app_ values is *intrinsic* to the redox properties of Ce-MOF-808.

### Conversion of *D*_app_ to Rate Constants of PCET Hopping

*D*_app_ values have been employed to decipher PCET hopping kinetics, as similar values have been reported for other redox-active MOFs.^27–32^ In this section, we describe the conversion of *D*_app_ values to the apparent hopping rate constants (*k*_app_) by using eq 11 and by knowing the distance between the two redox active sites (*r*).^28^ Here, we note that this apparent rate constant is related to the microscopic hopping of protons and electrons, rather than the *intrinsic* rate constant of adding protons and electrons to the pristine node.^28,33^ This is why these rate constants have the units of s^–1^, while those typically reported for PCET reactions of homogeneous species are in the units of M^–1^ s^–1^.^34–36^ Further discussions on this distinction can be found in the Discussion section.

Derivation of *k*_app_ values allows comparisons of PCET hopping kinetics beyond MOF-based systems (as described in the Discussion section). In essence, *k*_app_ is the apparent ‘diffusion’ rate constant of H^+^/e^–^ bound on the node to hop to an adjacent site. Furthermore, *k*_app_ values can be directly applied to many theories defining rate vs driving force relationships (see the Discussion section for more details).

$$k_{\mathrm{app}}=\frac{D_{\mathrm{app}}}{r^{2}}$$ 11

Because CPE measurements do not indicate *r*, we turned to the crystallographic results of Ce-MOF-808 reported previously (cf.^23^). The derived *k*_app_ values are shown in Table 2. Here, we considered two Ce–Ce distances. The *intranode* Ce–Ce distance of ca. 3.5 Å is the shortest, and therefore, according to the Marcus theory, this charge hopping should be the most favorable. As described previously, Ce_6_ nodes within only a few crystallographic units away from the underlying FTO surface are electro-active.^25^ Thus, the intranode hopping should quickly become kinetically uphill. We also considered the *internode* distance of 7.7 Å. This internodal distance is believed to be the most relevant for through-film charge transport, as discussed later. No other internode distances were considered as PCET hopping of *r* > 10 Å is unlikely.

**Table 2:** *k*_app_ Values of Ce-MOF-808|FTO Determined from *D*_app_ Values and Two Ce–Ce Distances Derived from Crystallographic Data^a^

	*k*_app_ (*r* = 3.5 Å)/*k*_app_ (*r* = 7.7 Å) (10^3^ s^–1^)					
	oxidative			reductive		
pH	MOPS	Tris	H_3_BO_3_	MOPS	Tris	H_3_BO_3_
7	0.9(2)/0.19(3)	1.2(2)/0.25(3)		2.0(3)/0.40(7)	2.9(3)/0.61(7)	
8	0.5(4)/0.09(1)	0.73(3)/0.15(1)	0.46(5)/0.09(1)	2.6(4)/0.54(8)	3.8(4)/0.79(8)	1.5(2)/0.30(5)
9		0.11(1)/0.02(1)	0.04(2)/0.008(5)		1.7(4)/0.35(8)	0.4(2)/0.08(3)
10			0.03(2)/0.007(3)			0.08(3)/0.017(7)

a All standard errors in the reported table are propagated from those of *D*_app_ values reported in Table 1.^26^

Attempts to measure intrinsic electron transfer rate constants (*k*_ET_) were unsuccessful. Typically, *k*_ET_ values are determined through examining how the difference between *E*_p,c_ and *E*_p,a_ (Δ*E*_p_) changes as a function of a CV scan rate (υ); this method is typically called the Nicholson method.^37,38^ As shown in Figure S24, this method proved challenging as the Δ*E*_p_-derived dimensionless parameter, Ψ, did not scale with υ in an expected manner. Thus, we have further measured the charge transfer resistance (*R*_CT_) of Ce-MOF-808|FTO by using electrochemical impedance spectroscopy (EIS).^39^ While this measurement was successful, sample-to-sample variation was high. Namely, the R^_CT_^-derived *k*_ET_ values of four nominally identical Ce-MOF-808|FTO ranged between 5.7(5) × 10^–7^ and 1.07(4) × 10^–5^ cm s^–1^. This inconsistency is likely due to the difference in the amount of electroactive Ce near the underlying FTO, as further indicated by the difference in the EIS-derived double-layer capacitance (Figure S25 and Table S7). Such a large sample-to-sample variation was not observed in *D*_app_ measurements because *D*_app_ probes the kinetics of H^+^’s/e^–^’s hopping within the MOF lattice after the initial charge transfer. Thus, we prefer to focus on *D*_app_-derived *k*_app_ values in this study.

### Half-Wave Potentials of Ce^4+^O/Ce^3+^OH Redox under CPE Reaction Conditions

The focus of this work is on PCET kinetics rather than thermodynamics, as we have established the latter in our previous publication.^25^ Nevertheless, we briefly note here that the half-wave potentials (*E*_1/2_) derived from the CVs were somewhat dependent on the buffer concentrations, though not to the extent of *D*_*app*_ values. In general, *E*_1/2_ was higher by ≤100 mV when the buffer concentration increased from 0.1 to 0.2 M and remained nearly identical with further increases in buffer concentration. The only exception to this trend is H_3_BO_3,_ and we describe why the borate-containing buffer was an exception later.

This noticeable change in *E*_1/2_ by ≤100 mV equals ±2 kcal mol^–1^ error on the thermodynamics of this PCET reaction. This error range is within that reported previously for Ce-MOF-808 (see Scheme 1), and is typical for similar values reported for MOFs, metal oxides, and even molecular species.^7,40,41^

### Thermodynamics of Buffer Interactions with Ce-MOF-808

To better understand the interaction between Ce-MOF-808 and buffers, we conducted isothermal titration calorimetry (ITC) measurements using a modified procedure from that reported previously by Drout et al.^42^ Experimental details can be found in the SI. ITC measurements were conducted at pH 8-adjusted aqueous solutions for MOPS and Tris buffers, and at pH 9 for H_3_BO_3_ to ensure the partial deprotonation of buffer molecules. The concentrations of the buffers ranged between 8 and 50 mM and were individually determined through a series of optimizations. As shown in Figure 3 and Table 3, all buffer-MOF interactions were thermoneutral to exergonic. The reaction between borate and Ce-MOF-808 was the most exergonic, with the Δ*G* of ca. −6.3 kcal mol^–1^. Surprisingly, this borate-Ce-MOF-808 interaction was largely driven entropically and was, if at all, endothermic. This contrasts with MOPS and Tris interactions with Ce-MOF-808, where for Δ*H* was below zero. Tris–MOF interaction was largely enthalpically driven, where TΔ*S* is negative (i.e., energetically unfavorable).

**Figure 3:**
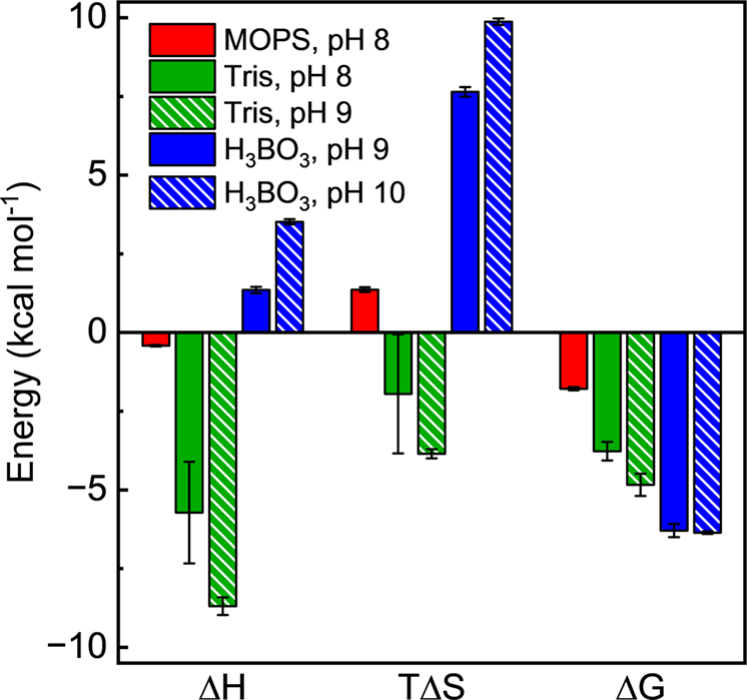
Figure summarizing thermodynamic parameters, Δ*H*, *T*Δ*S*, and Δ*G* of interactions between buffers and Ce-MOF-808. All errors are 1σ of triplicate measurements.

**Table 3:** Summary of Thermodynamic Parameters, Δ*H*, *T*Δ*S*, and Δ*G* Derived from the ITC Measurements of Ce-MOF-808 in Various Buffers and pH^a^

buffer	pH	Δ*H* (kcal mol^–1^)	*T*Δ*S* (kcal mol^–1^)	Δ*G* (kcal mol^–1^)
MOPS	8	–0.4(1)	1.4(1)	–1.8(1)
Tris	8	–6(2)	–2(2)	–3.8(3)
Tris	9	–8.7(3)	–3.8(1)	–4.8(4)
H_3_BO_3_	9	1.4(1)	7.6(2)	–6.3(2)
H_3_BO_3_	10	3.5(1)	9.9(1)	–6.4(1)

a All errors in this table represent 1σ of triplicate measurements.

At pH 8, about half of the Tris molecules within the solution remain protonated as [TrisH]^+^. This holds true for pH 9-adjusted H_3_BO_3_. To examine the role of protonation, we conducted similar ITC experiments in pH 9-adjusted Tris and pH 10-adjusted borate solutions. As shown in Figure 3, deprotonation of [TrisH]^+^ to Tris in pH 9 resulted in a slight increase in ΔG largely due to an increase in ΔH. In contrast, the thermodynamic parameters of borates in pH 9 vs 10 were more similar than different.

### Computational Modeling of Interactions between Ce_6_ Node and Buffers

To computationally model the buffer-MOF interactions, we performed free energy calculations of “buffer insertion” reactions (Δ*G*_Buf_) to the cluster model of the Ce_6_ node of Ce-MOF-808. The ωB97M-V/def2-TZVPPD//ωB97X-D4/def2-SVPD level of theory. We have employed both the SMD­(water) implicit solvent model and one explicit water molecule as solvent.^43–46^ Our primary focus was the insertion of buffers onto the node via the displacement of one or more of −OH/–OH_2_ groups on the node. However, as described below, our initial attempts at examining the buffer-node interaction also resulted in buffer-dependent proton transfer. Full computational details are provided in the SI.

We began with computational modeling of the interaction between a singly deprotonated [H_2_BO_3_]^−^ and the pristine Ce_6_ node. Intriguingly, we observed a proton transfer from μ_3_–OH of the node to the buffer anion. The p*K*_*a*_ values of these μ_3_–OH moieties were calculated to be 7–9, even in the presence of a H_2_O molecule, which indeed suggests that this proton transfer is thermodynamically favorable (see Tables S8 and S9 for the exact p*K*_a_ values). Given the p*K*_a_, MOPS and Tris did not undergo a similar proton transfer. We believe this explains why (A) *E*_1/2_ vs NHE measured in borate buffers resulted in a relatively large shift when buffer concentrations were altered, and (B) borate-node interaction is entropically driven.

Next, we performed calculations solely focusing on insertion reactions. In this section, we will largely focus on thermodynamically most stable states for each buffer, with their energies and structures shown in Table 4 and Figure 4, respectively; see the SI for the full list of ΔG_Buf_ in various binding motifs (Table S9). Figure 4A–C summarize the most thermodynamically stable, optimized structures of the three buffers bound to the pristine Ce_6_ node. For H_3_BO_3_/[H_2_BO_3_]^−^, the reaction free energies to displace a terminal −OH_2_ (Δ*G*_Buf_) ranged from −0.3 to +2.0 kcal mol^–1^. In contrast, the Δ*G*_Buf_ values to insert borate buffer to terminal −OH sites or binding in μ_2_-fashion were much more endergonic (+3.5 to +9.5 kcal mol^–1^). For Tris, our simulations indicate that displacement of terminal −OH (via deprotonation of Tris to release H_2_O) is more favorable, but still less energetically preferable than the displacement of terminal −OH_2_, with the difference in free energy of the two states ΔΔ*G*_Buf_ of 2–3 kcal mol^–1^. Similar reactions with MOPS were, overall, mildly to highly endergonic (+0.7 to +20.9 kcal mol^–1^); see Table 4 for all Δ*G*_Buf_ values.

**Figure 4:**
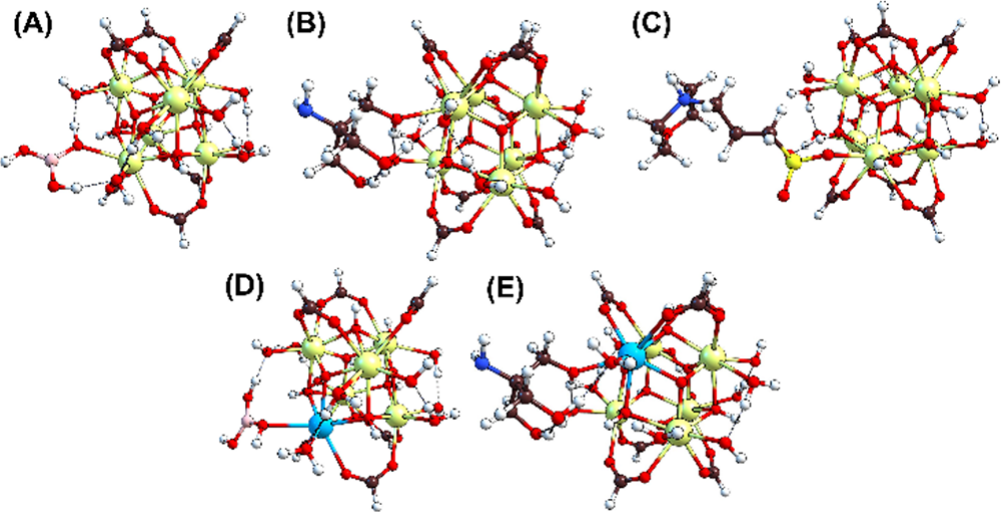
Optimized structures of the Ce_6_ node with inserted buffers. (A) H_3_BO_3_ bound via terminal −OH_2_ substitution; (B) Tris bound via terminal −OH_2_ substitution; (C) MOPS bound via terminal −OH_2_ substitution. Panels (D, E) show corresponding reduced node structures with the Ce^3+^ site (cyan) and protonation of a terminal −OH site. Atom colors: Ce^4+^ = light yellow, Ce^3+^ = cyan, O = red, C = gray, H = white, *N* = blue, B = pink, S = yellow.

**Table 4:** Summary of Δ*G*_Buf_ between Various Buffers and Ce_6_ Nodes with or without an Extra 1H^+^/1e^–^

table entry^a^	buffer	number of added H^+^/e^–^	Δ*G*_Buf_ (kcal mol^–1^)
A	H_3_BO_3_	0	–0.29
B	Tris	0	–2.11
C	MOPS	0	0.74
D	H_3_BO_3_	1	3.21
E	Tris	1	1.97

a Table entry corresponds with the labels on Figure 4.

We further performed similar simulations as above using Tris and H_3_BO_3_, but with a singly reduced node with a hydrogen atom added to the adjacent terminal −OH group. This effectively reduced the closest Ce cation to the 3+ oxidation state. For both of these species, Δ*G*_Buf_ became more endergonic by ∼3–4 kcal mol^–1^, suggesting that buffers are less likely to be coordinated upon reduction; see Figure 4D,E for the optimized structures. As described in the Discussion section, this difference in buffer-node binding thermodynamics drives the apparent anisotropy in PCET hopping kinetics of Ce-MOF-808.

## Discussion

### CPE-Derived Diffusion Coefficients of Ce-MOF-808|FTO and in Related MOF-Based Systems

CPE was used to derive apparent diffusion coefficients (*D*_app_’s) of the redox reaction within the pores of Ce-MOF-808. The *D*_app_ values were measured for both the oxidative and reductive reactions involving 1H^+^/1e^–^ transfer to and from the Ce_6_ node and thus involve the Ce^4+^O/Ce^3+^OH couple.^25^ The *D*_app_ values obtained from Cottrell analysis of chronoamperometric data represent the effective proton/electron hopping between redox-active cerium within the MOF lattice. Unlike conventional diffusion coefficients that describe the movement of solvated ions through porous materials, these values quantify the rate of charge propagation via PCET across the MOF. This interpretation reflects the lack of an ion concentration parameter in the Cottrell equation (eq 10). The measured *D*_app_ values were roughly within the expected range for an MOF film fabricated via drop-casting; based on the following references, MOF-based films synthesized in a similar fashion exhibit *D*_app_ values ranging from 10^–11^ to 10^–13^ cm^2^ s^–1^.^27,28,47^

We will first focus on *D*_app_ values as these values are widely used to understand the redox kinetics and charge hopping within MOFs (vide infra). Discussions of *k*_app_ values are described later.

Anisotropic *D*_app_ values have previously been reported by others. A Zr-based MOF, NU-1000, for example, features redox-active pyrene-based organic linkers with *D*_app_ values that depend on the orientation of the MOF crystals with respect to the underlying electrode, which in turn influences ion diffusion within the MOF pores.^28^ The same MOF decorated with metallocene or 2,2′-bipyridine complexes has been used by Morris and co-workers to determine the *D*_app_ values of electrons and the charge-balancing ions (Scheme 2A).^30,31^ In all cases, electrons reside within the redox-active units and must hop to the nearby unit during CPE measurements; thus, these reactions are considered as outer-sphere electron transfer (ET) reactions.

**Scheme 2:**
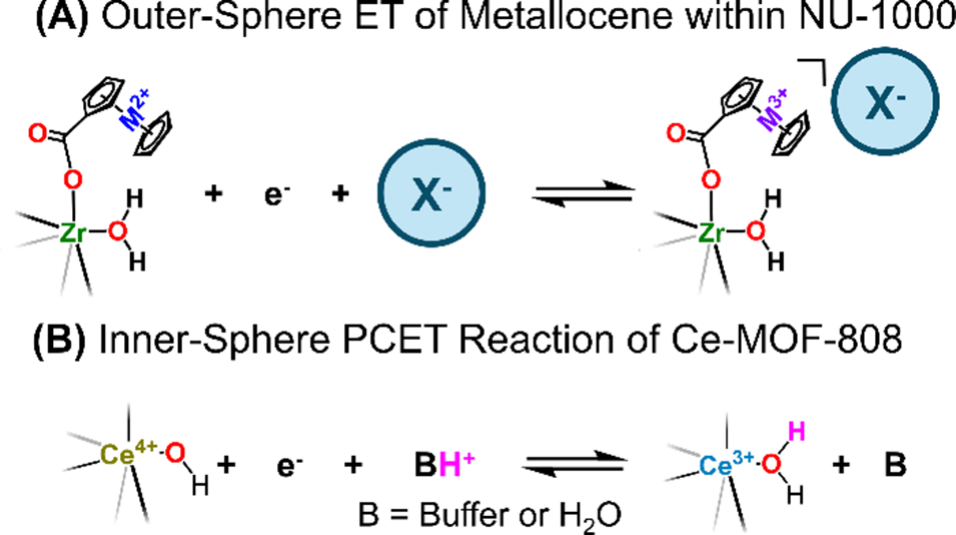
Schematic Illustration Showing (A) Ion Coupling in Outer-Sphere ET of Metallocene within NU-1000 and (B) O–H Bond Formation Due to Inner-Sphere PCET Reaction within Ce-MOF-808

a In (B), only one of the four proton topologies that are possible on the reduced Ce-MOF-808 node is considered (see ref 51). This is further elaborated later.

In contrast, the PCET reaction of Ce-MOF-808 yields a covalent O–H bond at the Ce_6_ node (Scheme 2B).^25^ Typically, O–H bonds on a surface of metal-oxo sites are localized, unlike the electrons that are ‘loosely coupled’ to counterions.^7,41^ Our computational simulations also indicate highly localized spin density on one of the six Ce cations upon reduction, suggesting electron localization. We and others have reported similar computational observations upon adding 1H^+^/1e^–^ to the Ti-oxo nodes of the MOF, Ti-MIL-125.^48–50^ In other words, Ce-MOF-808 undergoes an *inner-sphere PCET reaction.*

For Ce-MOF-808 and other cerium-oxo-based systems, reduction of Ce^4+^O to Ce^3+^OH typically causes large lattice strain as the volume of the Ce^3+^ cation is ca. 50% larger than that of Ce^4+^.^52–54^ Thus, it is not surprising that the delocalization of electrons within CeO_x_ is unfavorable due to ion expansion and contraction within the lattice.

As described below, the *inner-sphere* nature of the PCET reactions on Ce-MOF-808 is fundamental to understanding the apparent redox anisotropy and kinetic vs electrolyte identity relationship.

### Buffer-Dependent *D*_app_ Values of Ce-MOF-808 and Their Correlation to the Buffer-Node Interaction

A series of CPE measurements using Ce-MOF-808|FTO have further demonstrated that *D*_app_ values depend on the identity and concentration of aqueous buffers.

The difference in the free energy of chemical interactions between buffers and Ce_6_ nodes of Ce-MOF-808 may, at first glance, explain the difference in *D*_app_ values observed in our work. Borate anions bind to the surfaces of metal oxides like nickel and cobalt oxides,^12,14^ and indeed, our ITC measurements indicate exergonic borate-node interactions. Using instead a weakly coordinating MOPS, on the other hand, the Δ*G* was nearly thermoneutral; we will elaborate on the exact reaction and the ITC-derived ΔH/ΔS later.

Electrolyte-dependent redox kinetics within MOFs have commonly been explained using the mobility of charge-compensating ions within the MOF pores. For example, *D*_app_ values of metallocene within NU-1000 (vide supra) are larger when tetrakis­(pentafluorophenyl)­borate ([BAr^F^_5_]^−^) ions are introduced as charge-compensating anions as compared to hexafluorophosphate (PF_6_^–^).^31^ The weak Coulombic interaction between the oxidized, and therefore cationic, metallocenium and large [BAr^F^_5_]^−^ facilitates ion movement within the MOF pores compared to the same reaction with a smaller PF_6_^–^ anion. Thus, in an outer-sphere ET reaction, the counteranion mobility often defines the redox kinetics.

However, we believe that the observed electrolyte-dependent PCET kinetics of Ce-MOF-808 cannot be explained, at least completely, by ionic interaction. The reduction of the Ce^4+^ to Ce^3+^ within the node can be charge-compensated by a cation like [TrisH]^+^. Thus, in a pH 7-adjusted Tris buffer enriched in [TrisH]^+^, *D*_app,Red_ should be lower because of the strong ionic interaction. Yet, *D*_app,Red_ in Tris buffers, regardless of the pH, was higher than those measured in MOPS or borate buffers. Furthermore, the buffer-node Coulombic interaction cannot explain the apparent anisotropy between *D*_app,Red_ and *D*_app,Ox_. The change in ionic strength via modulation of buffer concentration also resulted in distinct and nonintuitive trends in PCET kinetics (see SI for details).

Here, we further emphasize that the product of the inner-sphere PCET reaction with equimolar amounts of protons and electrons retains charge neutrality.^2,5,25,41,49,50^ As shown in Scheme 2, the addition of H^+^ to the terminal –OH and electron to Ce^4+^ does not result in a charged product like those that would otherwise form in outer-sphere ET reaction. Thus, it is difficult to conceive how the kinetics of converting a charge-neutral substrate to a charge-neutral product would depend on Coulombic interactions. Overall, ionic interaction seems to play little to no role in defining the PCET kinetics of Ce-MOF-808.

Thus, it is more appropriate to assign the buffer-dependent *D*_app_ values to the *chemical interactions between the buffers and Ce-MOF-808*. We discuss the exact interactions between different buffers and the nodes of Ce-MOF-808 in the next section.

### Experimental and Computational Derivation of Buffer-Node Interactions

In this work, we employed ITC measurements and computational modeling to probe the thermodynamics of buffer-node interactions.

According to the ITC measurements, borate buffer interacted the strongest, followed by Tris, and then MOPS (Table 3 and Figure 3). This is expected, as borate buffers are known to coordinate to surfaces of transition metal oxides strongly, and can even participate in catalytic reactions (cf.^12^). In fact, we employed these three buffers precisely because of the expected difference in coordination thermodynamics with the Ce_6_ nodes of Ce-MOF-808. It was, however, surprising that the borate-node interaction was *entropically driven*. If the reaction is driven by the strong Ce–O-borate bond, then we expect that this reaction should instead be *enthalpically driven.* Our initial computational modeling indicated that when a singly deprotonated borate anion, [H_2_BO_3_]^−^, approached the node, the μ_3_–OH donated the proton to the anion. We acknowledge that this reaction is performed en vacuo with at most one proximal H^_2_^O molecule. Thus, pore-filled H_2_O network is not considered. However, it is still tempting to claim that this release of solid-bound proton to [H_2_BO_3_]^−^ then quickly to H_2_O (through the Grotthuss mechanism) causes the ‘borate-node’ interaction to be entropically favorable. This is reminiscent of entropically driven proton transfer reactions within micropores of zeolites,^55,56^ or between membranes in biological systems.^57,58^

Protonation of [H_2_BO_3_]^−^ alters the acid–base equilibrium, and, effectively, increases the apparent proton activity within the MOF pores. This explains the relatively large deviation of *E*_1/2_ vs NHE of the Ce^4+^O/Ce^3+^OH redox reaction when the borate concentration was altered at a given pH; at buffer concentrations of 0.1–0.2 M, a large fraction of [H_2_BO_3_]^−^ is protonated within the MOF pores, and thus the local pH proximal to the node should be lower than the bulk electrolyte.

Returning to the buffer-node interaction, our computational simulations indicated that Δ*G*_Buf_ solely from borate coordination to the pristine Ce_6_ node is around −0.3 kcal mol^–1^ in its most thermodynamically stable configuration. This relatively thermoneutral Δ*G*_Buf_ suggests that the solubilized, but pore-confined H_3_BO_3_ is in dynamic equilibrium with its node-bound state by displacing the terminal −OH_2_ group. Furthermore, much of the ITC-derived ΔG likely arises from the proton transfer between [H_2_BO_3_]^−^ and the MOF (vide supra). Conversely, when Tris-node interaction was computed, the Δ*G*_Buf_ was more negative (−2 kcal mol^–1^; see Figure 4). ITC-derived ΔH of −6 to −8.7 kcal mol^–1^ also corroborates this simulation.

It is noteworthy that, thermodynamically, the most favorable states of all three buffers resulted in the displacement of *only one terminal −OH/–OH*_*2*_ *group.* Given that carboxylate groups on the organic linker of the MOF bridge between two Ce cations, this may look surprising. Indeed, formate anions of similar size to H_3_BO_3_ can bind to two metal cations within MOF-808 and NU-1000 with similar inorganic nodes.^59,60^ However, recently, Liu et al. have reported that postsynthetically introduced benzoates to the MOF, NU-1000, bind in a similar fashion as H_3_BO_3_ binding to the Ce_6_ node.^51^

ITC measurements can only indicate the interaction between the pristine Ce-MOF-808 and the buffer. Most, if not all, Ce cations within Ce-MOF-808 are in the 4+ oxidation state.^24^ Thus, we turned to computational simulations of buffer-node interaction when the node is reduced with 1H^+^/1e^–^. As described in the Results section, for both Tris and H_3_BO_3_-bound nodes, reduction by 1H^+^/1e^–^ resulted in an increase in Δ*G*_Buf_ by around 3–4 kcal mol^–1^.

The ca. 3–4 kcal mol^–1^ difference between the buffer-binding thermodynamics of oxidized vs reduced node, denoted Δ*G*_Buf,Ox_ or Δ*G*_Buf,Red_, respectively, suggests that under otherwise identical conditions, the oxidized node is ∼150–800 times more likely to be bound to the buffer molecules than the reduced node; see eqs 12 and 13 and the related equilibrium constants, *K*_Buf,Ox_ and *K*_Buf,Red_.

$$\Delta \Delta G_{\mathrm{Buf}}=\Delta G_{\mathrm{Buf},\mathrm{Ox}}-\Delta G_{\mathrm{Buf},\mathrm{Red}}$$ 12

$$\Delta \Delta G_{\mathrm{Buf}}=-1.37\log\left(\frac{K_{\mathrm{Buf},\mathrm{Ox}}}{K_{\mathrm{Buf},\mathrm{Red}}}\right)$$ 13

As described in the next section, it is this difference in buffer affinity that leads to the apparent violation of the principle of microscopic reversibility.

### Anisotropic *D*_app_/*k*_app_ and Apparent Microscopic “Irreversibility”

From here onward, we will employ both *D*_app_ and *k*_app_ values as we begin comparisons of PCET kinetics between Ce-MOF-808 and other redox-active systems beyond MOFs. In this section, we focus on the anisotropy in PCET kinetics of Ce^4+^O/Ce^3+^O–H redox couple.

First, we note that for every electrode and electrolyte, the *E*_CPE_ values were controlled to be ±240 mV with respect to the peak potential of CVs measured prior to any CPE measurements. Thus, in all cases, the additional reaction free energy to drive the PCET reaction should be identical. Yet, as shown in Table 2, *k*_app,Red_ values of Ce^4+^O reduction to Ce^3+^OH in all electrolytes were at least 3–10 times larger than those of the oxidation reaction (*k*_app,Ox_). All chronoamperometric measurements exhibited a rapid decrease in current within the first 5 s, and the measured current plateaued, suggesting that the system has reached an equilibrium. Thus, the observed trend between *k*_app_ and pH suggests that, regardless of the electrolyte, buffer molecules facilitate node reduction and/or hinder oxidation. *At first glance, this appears to violate the principle of microscopic reversibility* (see ref 61 for details on microscopic reversibility). As described below, we can explain this through buffer-node interactions to strictly uphold the thermodynamic principles. Nevertheless, this is particularly interesting, as previously, we have demonstrated that the Ce^3+^O–H BDFE is independent of the buffer and the electrolyte pH.^25^

The previously observed buffer-independent Ce^3+^O–H BDFE does *not* rule out any interaction between the buffer and the node. The square scheme shown below illustrates the PCET reaction of buffer-free and buffer-bound nodes, where ‘Buf’ is used to denote a generic buffer molecule in the electrolyte (Scheme 3). Only one out of six Ce cations is shown for clarity. According to Hess’s Law, the PCET thermodynamics of the buffer-free and buffer-bound nodes would be nearly identical if the buffer-node interaction between oxidized and reduced nodes is similar; in other words, Δ*G*_Buf,Ox_ ≈ Δ*G*_Buf,Red_. As described above, our computational modeling indicates that the difference in these two Δ*G* values was 3–4 kcal mol^–1^; this difference is *more or less* within the typical error range of BDFE, which is ±2 kcal mol^–1^.^7^

**Scheme 3:**
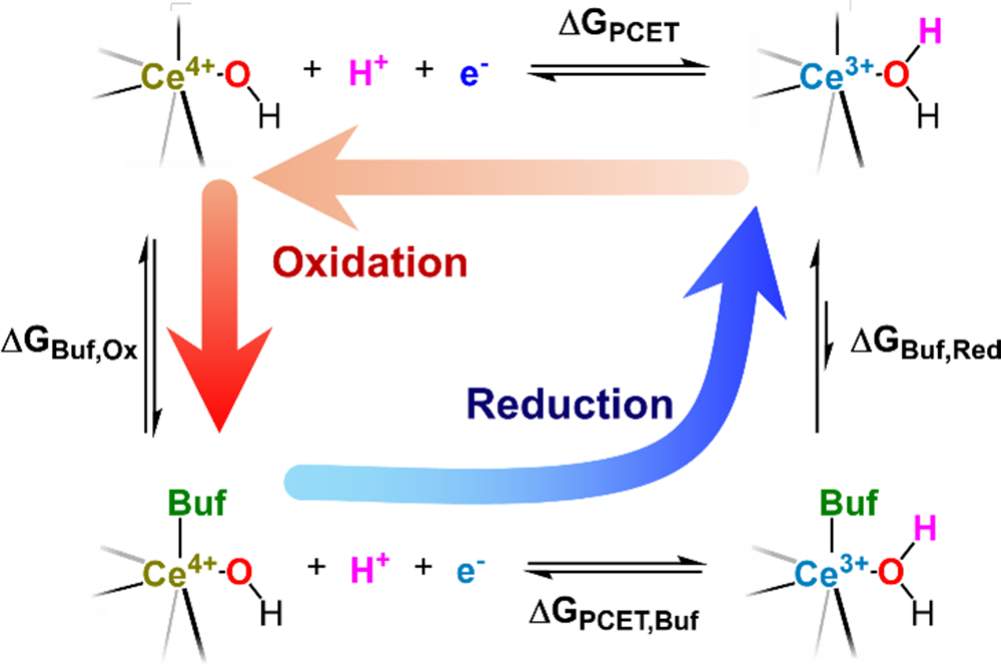
Square Scheme Illustrating the PCET Reaction of Buffer-Free and Buffer-Bound Nodes of Ce-MOF-808

However, this 3–4 kcal mol^–1^ difference between Δ*G*_Buf,Ox_ and Δ*G*_Buf,Red_ can have a more significant effect on PCET hopping kinetics within Ce-MOF-808. Here, we will employ a borate buffer as a case study. Δ*G*_Buf,Ox_ of borate buffer is more likely to be close to zero, even though experimentally, it was exergonic due to the proton transfer between borate and the node (vide supra). Δ*G*_Buf,Red_ is more endergonic. Therefore, when 1H^+^/1e^–^ hops from one Ce cation to another by applying *E*_CPE,c_, the subsequent liberation of node-bound borate to the electrolyte should be thermodynamically favorable, and should occur instantaneously. This reaction is shown as the blue arrow in Scheme 3. In the reverse oxidative reaction, however, the borate binding is more thermoneutral. Thus, this reaction is *not* a ‘simple’ PCET reaction involving a pristine node. Instead, the measured *D*_app,Ox_/*k*_app,Ox_ must be sensitive to both the H^+^/e^–^ hopping and the buffer binding. The two red arrows in Scheme 3 illustrate that during oxidation, at least transiently, the buffer-free, oxidized node forms as an ‘intermediate’ that further undergoes dynamic equilibrium with the buffers in the MOF pore.

Given that Δ*G*_Buf,Ox_ of MOPS buffer was also measured to be nearly thermoneutral experimentally and computationally, the above explanation should hold for the anisotropy observed in MOPS buffer. However, for the Tris buffer, Δ*G*_Buf,Ox_ was measured to be exergonic. The difference between experimental and computational Δ*G*_Buf,Ox_ was roughly 2 kcal mol^–1^. Computationally, we derived Δ*G*_Buf,Red_ to be ∼2 kcal mol^–1^, making ΔΔ*G*_Buf_ to be ∼4 kcal mol^–1^. We have described below in detail that the difference in experimental and computational values is likely due to the lack of explicit solvent molecules in the in silico model (vide infra). However, under otherwise identical conditions, ΔΔ*G*_Buf_ should be more accurate. Together, it is tempting to claim that the ‘true’ Δ*G*_Buf,Red_ is isoergic (i.e., Δ*G*_Buf,Red_ ≈ 0). This would pose the reduction step to be a combination of 1H^+^/1e^–^ addition followed by *slow* buffer release. The buffer binding after removal of 1H^+^/1e^–^ is likely spontaneous.

In essence, the redox reactions of Ce-MOF-808 are *not* purely an electrochemical reaction, but are what would otherwise be called the EC reaction (E = electrochemical, C = chemical). Following an electrochemical PCET reaction, the reduced or oxidized node undergoes a chemical, buffer binding/release. This should, to some extent, cause the ratio of the anodic and cathodic peak currents to deviate from the ideal value of unity. Indeed, we and others have observed this previously for Ce-MOF-808.^24,25^

This square scheme also illustrates the apparent trend between *D*_app_/*k*_app_ and the electrolyte pH. In a pH 8-adjusted borate buffer, for example, the pore must be filled largely with H_3_BO_3_, particularly due to the proton transfer from the node to the small amount of [H_2_BO_3_]^−^ (see above for details). Because the most thermodynamically favorable binding motif of H_3_BO_3_ to the node is through the displacement of terminal −OH_2_, due to Le Chatelier’s Principle, the H_3_BO_3_ binding must be facilitated. Because Δ*G*_Buf,Ox_ is close to thermoneutral, but Δ*G*_Buf,Red_ is endergonic, *k*_app,Red_ should be larger than *k*_app,Ox_ in this electrolyte. At pH 10, the pores are filled instead with [H_2_BO_3_]^−^ and its binding to the node via displacement of terminal −OH is endergonic (see Results section). Thus, in this case, the two *k*_app_ values are more similar than different. For Tris buffers, protonation occurs at the N-atom, which is not the coordination site to the node, and therefore, *k*_app,Red_ is higher than *k*_app,Ox_ for all pHs.

In sum, the apparent microscopic irreversibility can be explained through the difference in buffer-node interaction at different oxidation states. Both the substrate and the product during the reduction or oxidation are *chemically distinct species.*

### Consequences of Electrolyte-Dependent, Anisotropic PCET Kinetics on Linear Free Energy Relationships and Volcano Plots

The Bell–Evans–Polanyi (BEP) relationship that connects the change in reaction thermodynamics (ΔΔ*G*_rxn_) to the change in transition state free energy (ΔΔ*G* ^‡^) has been the basis of kinetic vs thermodynamic relationships of homogeneous and heterogeneous systems (eq 14).^2,11,62,63^ The transfer coefficient, α, that connects the thermodynamic and kinetic parameters, is often assumed to be $\frac{1}{2}$ and, in many cases, electrolyte independent.

$$\Delta \Delta G^{‡}=\alpha \Delta \Delta G_{\mathrm{rxn}}$$ 14

The classical BEP equation can be elaborated to explicitly include *k*_app_ and equilibrium constants of reduction vs oxidation reactions (eq 15). We note that in this equation, the two *k*_app_ and *K*_eq_ values of redox reactions that would otherwise be considered an identical reaction except for the direction of the reaction. In other words, for a microscopically reversible reaction, α should be undefined because the logarithmic components on the two sides of the equation will be zero. We acknowledge that the *k*_app_ values refer to the hopping kinetics, while strictly, instrinsic rate constants should be employed for the BEP relationship. We have described the difficulty in determining accurate intrinsic rate constants above.

$$\log\left(\frac{k_{\mathrm{app},\mathrm{Ox}}}{k_{\mathrm{app},\mathrm{Red}}}\right)\approx \alpha \log\left(\frac{K_{\mathrm{Buf},\mathrm{Ox}}}{K_{\mathrm{Buf},\mathrm{Red}}}\right)$$ 15

For a microscopically irreversible system, α becomes a defined value even between the reductive vs oxidative reaction of a nominally same redox couple. We have previously demonstrated that the Ce^3+^O–H BDFE is largely buffer-independent and is quantitatively similar to those computed (without explicit buffer molecules).^25^ We have also unified the difference between *E*_CPE_ and peak potentials to be 240 mV. Thus, we can assume that buffers have a minimal role in altering PCET thermodynamics—i.e., in Scheme 3, Δ*G*_PCET_ ≈ Δ*G*_PCET,Buf_. Therefore, ΔΔ*G*_rxn_ is essentially dominated by the difference between Δ*G*_Buf,Ox_ vs Δ*G*_Buf,Red_. This difference was ca. 2–4 kcal mol^–1^ (vide supra). The ratios of *k*_app,Ox_ vs *k*_app,Red_ in all buffers were from 1:3 to 1:10. Using these values and combining eqs 13 and 15, the derived α ranges between 0.1 and 0.2. In essence, this α is better denoted as Δα between the reduction vs oxidation reactions. Thus, depending on the direction of the reaction, the measured α may deviate by ±Δα—i.e., assuming an otherwise ideal α of $\frac{1}{2}$ , the actual α of a microscopically irreversible system can range from 0.3 to 0.7.

*We emphasize that this defined* α *for nominally identical redox couples indicates that, depending on the direction of the reaction, the kinetic vs thermodynamic relationship is distinct.* This may be why, for example, many metallic/metal oxide-based systems exhibit α that deviates from $\frac{1}{2}$ in PCET/HAT reactions,^63–65^ though many other reasons are possible (see below).

The reason for this anomalous behavior is rather simple—oxidation and reduction reactions are *chemically distinct reactions*. The BEP relationship assumes a single-step reaction that is of identical chemical nature and is microscopically reversible.^2,11,62,63^ Clearly, this is not true for PCET reactions of Ce-MOF-808.

Despite this rather ‘simple’ reason, the *implicit* assumption that the interfacial reactions in heterogeneous catalysis are microscopically reversible is prevalent. The classical example of this assumption is the volcano plots for HER. Often, monometallic and binary/ternary catalysts like metal sulfides and phosphides are plotted on the same volcano plot, with exchange current density (*j*_0_) used as the metric of their HER activities.^8^ This implies that under an identical driving force, metal sulfides/phosphides can be a competent catalyst for the reverse H_2_ oxidation reaction (HOR). However, experimentally, metal sulfides/phosphides that are *HER-active* are almost always *HOR-inactive*. This apparent catalytic irreversibility is often due to distinct surface-bound H-atoms that are involved in the two reactions. H_2_ dissociation on metal phosphides is heterolytic, yielding strong metal–H bonds, limiting the HOR rate by its bond dissociation. In HER, however, H_2_ likely evolves by involving weakly bound surface-H species either through Heyrovsky or Tafel-like steps.^66,67^ This contrasts with Pt and other monometallic catalysts, where, regardless of the interfacial PCET or H_2_ dissociation, at least nominally, the two reactions yield the same intermediate.^9^ In essence, Ce-MOF-808 behaves similarly to metal sulfides/phosphides. The reactive sites for oxidative vs reductive reaction of the same redox couple are chemically distinct.

We would like to emphasize that ‘microscopic irreversibility’ is not unique to heterogeneous (electro)­catalysts. In fact, many enzymatic active sites, metallocofactors, and active site cascades exhibit bidirectional, but irreversible, electrocatalytic behavior.^68–72^ In many of these cases, proton/electron transfer (PT/ET) pathways are altered at the reduced vs oxidized state. In other words, H^+^’s and e^–^’s hop to different sites in the reduction vs oxidation reaction. Thus, conceptually, this is analogous to the different pathways described in Scheme 3 above for Ce-MOF-808. We cannot rule out that, on top of the above buffer-node interactions, protons and electrons involved in the redox reaction are transferred to and from different moieties in the reduction vs oxidation reaction. However, if the difference in PT/ET pathways in the redox reactions dominates the kinetic anisotropies, we expect PCET kinetics of Ce-MOF-808 at *identical pH* but *distinct buffers* to be similar. However, as shown in Figure 2C,D, this is not true. Thus, we prefer to claim thermodynamically anisotropic buffer-node interactions to be the primary reason for the observed kinetic anisotropy in PCET kinetics.

The observed *pH-dependent* PCET kinetics of Ce-MOF-808 are reminiscent of those reported using graphite-conjugated carboxylic acids by Surendranath and co-workers. In this work, they have demonstrated that carboxylic acids conjugated to a graphitic electrode can exhibit *k*_app_ between >10^4^ and 10 s^–1^, depending on the electrolyte pH;^73^ it is noteworthy that though Ce-MOF-808 is formally an electrical insulator,^24^ the estimated *k*_app_ values are within the same range as the graphite-conjugated acids. This further suggests that Ce_6_ clusters in close proximity to the FTO surface are responsible for the observed electrochemical behavior.

Returning to the graphite-conjugated carboxylic acid, the *k*_app_ vs pH trend can be fitted using the Butler–Volmer equation. Though it is tempting to apply this concept to Ce-MOF-808, we emphasize that the Butler–Volmer equation also assumes the principle of microscopic reversibility. Thus, the observed *k*_app_ vs pH trend should not be explained using the same approach.

We acknowledge that the deviation of α from the ideal value of $\frac{1}{2}$ can be ascribed to many other reasons. In the context of PCET reaction, this deviation is often ascribed to the asynchronous PCET reaction. The proton and electron do not transfer synchronously along the reaction coordinate. There remains controversy on how this phenomenon can be explained quantum-mechanically, and we direct our readers to the following references for more details.^36,74^

Specific to heterogeneous systems, the deviation of α is also often ascribed to site heterogeneity and diffusive complications.^64,65^ We cannot rule out these effects on Ce-MOF-808. Indeed, we have previously demonstrated that Ce_6_ nodes present a distribution in thermodynamics of the PCET reaction (see the last section of the Discussion for more details). Given that the proton source would have to diffuse to Ce_6_ sites near FTO, diffusive complications cannot be ruled out, though the fast current decay in all chronoamperometric curves otherwise suggests minimal diffusive complications.

Despite the complications mentioned above, it is still tempting to claim that at least part of the reason behind the nonideal BEP relationships reported in a wide range of systems may be due to the microscopic irreversibility, like that observed for Ce-MOF-808.

### Implications of Electrolyte-Dependent PCET Hopping Kinetics on (Electro)­catalysis

Our findings demonstrate that much like metal oxides, the PCET hopping kinetics within the pores of Ce-MOF-808 are also electrolyte-dependent. This challenges the implicit but common assumption within the MOF-based catalysis field, where a pristine node from crystallographic data is assumed to represent all catalytic motifs.^75,76^ Instead, at least for Ce-MOF-808 in borate buffer, the borate anions not only can deprotonate the Bro̷nsted acids on the node, but roughly half of the H_3_BO_3_ within the MOF pores likely undergoes coordination with the node, displacing terminal −OH_2_ (vide supra). This reaction is much more endergonic when the node is reduced with 1H^+^/1e^–^. While we demonstrated that these buffer bindings have a relatively small effect on PCET *thermodynamics*, their effect on PCET hopping *kinetics* is profound. The implicit assumption of omitting electrolytes is directly related to the difficulty in understanding their exact molecular arrangement at solid–liquid interfaces. We discuss challenges and limitations related to this difficulty below.

Given the drastic change in rate constants, we advocate that electrolyte compositions should be leveraged as another tool to potentially enhance the (electro)­catalytic performance of MOFs and other heterogeneous materials. MOFs with redox-active motifs have emerged as candidate materials for electrocatalysts in reactions of H_2_, O_2_, and CO_2_, as well as electrodes for energy-storage devices.^77–83^ Often, chemists rely on ‘engineering’ the MOF structure to enhance its electrochemical properties, much like how traditional heterogeneous materials are structurally modified.^84–86^ Given this preconceived notion, we propose the rather significant effect of the electrolyte systems in PCET as additional modulators of heterogeneous (electro)­catalysis.

While recently, the effect of electrolytes within the MOF pores on catalysis has gained attention (cf.^21,29,79^ ), *this work is, to the best of our understanding, the first report quantifying the exact effect of electrolytes in PCET hopping kinetics of MOFs.*

### Limitations Associated with the Presented Studies

We conclude this Discussion section by outlining the limitations of the presented studies. Limitations specific to ITC measurements and electrochemically derived *D*_app_ values specific to MOF-based systems can be found in the following references.^27,28,42,87^

As noted above, the *k*_app_ and the *D*_app_ values presented in this study probe the hopping reaction of protons and electrons within the lattice structure. This is fundamentally distinct from the *intrinsic rate constants* of a PCET reaction. In other words, the measured *k*_app_ is related to the initial Ce^3+^O–H bond cleavage, followed by similar bond formation at another location; this can occur on the same or different Ce_6_ nodes. If the intrinsic rate constants of Ce^3+^O–H bond formation/cleavage were to be measured, Ce-MOF-808 would have to be titrated with chemical reductants or oxidants and probe the reaction progress. We have described previously, however, that these titrations were unsuccessful as Ce-MOF-808 decomposed after exposure to these reagents.^25^

We also have previously demonstrated that the Ce^3+^O–H BDFE of Ce-MOF-808 has a non-negligible distribution.^25^ This may be due to two distinct reasons: proton topologies with intrinsically different BDFEs, or node-linker-node lateral interactions due to the expansion/contraction of Ce cations during the redox reaction (vide supra). From the full-width-half-maximum (fwhm) of the CVs, we concluded that the H-atom adsorption/desorption thermodynamics on Ce-MOF-808 follow the Frumkin isotherm. Thus, the Ce^4+^O/Ce^3+^OH redox potentials are better described using the modified Nernst equation with a linear correction factor, the Frumkin interaction parameter (*C*; eq 16).^41,52^ In this equation, we have used θ to denote fractional surface coverages of H-atoms on *electroactive sites*, i.e., Ce^3+^OH. Thus, 1 – θ refers to Ce^4+^O. For Ce-MOF-808, *C* was estimated to be roughly 6 kcal mol^–1^ (or 0.25 eV).^25^

$$E=E^{{}^{\circ}}-0.059\log\left(\frac{\theta }{1-\theta }\right)-0.059pH+C(\theta -0.5)$$ 16

Kinetic vs thermodynamic relationships using an H-atom donor/acceptor that follows the Frumkin isotherm are challenging. When, for example, *E*_CPE,c_ is applied to Ce-MOF-808, at the early points, most if not all Ce_6_ nodes are fully oxidized, and thus the effect of Frumkin interaction parameter should be low. However, the Frumkin effect will become more prevalent at larger θ. This may be the reason why, for example, we observed deviation from the linear fits at early time points (see Figure 2B as an example), though it is hard to decipher when this effect is most prominent. This further complicates the BEP-type analysis,^11,88^ which, as stated above, is already convoluted due to the buffer-node interaction (vide supra). Together, these complications proved that retaining *E*_CPE_ large enough to drive all possible reactions and monitoring apparent kinetics is the most adequate method.

We also acknowledge that the computational simulations employed a single, formate-terminated Ce_6_ cluster with buffer molecules under vacuum (with an implicit solvent model included), or at most one H_2_O molecule. In our previous work, the computed and experimentally derived Ce^3+^O–H BDFE were quantitatively similar;^25^ much like molecular species, the addition of 1H^+^/1e^–^ does not cause a significant change to the arrangement of electrolytes surrounding the Ce_6_ cluster within the MOF. Thus, even though the computed model omitted, for example, H_2_O molecules within the MOF pores, the two values quantitatively agreed with each other. For buffer molecules, however, the rearrangement of solvent molecules before and after buffer-node interaction must be more significant.

Explicit computational simulations of multiple H_2_O molecules during electrochemical reactions remain a significant challenge within this field. In fact, this is one of many reasons why the PCET thermodynamics on conventional heterogeneous electrodes is often performed en vacuo,^8^ like that reported here and in our previous reports.^25,49,89^ This is even more challenging when a microporous environment must be considered. H_2_O molecules confined within a nanosized space form a hydrogen-bonded network that is distinct from the bulk solution. This further induces differences in proton activity, dielectric constants, and many other factors that can influence the PCET reactivity.^20,90,91^ In fact, to some extent, we have experimentally and computationally demonstrated that [H_2_BO_3_]^−^, which remains deprotonated in bulk solution, likely becomes protonated inside the pores of Ce-MOF-808 (vide supra).

While we acknowledge many challenges that remain to be addressed, this work quantitatively demonstrated that the anisotropic inner-sphere PCET kinetics are intrinsically correlated to the electrolyte composition.

## Conclusions

Kinetics of PCET reaction and its hopping within the porous lattice of Ce-MOF-808 were examined through the electrochemically derived *D*_app_ values. These values are directly correlated to the *k*_app_ of proton/electron hopping either within one node or between multiple nodes. Our results demonstrated that these values were intimately correlated to the direction of the reaction, as well as electrolyte compositions and pH. ITC and computational simulations that probed the thermodynamics of buffer-node interactions indicated that both the redox states of the node and the buffer identity define the reaction free energy, which leads to the “apparent microscopic irreversibility” in the PCET reaction of Ce-MOF-808.

The PCET kinetics of Ce-MOF-808 were observed to change by orders of magnitude by changing the electrolyte identity and/or the proton activity. This raises questions about the common assumptions within the MOF-based (electro)­catalysis field that computational simulations performed in vacuum or with *implicit solvent* can accurately represent ‘true’ kinetics of the MOF-catalyzed reaction. This is primarily related to the difficulty in treating H_2_O molecules within the MOF pores explicitly, and we have also faced this difficulty in simulating the thermodynamics of buffer-binding in this work. Still, this work sheds light on electrolytes being an important component in designing MOF-based electrochemical/electrocatalytic systems. Currently, our research focus is to explore this buffer-dependent kinetic anisotropy of Ce-MOF-808 in electrocatalytic conversion of small molecules, and to explore other MOFs with Ce and various other metal-oxo clusters.

The electrolyte-dependent PCET kinetics of Ce-MOF-808 implies that kinetic parameters, such as reaction rates in volcano plots, can exhibit significant distribution. We and others have previously demonstrated that thermodynamic parameters like BDFEs on metallic surfaces, metal oxides, metal phosphides, and even MOFs can exhibit a wide distribution.^16,17,41,50,52,92^ This contradicts the common assumption that BDFEs are single-valued in nature. We elaborate on these findings that kinetic parameters may also *not be a single value* in nature. We advocate that this distribution in PCET kinetics should be treated as an advantage to tune the activity of electrodes in energy storage and conversion to optimize the reaction kinetics.

These findings also raise questions about whether the observed distribution in PCET kinetics is intrinsic to Ce-MOF-808. Our current research focuses on examining similar PCET kinetics over a wide range of redox-active MOFs, which overall should construct a comprehensive, kinetic vs thermodynamic relationships relevant to energy storage and conversion. This should lay the foundation to revise the preconceived notions of volcano plots, considering that kinetics are single-valued and are electrolyte-independent.

## Supplementary Material



## Notes

Details on characterization and computational simulations of Ce-MOF-808 (PDF)
