# Supplementary material for: Electrolyte-Dependent, “Microscopically Irreversible” H‑Atom Transfer Kinetics of Ce-Based Metal–Organic Framework, Ce-MOF-808
Source: ACS Appl Mater Interfaces. 2026 Jan 5;18(1):1741–55. doi: 10.1021/acsami.5c21367 (PMC12781061; doi:10.1021/acsami.5c21367)
Supplement: Supplementary file 1 [file am5c21367_si_001.pdf]

Supporting Information

Electrolyte-Dependent, “Microscopically Irreversible” H-atom Transfer Kinetics of Ce-Based Metal-Organic Framework, Ce-MOF-808

Miguel A. Liuzzi-Vaamonde,[†] Zaheer Masood,[‖] Bin Wang,[‖] Nikolay V. Tkachenko[†] and Hyunho Noh[†*]*

[†]Department of Chemistry and Biochemistry, University of Oklahoma, Norman, OK 73019, USA

[‖]School of Chemical, Biological and Materials Engineering, University of Oklahoma, Norman, Oklahoma 73019, United States

*Corresponding authors

Nikolay V. Tkachenko: nikolay.tkachenko@ou.edu

Hyunho Noh: hyunho.noh-1@ou.edu

Table of Contents

1 General Considerations 3
1.1 Materials 3
1.2 Instrumentation 3
2 Experimental Details 4
2.1 Synthesis of Ce-MOF-808 nanocrystals 4
2.2 Preparation of Ce-MOF-808|FTO Electrodes 5
3 Physical Characterization of Ce-MOF-808 5
3.1 N_2 Adsorption-Desorption Isotherm 5
3.2 PXRD Patterns 6
3.3 SEM Images 6
3.4 Details on Zeta-Potential (E(ζ)) Measurements 6
4 Calculation of Apparent Electron Diffusion Coefficients (D_{app}) of Ce-MOF-808|FTO 7
4.1 Details on CV measurements 7
4.2 Scan rate dependence 10
4.3 Details on CPE measurements 18
4.4 Details on Cottrell Analysis of CPE Data 19
4.5 Investigation of Redox Anisotropy between $D_{app,Ox}$ and $D_{app,Red}$ 28
5 Investigation of Ce-MOF-808–Buffer Interactions 30
5.1 Details on Isothermal Titration Calorimetry 31
6 Computational Details 36
6.1 Details on Computational Methods 36
6.2 Proton Transfer between Buffer and Ce_6 Nodes 38
6.3 Coordination of Buffer to Ce_6 Nodes 40
6.4 XYZ Coordinates 45
7 References 95

1 General Considerations

1.1 Materials

All chemicals and materials in this work were used as received.

Fluorine-doped tin oxide glass (FTO; surface resistivity ~7 Ω sq^{-1}), ammonium cerium(IV) nitrate (99+%), 1,3,5-benzenetricarboxylic acid (H_3BTC; 95%), formic acid (≥88%), 3-(N-Morpholino)propanesulfonic acid (MOPS; ≥99.5%), boric acid (H_3BO_3; ≥99.5%), and sodium chloride (≥99.0%) were purchased from Sigma-Aldrich. N,N'-Dimethylformamide (≥99.8%) was received from Supelco. Concentrated hydrochloric acid and tris(hydroxymethyl)aminomethane (Tris) were received from Fisher Scientific. Sodium hydroxide was purchased from Macron Chemicals.

All aqueous buffers (MOPS, Tris, and H_3BO_3) were prepared using 18.2 MΩ cm water (Millipore Synergy Water Purification System). Concentrations of NaCl were kept constant, at 0.1 M for all buffers, while the concentrations of buffer species were adjusted between 0.1 and 1.0 M as indicated for each measurement. The pH of the aqueous electrolytes was further adjusted using >5 M NaOH or HCl.

D_2O (99.9 atom % D) and sodium deuteroxide (40 wt% in D_2O, 99+ atom % D) used for ^1H NMR were purchased from Sigma-Aldrich.

1.2 Instrumentation

N_2 adsorption-desorption isotherm of Ce-MOF-808 was measured using 3Flex (Micromeritics) to confirm the porosity of the material. Before all measurements, freshly prepared Ce-MOF-808 was thermally activated under a dynamic vacuum (<50 mTorr) at 80 °C using VacPrep (Micromeritics) following the reported procedure.[1] Brunauer–Emmett–Teller (BET) area of Ce-MOF-808 was determined to be 1500 m^2 g^{-1} using the data set between P/P_0 = 0.005 – 0.1.[2] The adsorption-desorption isotherm and the DFT-derived pore size distribution are shown below (Figure S1). This corresponds well with the previously reported results.[3-5]

The grazing incidence powder X-ray diffraction (PXRD) patterns were collected using the Rigaku Miniflex600 equipped with a Ni-filtered Cu Kα X-ray source. 2θ between 2 – 15° with a step size of 0.02°/min were employed as parameters.

Zeta-potential (E(ζ)) measurements were performed using a Malvern Zetasizer Ultra. Aqueous MOF suspensions were prepared by adding solid Ce-MOF-808 in various pH-adjusted (7 to 10) aqueous buffers—MOPS, Tris, and boric acid—and concentrations—0.1 M to 1.0 M—for determining the surface charge.

Electrochemical measurements were performed with a CH Instruments potentiostat (model 600D). The electrochemical system consisted of a Ce-MOF-808 on FTO as a working electrode, a platinum wire auxiliary electrode, and an Ag/AgCl (3M KCl) reference electrode (BASi). Cyclic Voltammetry (CV) and Controlled Potential Electrolysis (CPE) were measured in a single-

chamber electrolytic cell filled with pH-adjusted (7 to 10) aqueous buffers—MOPS, Tris, and boric acid—and concentrations—0.1 M to 1.0 M. CV were measured using a scan rate (υ) of 25 mV s^{-1} unless otherwise noted. All measured potentials were standardized to the Normal Hydrogen Electrode (NHE). All error bars presented throughout this study represent 1σ from triplicate measurements.

All buffers used were adjusted for pH using the SevenDirect SD20 with the InLab® Expert Pro-ISM sensor from Mettler-Toledo. Prior to all measurements, a standard four-point calibration was done using buffers at pH 1.68 from Sigma Aldrich, and pH 4.01, 7.00, and 10.01, which were purchased from Oakton.

Scanning electron microscopy (SEM) images were collected using the TFS Quattro S ESM field emission instrument operated at 20.00 kV. Prior to the imaging, Ce-MOF-808 was dispersed in acetone and drop-casted onto FTO to measure the effective concentration of material in the glass electrode.

Isothermal Titration Calorimetry (ITC) measurements were performed using a Malvern MicroCal PEAQ-ITC system. After each measurement, a standard automated washing procedure was followed using a 20% Contrad 70 solution, followed by multiple ultrapure water rinses and a final methanol (HPLC grade) rinse, and dried under vacuum. ITC data was processed using the Malvern MicroCal PEAQ-ITC Analysis Software compatible with the titration instrument. Measurement details provided below.

Electrochemical impedance spectroscopy (EIS) was measured using Solartron 1400. The collected EIS were analyzed using the software, Z-view.

2 Experimental Details

2.1 Synthesis of Ce-MOF-808 nanocrystals

Ce-MOF-808 was synthesized following a modification of the reported procedure.[3,6] First, a mixture of 67.2 mg of H_3BTC, 4.12 mL of formic acid, and 1.6 mL of DMF were sonicated with 1.2 mL of aqueous ammonium cerium(IV) nitrate (0.53 M) in a sealed 2 dram glass vial for 5 min. Thereafter, the vial was placed into an oven at 100°C for 20 min for crystal growth and readily cooled in a water bath at room temperature. The mother liquor was removed by centrifugation (7830 rpm, 25°C, 10 min). The material underwent the following washing procedure: 3 x DMF (15 mL) and 3 x acetone (15 mL), with immersion periods of 2 h, overnight, and 2 h between each washing step to ensure complete solvent exchange. The material was dried in a vacuum oven at 80°C for 2 h to obtain the Ce-MOF-808 nanocrystals. The MOF was stored at room temperature under N_2 atmosphere.

The porosity and crystallinity were confirmed via N_2 sorption isotherms and powder X-ray diffraction (PXRD) respectively.

2.2 Preparation of Ce-MOF-808|FTO Electrodes

Electrodes of Ce-MOF-808 were prepared as reported previously.[3] A standard drop-casting method was used to deposit MOF nanocrystals on FTO glass electrodes, controlling the area of the exposed electroactive surface to 0.5 × 0.5 cm^2 using polyimide tape. These working electrodes, referred to as Ce-MOF-808|FTO, were loaded with a specific amount of material by preparing a suspension with 6.0 mg of Ce-MOF-808 in 0.5 mL of acetone and drop-casting 4 × 6 µL of the suspension on each electrode. For cross-sectional consistency, fresh electrodes were prepared before each measurement set following the protocol outlined above.

3 Physical Characterization of Ce-MOF-808

3.1 N_2 Adsorption-Desorption Isotherm

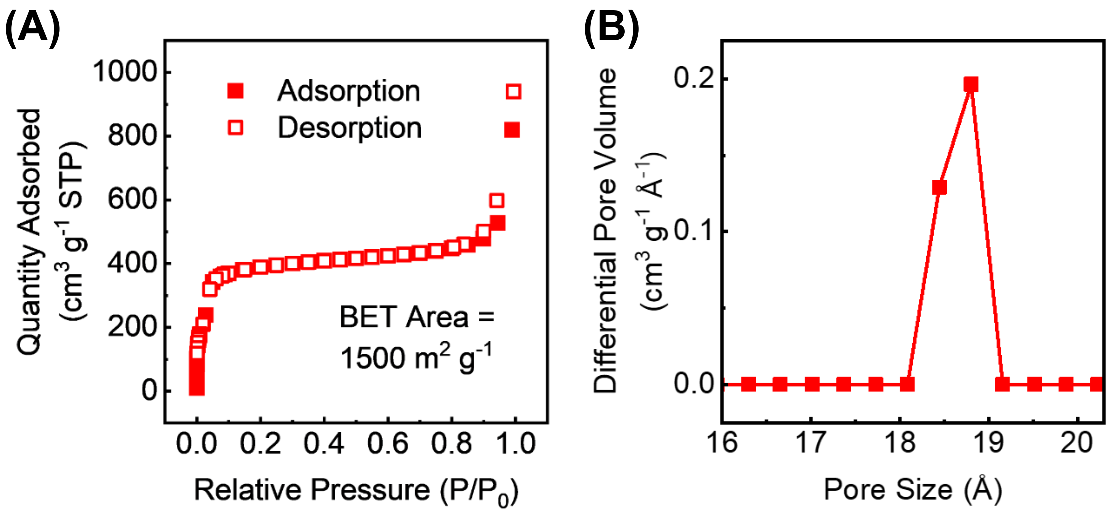

Figure S1. (**A**) N_2-adsorption-desorption isotherms and (**B**) DFT-derived pore size distribution of Ce-MOF-808.

3.2 PXRD Patterns

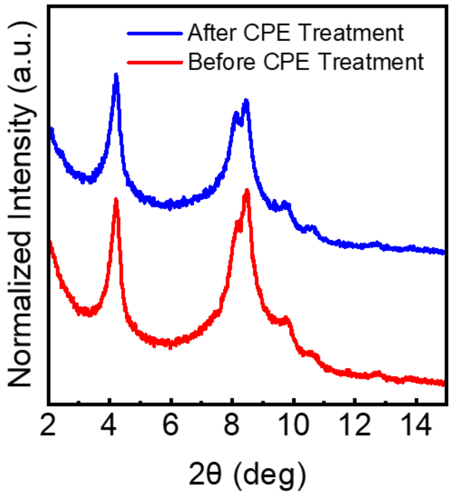

Figure S2. PXRD patterns of Ce-MOF-808|FTO before and after CPE.

3.3 SEM Images

The cross-sectional SEM images shown below were used to estimate the thickness of the Ce-MOF-808|FTO. As noted in *Section 4.4*, this film thickness was used to estimate the effective concentration of Ce cations within the film.

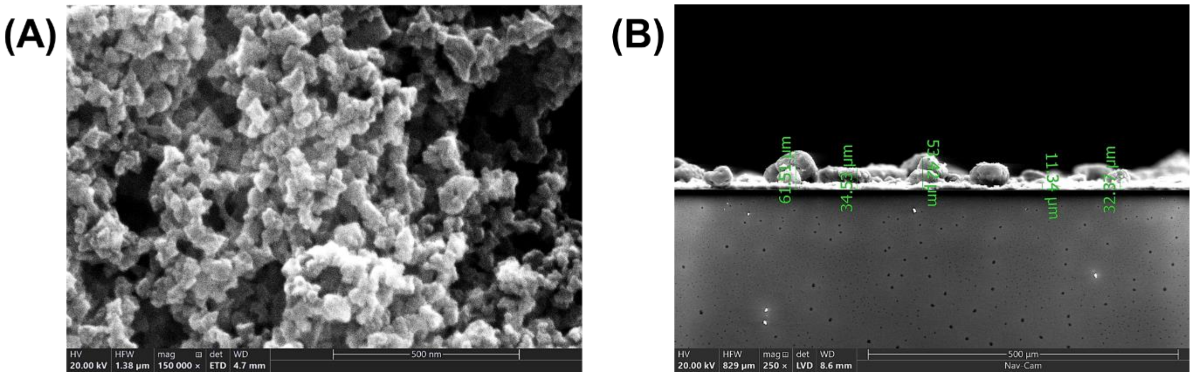

Figure S3. Representative **(A)** Top-down and **(B)** cross-sectional SEM images of Ce-MOF-808|FTO.

3.4 Details on Zeta-Potential (E(ζ)) Measurements

Surface charges were examined using E(ζ) measurements under different pH conditions (7 to 10), buffer types (MOPS, Tris, boric acid), and concentrations (0.1 to 1.0 M). Due to the nature of Ce-MOF-808, a colloidal mixture was impossible to attain while keeping the structural integrity of the MOF; hence, the observed E(ζ) values serve as a relative measure of the overall particle charge and are expected to be considerably low for the rapidly precipitating suspension. Overall, E(ζ)

showed limited stability (E(ζ) = ± 20), with values becoming more neutral (approaching 0 mV) at higher buffer concentrations across all electrolyte conditions tested (Figure S4, Table S1).

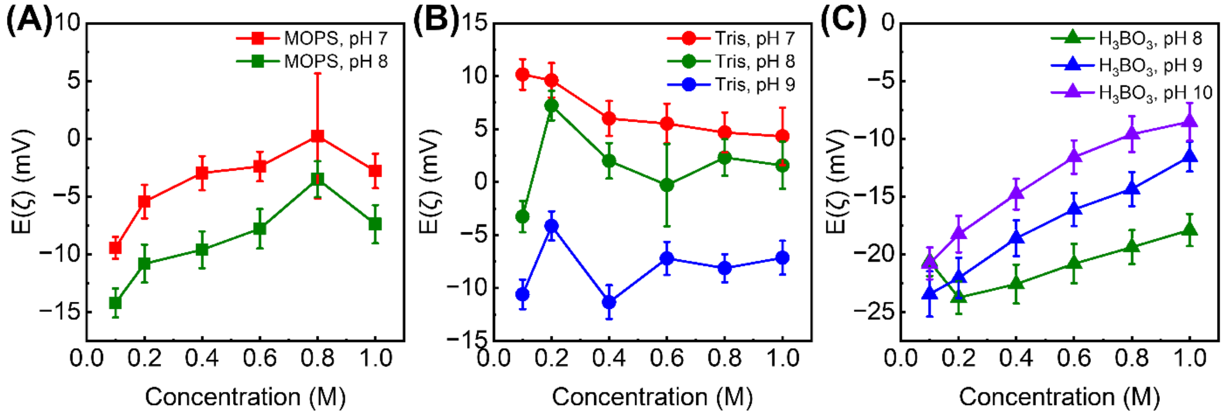

Figure S4. E(ζ) distribution of Ce-MOF-808 in **(A)** MOPS, **(B)** Tris, and **(C)** boric acid aqueous buffers used for electrochemical measurements.

Table S1. E(ζ) values of Ce-MOF-808 dispersions in various aqueous buffers.

Buffer	Concentration (M)	pH 7	pH 8	pH 9	pH 10
MOPS	0.1	–9(1)	–14(1)	–	–
	0.2	–5(1)	–11(2)	–	–
	0.4	–3(1)	–10(2)	–	–
	0.6	–2(1)	–8(2)	–	–
	0.8	0(5)	–3(2)	–	–
	1.0	–3(2)	–7(2)	–	–
Tris	0.1	10(1)	–3(1)	–11(1)	–
	0.2	10(2)	7(1)	–4(1)	–
	0.4	6(2)	2(2)	–11(2)	–
	0.6	6(2)	0(4)	–7(2)	–
	0.8	5(2)	2(2)	–8(1)	–
	1.0	4(3)	2(2)	–7(2)	–
H_3BO_3	0.1	–	–21(1)	–23(2)	–21(1)
	0.2	–	–24(1)	–22(2)	–18(2)
	0.4	–	–23(2)	–19(2)	–15(1)
	0.6	–	–21(2)	–16(1)	–12(1)
	0.8	–	–19(1)	–14(1)	–10(2)
	1.0	–	–18(1)	–12(1)	–9(2)

4 Calculation of Apparent Electron Diffusion Coefficients (D_{app}) of Ce-MOF-808|FTO

4.1 Details on CV measurements

CVs of Ce-MOF-808|FTO were used to determine the cathodic and anodic peak potentials of the reversible Faradaic features corresponding to the $Ce^{4+}O/Ce^{3+}OH$ redox pair transformation. These

potentials are referred to as $E_{CPE,a}$ and $E_{CPE,c}$ in the main text; representative CV with $E_{CPE,a}$ and $E_{CPE,c}$ values are shown in Figure 2A of the main text. This process was repeated for various aqueous electrolytes—MOPS, Tris, and boric acid—over a pH range of 7 to 10 and buffer concentrations of 0.1 to 1 M (Figure S5). Below are the representative CVs of Ce-MOF-808|FTO measured in the electrolytes used for all CPE measurements. Table S2 summarizes the $E_{½}$, full-width-half-maximum (FWHM), peak separations (ΔE_p), and the ratio of the anodic and cathodic peak currents ($j_{p,a}/j_{p,c}$). Here onwards, all errors shown in tables and figures are 1σ of triplicate measurements, unless otherwise noted.

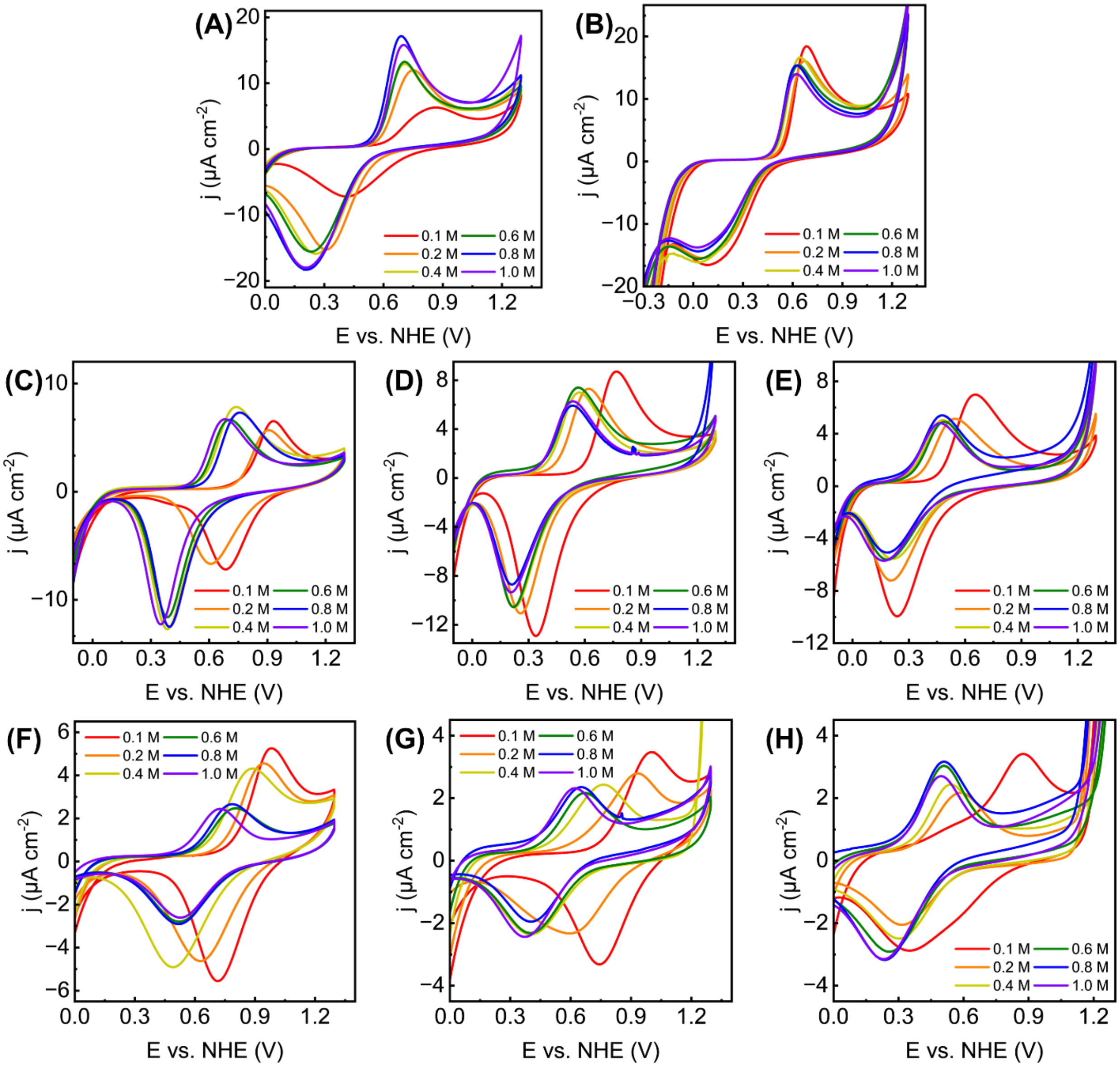

Figure S5. Representative CVs of Ce-MOF-808|FTO in pH-adjusted aqueous buffers of various concentrations. (**A**) MOPS pH 7, (**B**) MOPS pH 8, (**C**) Tris pH 7, (**D**) Tris pH 8, (**E**) Tris pH 9, (**F**) boric acid pH 8, (**G**) boric acid pH 9, (**H**) boric acid pH 10.

Table S2. $E_{½}$, FWHM, ΔE_p, and $j_{p,a}/j_{p,c}$ values of Ce-MOF-808|FTO redox waves from CVs measured in systematically distinct electrolyte media

Buffer	pH	Concentration (M)	$E_{½}$ (V vs. NHE)	FWHM (V)	ΔE_p (V)	$j_{p,a}$ / $j_{p,c}$
MOPS	7	0.1	0.61(2)	0.50(3)	0.41(2)	0.78(8)
		0.2	0.51(2)	0.43(1)	0.44(3)	0.81(4)
		0.4	0.49(1)	0.43(1)	0.44(1)	0.83(2)
		0.6	0.47(1)	0.44(1)	0.45(1)	0.87(2)
		0.8	0.47(2)	0.45(1)	0.45(1)	0.8(1)
		1.0	0.47(1)	0.45(1)	0.47(1)	0.9(1)
	8	0.1	0.44(2)	0.48(2)	0.49(3)	1.1(1)
		0.2	0.42(1)	0.48(5)	0.50(3)	1.1(1)
		0.4	0.39(1)	0.39(3)	0.49(3)	1.1(1)
		0.6	0.38(2)	0.5(1)	0.49(2)	1.1(1)
		0.8	0.37(1)	0.49(7)	0.49(2)	1.1(1)
		1.0	0.37(1)	0.46(9)	0.48(3)	1.0(1)
Tris	7	0.1	0.79(3)	0.41(1)	0.25(1)	0.88(3)
		0.2	0.73(2)	0.43(1)	0.31(1)	0.86(2)
		0.4	0.62(6)	0.43(3)	0.37(2)	0.7(1)
		0.6	0.58(3)	0.40(1)	0.35(3)	0.58(1)
		0.8	0.56(2)	0.41(1)	0.35(1)	0.58(2)
		1.0	0.51(2)	0.41(1)	0.34(1)	0.55(2)
	8	0.1	0.53(4)	0.42(1)	0.39(4)	0.66(3)
		0.2	0.45(1)	0.43(1)	0.38(2)	0.65(2)
		0.4	0.40(1)	0.42(1)	0.34(1)	0.63(3)
		0.6	0.39(1)	0.38(4)	0.34(1)	0.67(4)
		0.8	0.37(1)	0.43(4)	0.32(1)	0.69(5)
		1.0	0.36(1)	0.47(3)	0.31(1)	0.69(2)
	9	0.1	0.44(2)	0.45(1)	0.39(4)	0.76(6)
		0.2	0.38(1)	0.46(5)	0.31(2)	0.8(1)
		0.4	0.36(1)	0.46(1)	0.27(2)	0.90(3)
		0.6	0.35(1)	0.41(4)	0.27(3)	0.91(3)
		0.8	0.33(1)	0.44(1)	0.28(1)	0.9(1)
		1.0	0.32(1)	0.44(1)	0.29(1)	0.85(2)

H_3BO_3	8	0.1	0.85(1)	0.43(1)	0.25(1)	0.98(4)
		0.2	0.78(1)	0.43(1)	0.30(1)	1.1(1)
		0.4	0.70(3)	0.46(5)	0.34(4)	0.94(7)
		0.6	0.65(1)	0.48(1)	0.25(2)	0.88(1)
		0.8	0.64(1)	0.48(2)	0.23(3)	0.90(2)
		1.0	0.63(1)	0.44(2)	0.23(3)	0.89(5)
	9	0.1	0.86(3)	0.44(2)	0.24(1)	1.1(1)
		0.2	0.76(3)	0.55(3)	0.31(2)	1.2(1)
		0.4	0.57(2)	0.47(2)	0.31(4)	0.99(4)
		0.6	0.54(1)	0.44(5)	0.26(2)	0.95(2)
		0.8	0.52(1)	0.45(1)	0.24(1)	1.0(2)
		1.0	0.49(2)	0.45(5)	0.23(2)	0.91(5)
	10	0.1	0.62(1)	0.59(1)	0.49(2)	1.2(1)
		0.2	0.46(1)	0.41(6)	0.28(5)	0.9(2)
		0.4	0.42(1)	0.44(1)	0.23(1)	1.1(1)
		0.6	0.39(1)	0.48(7)	0.24(1)	1.4(1)
		0.8	0.38(1)	0.43(3)	0.27(1)	0.94(5)
		1.0	0.36(1)	0.46(4)	0.25(1)	0.86(1)

4.2 Scan rate dependence

This section describes CVs measured in various scan rates, ranging between 10-100 mV s^{-1} in various buffer systems—MOPS, Tris, boric acid—under different concentrations (0.1 to 1.0 M) and pHs (7 to 9). Representative CVs in each scan rate are shown in Figure S6. Table S3 summarizes the $E_{½}$, ΔE_p, and the ratio of the anodic and cathodic peak currents ($j_{p,a}/j_{p,c}$).

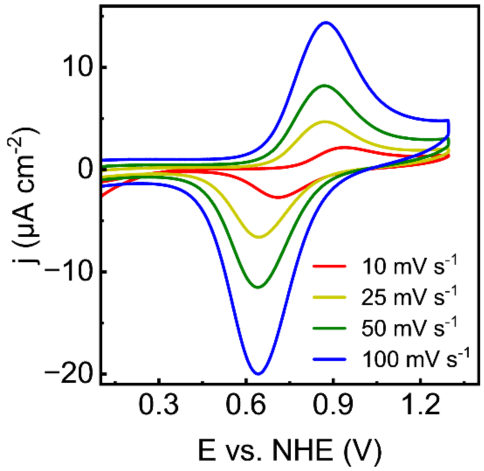

Figure S6. Representative CVs of Ce-MOF-808|FTO in pH 7-adjusted 0.1 M Tris buffer in various scan rates.

Table S3. $E_{½}$, ΔE_p, and $j_{p,a}/j_{p,c}$ values of Ce-MOF-808|FTO redox waves from CVs measured in various electrolytes.

Buffer	pH	Concentration (M)	Scan Rates (mV s^{-1})	$E_{½}$ (V vs. NHE)	ΔE_p (V)	$j_{p,a}/j_{p,c}$
MOPS	8	0.1	10	0.45(4)	0.4(1)	0.9(2)
			25	0.48(2)	0.28(6)	0.84(7)
			50	0.51(2)	0.25(5)	0.93(8)
			100	0.54(2)	0.21(4)	1.00(5)
		0.2	10	0.46(3)	0.3(1)	0.8(1)
			25	0.49(2)	0.23(5)	0.85(4)
			50	0.52(2)	0.21(3)	0.93(4)
			100	0.54(1)	0.19(3)	1.00(2)
		0.4	10	0.46(4)	0.3(1)	0.79(9)
			25	0.50(2)	0.25(5)	0.87(4)
			50	0.52(2)	0.22(4)	0.98(3)
			100	0.54(2)	0.19(4)	1.05(2)
		0.6	10	0.46(5)	0.3(1)	0.74(6)
			25	0.49(4)	0.22(7)	0.81(2)
			50	0.51(2)	0.19(5)	0.90(3)
			100	0.53(1)	0.17(3)	0.96(4)
		0.8	10	0.45(7)	0.3(1)	0.80(5)
			25	0.50(3)	0.21(8)	0.90(5)
			50	0.52(1)	0.18(4)	1.00(1)
			100	0.54(1)	0.15(2)	1.06(2)
		1.0	10	0.45(6)	0.3(1)	0.81(5)
			25	0.48(3)	0.21(8)	0.87(3)
			50	0.50(2)	0.19(6)	0.94(1)
			100	0.52(1)	0.17(4)	1.00(1)

Tris	7	0.1	10	0.74(8)	0.24(1)	0.7(1)
			25	0.71(4)	0.23(1)	0.68(6)
			50	0.72(4)	0.23(1)	0.68(3)
			100	0.72(3)	0.23(1)	0.70(1)
		0.2	10	0.67(7)	0.28(5)	0.8(2)
			25	0.65(4)	0.25(2)	0.78(5)
			50	0.66(3)	0.24(1)	0.79(3)
			100	0.66(3)	0.24(2)	0.80(2)
		0.4	10	0.59(6)	0.27(6)	0.70(2)
			25	0.57(3)	0.24(2)	0.74(2)
			50	0.58(3)	0.23(1)	0.78(2)
			100	0.59(2)	0.23(1)	0.81(2)
		0.6	10	0.53(5)	0.27(6)	0.6(2)
			25	0.53(2)	0.24(2)	0.7(1)
			50	0.54(3)	0.24(2)	0.7(2)
			100	0.55(2)	0.23(1)	0.79(9)
		0.8	10	0.48(3)	0.26(5)	0.63(3)
			25	0.48(1)	0.24(1)	0.72(3)
			50	0.50(1)	0.24(1)	0.79(2)
			100	0.51(1)	0.24(1)	0.84(1)
		1.0	10	0.47(2)	0.26(5)	0.65(5)
			25	0.47(1)	0.24(1)	0.75(4)
			50	0.48(1)	0.23(1)	0.82(2)
			100	0.50(1)	0.22(1)	0.87(1)

Tris	8	0.1	10	0.44(4)	0.3(1)	0.78(5)
			25	0.45(1)	0.21(3)	0.89(7)
			50	0.46(1)	0.19(2)	0.98(8)
			100	0.48(1)	0.17(3)	1.06(8)
		0.2	10	0.40(2)	0.25(6)	0.82(5)
			25	0.41(1)	0.21(2)	0.93(3)
			50	0.43(1)	0.19(1)	1.00(2)
			100	0.44(1)	0.17(1)	1.06(2)
		0.4	10	0.37(1)	0.27(2)	0.76(1)
			25	0.39(1)	0.26(1)	0.88(2)
			50	0.40(1)	0.24(1)	0.95(1)
			100	0.42(1)	0.22(1)	1.00(1)
		0.6	10	0.35(1)	0.26(1)	0.73(1)
			25	0.36(1)	0.25(1)	0.86(1)
			50	0.38(1)	0.23(1)	0.93(1)
			100	0.39(1)	0.22(1)	0.98(1)
		0.8	10	0.35(1)	0.28(1)	0.72(1)
			25	0.36(1)	0.26(1)	0.84(1)
			50	0.38(1)	0.24(1)	0.92(1)
			100	0.39(1)	0.22(1)	0.98(1)
		1.0	10	0.34(1)	0.27(1)	0.71(1)
			25	0.36(1)	0.26(1)	0.84(1)
			50	0.38(1)	0.23(1)	0.92(1)
			100	0.39(1)	0.21(1)	0.97(1)

Tris	9	0.1	10	0.40(2)	0.19(8)	1.0(2)
			25	0.41(1)	0.13(3)	1.11(8)
			50	0.42(1)	0.12(1)	1.14(4)
			100	0.43(1)	0.12(1)	1.16(1)
		0.2	10	0.38(4)	0.2(1)	0.9(2)
			25	0.38(2)	0.14(2)	1.10(8)
			50	0.39(1)	0.12(1)	1.16(3)
			100	0.40(1)	0.12(1)	1.17(1)
		0.4	10	0.35(3)	0.3(1)	0.99(2)
			25	0.36(1)	0.16(2)	1.14(3)
			50	0.37(1)	0.15(1)	1.16(1)
			100	0.38(1)	0.15(1)	1.16(1)
		0.6	10	0.33(2)	0.19(4)	1.06(8)
			25	0.34(1)	0.15(1)	1.08(1)
			50	0.35(1)	0.14(1)	1.11(1)
			100	0.36(1)	0.13(1)	1.13(1)
		0.8	10	0.32(2)	0.19(5)	1.00(1)
			25	0.33(1)	0.15(1)	1.06(2)
			50	0.34(1)	0.14(1)	1.09(1)
			100	0.35(1)	0.13(1)	1.12(1)
		1.0	10	0.31(2)	0.22(4)	1.1(2)
			25	0.32(1)	0.18(1)	1.05(2)
			50	0.33(1)	0.16(1)	1.06(1)
			100	0.34(1)	0.15(1)	1.08(1)

H_3BO_3	8	0.1	10	0.7(1)	0.20(5)	1.0(1)
			25	0.7(1)	0.18(6)	1.01(2)
			50	0.73(9)	0.16(5)	1.02(6)
			100	0.71(4)	0.13(1)	1.05(5)
		0.2	10	0.7(1)	0.22(7)	1.0(1)
			25	0.70(8)	0.18(6)	0.98(9)
			50	0.70(7)	0.16(4)	1.0(1)
			100	0.66(1)	0.08(8)	0.9(4)
		0.4	10	0.66(5)	0.2(1)	1.3(7)
			25	0.64(1)	0.14(3)	0.93(3)
			50	0.65(1)	0.12(2)	0.99(2)
			100	0.65(1)	0.12(1)	1.03(1)
		0.6	10	0.61(2)	0.14(6)	0.82(4)
			25	0.61(1)	0.11(1)	0.90(1)
			50	0.62(1)	0.09(1)	0.94(1)
			100	0.62(1)	0.09(1)	0.97(1)
		0.8	10	0.62(1)	0.14(5)	0.8(2)
			25	0.62(1)	0.11(1)	0.85(3)
			50	0.62(1)	0.10(1)	0.90(1)
			100	0.62(1)	0.10(1)	0.94(1)
		1.0	10	0.62(1)	0.13(4)	0.87(7)
			25	0.62(1)	0.10(1)	0.93(2)
			50	0.63(1)	0.10(1)	0.96(1)
			100	0.63(1)	0.10(1)	0.99(1)

The log of peak currents ($i_{p,a}$ and $i_{p,c}$) was plotted against the log of scan rates (υ), and the slope of the plots indicates whether the redox reaction is controlled by diffusion or kinetics of the PCET reaction at Ce-MOF-808|FTO. This was performed in all electrolytes described above. As shown in Figures S7-S11, the slopes of these plots range between 0.5 to 1.0, suggesting that the redox mechanism depends on the electrolyte. The peak currents and the slopes of the linear fits are shown in Table S4.

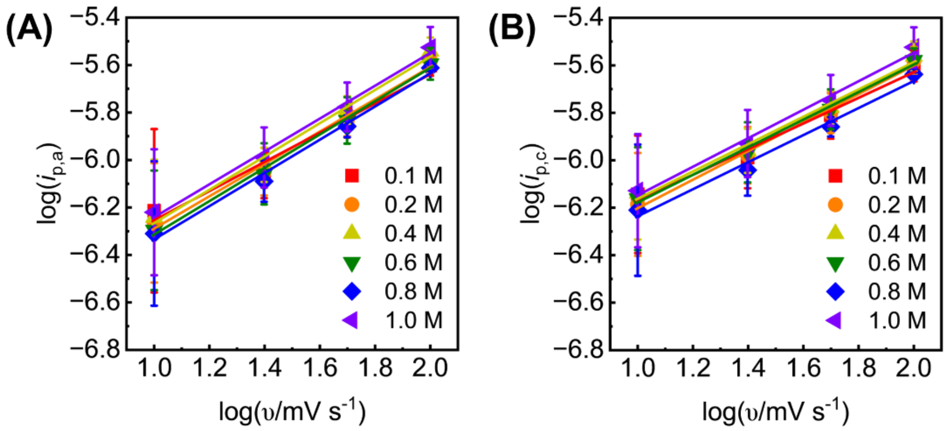

Figure S7. Plots showing **(A)** log($i_{p,a}$) or **(B)** log($i_{p,c}$) vs. scan rate in pH 8-adjusted MOPS buffer, where MOPS concentration was varied between 0.1 to 1 M.

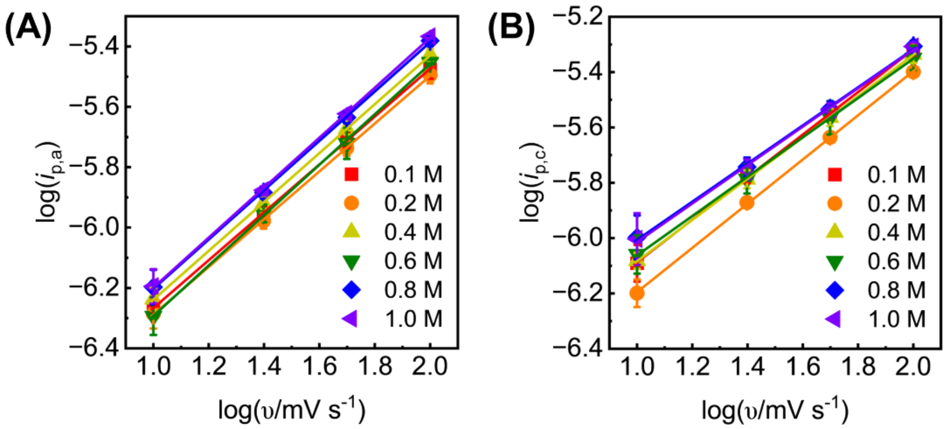

Figure S8. Plots showing **(A)** log($i_{p,a}$) or **(B)** log($i_{p,c}$) vs. scan rate in pH 7-adjusted Tris buffer, where Tris concentration was varied between 0.1 to 1 M.

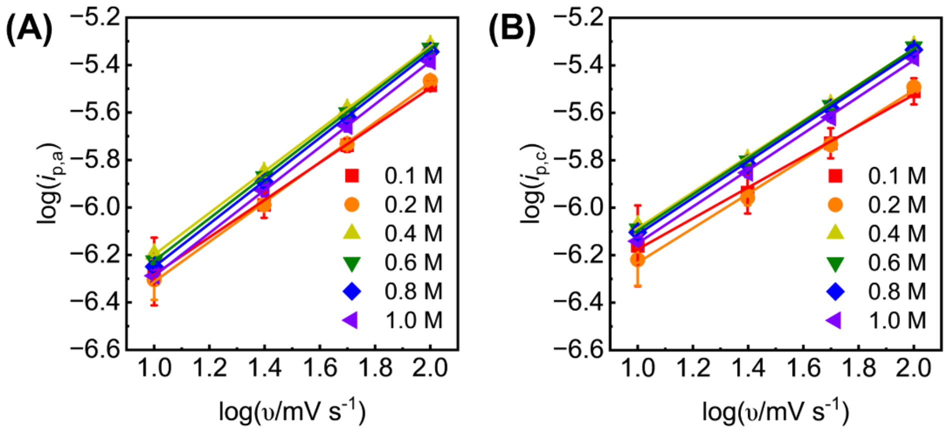

Figure S9. Plots showing **(A)** log($i_{p,a}$) or **(B)** log($i_{p,c}$) vs. scan rate in pH 8-adjusted Tris buffer, where Tris concentration was varied between 0.1 to 1 M.

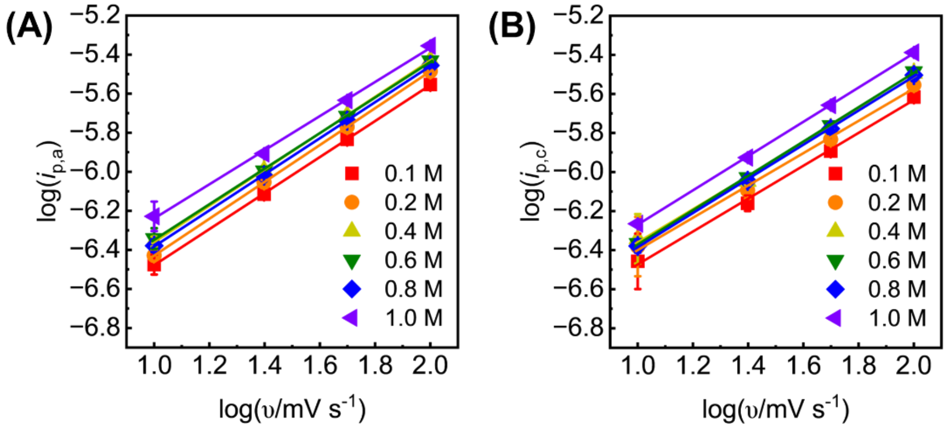

Figure S10. Plots showing **(A)** log($i_{p,a}$) or **(B)** log($i_{p,c}$) vs. scan rate in pH 9-adjusted Tris buffer, where Tris concentration was varied between 0.1 to 1 M.

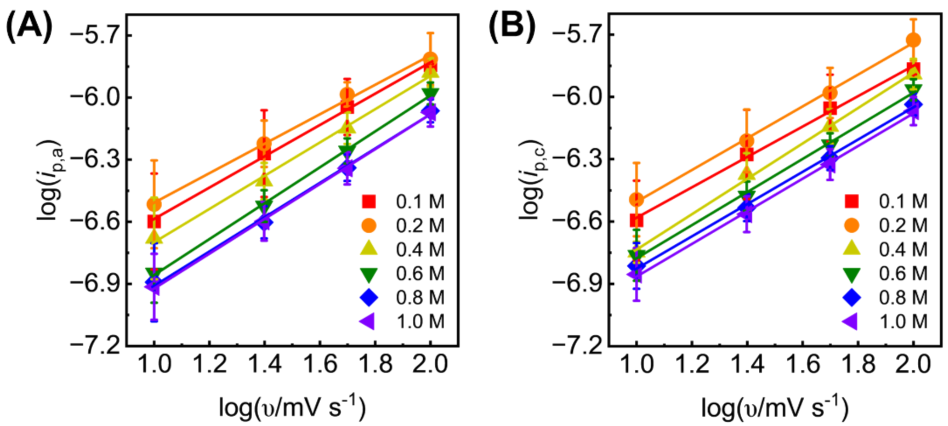

Figure S11. Plots showing **(A)** log($i_{p,a}$) or **(B)** log($i_{p,c}$) vs. scan rate in pH 8-adjusted borate buffer, where borate concentration was varied between 0.1 to 1 M.

Table S4. Slopes of log($i_{p,a}$) or log($i_{p,c}$) vs. log(υ) based on the CVs measured at various electrolytes.

Buffer	pH	Concentration (M)	Slopes vs. log(υ) for…	
			log($i_{p,a}$)	log($i_{p,c}$)
MOPS	8	0.1	0.75(5)	0.60(5)
		0.2	0.76(5)	0.65(4)
		0.4	0.77(4)	0.63(5)
		0.6	0.74(3)	0.62(3)
		0.8	0.79(3)	0.70(4)
		1.0	0.74(4)	0.64(4)
Tris	7	0.1	0.80(1)	0.76(1)
		0.2	0.80(1)	0.79(1)
		0.4	0.81(1)	0.75(1)
		0.6	0.84(1)	0.71(1)
		0.8	0.83(1)	0.71(2)
		1.0	0.85(1)	0.73(2)
	8	0.1	0.82(2)	0.68(3)
		0.2	0.88(1)	0.79(2)
		0.4	0.88(1)	0.76(2)
		0.6	0.89(1)	0.76(2)
		0.8	0.90(1)	0.77(2)
		1.0	0.90(1)	0.77(2)
	9	0.1	0.93(1)	0.91(1)
		0.2	0.94(1)	0.91(2)
		0.4	0.94(1)	0.94(1)
		0.6	0.93(1)	0.90(1)
		0.8	0.94(1)	0.90(1)
		1.0	0.91(1)	0.88(1)
H_3BO_3	8	0.1	0.72(2)	0.71(2)
		0.2	0.71(5)	0.78(2)
		0.4	0.84(3)	0.86(2)
		0.6	0.89(2)	0.82(2)
		0.8	0.87(3)	0.80(2)
		1.0	0.86(2)	0.80(2)

4.3 Details on CPE measurements

CPEs of Ce-MOF-808|FTOs were at ±240 mV with respect to the peak potentials of the $Ce^{4+}O/Ce^{3+}OH$ redox features. All CPE measurements were performed by applying a pre-electrolysis potential corresponding to the counter-reaction to ensure all redox-active nodes are either fully reduced or oxidized. Below are the chronoamperometric curves measured at various concentrations of buffers and/or pH.

4.4 Details on Cottrell Analysis of CPE Data

Apparent electron diffusion coefficients (D_{app}'s) were calculated using the Cottrell equation (eq. S1), as described in the main text. C_{Ce} is the effective concentration of Ce cations in the thin film, estimated from the film area (standardized to 0.25 cm^2) and thickness; see Figure S3 for the cross-sectional SEM image. F represents Faraday's constant (96485 C mol^{-1}).

$$j = \frac{FC_{Ce}\sqrt{D_{app}}}{\sqrt{\pi t}} \tag{S1}$$

Below are representative Cottrell plots using the results from the chronoamperometric curves resulting from CPE analysis (Figures S12-S19). The errors on D_{app}'s shown in these figures are 1σ of the linear regression. Current density within the 1-5 s of the CPE measurements was used to estimate all D_{app} values, which are summarized in Table S5. Figures S20-S22 summarize all D_{app} values plotted against the concentration of buffers. These figures show that D_{app} has complex dependence on the buffer concentration and pH, and the trend between $D_{app,Ox}$ vs. $D_{app,Red}$ can be different. Precise determination of the reason behind this trend is beyond the scope of this work. We note that Figure S14A is also reproduced in the main text as Figure 2B.

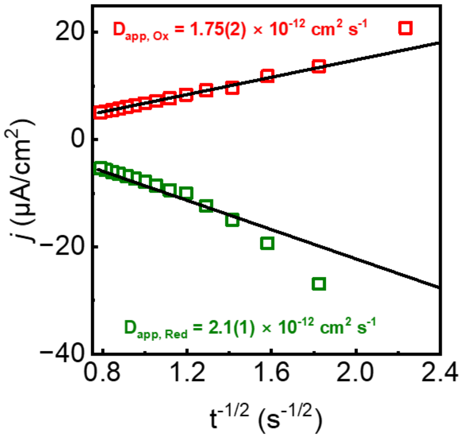

Figure S12. Representative Cottrell plot of Ce-MOF-808|FTO at $E_{CPE,a}$ and $E_{CPE,c}$, and the derived D_{app} values in pH 7-adjusted 0.1 M MOPS buffer.

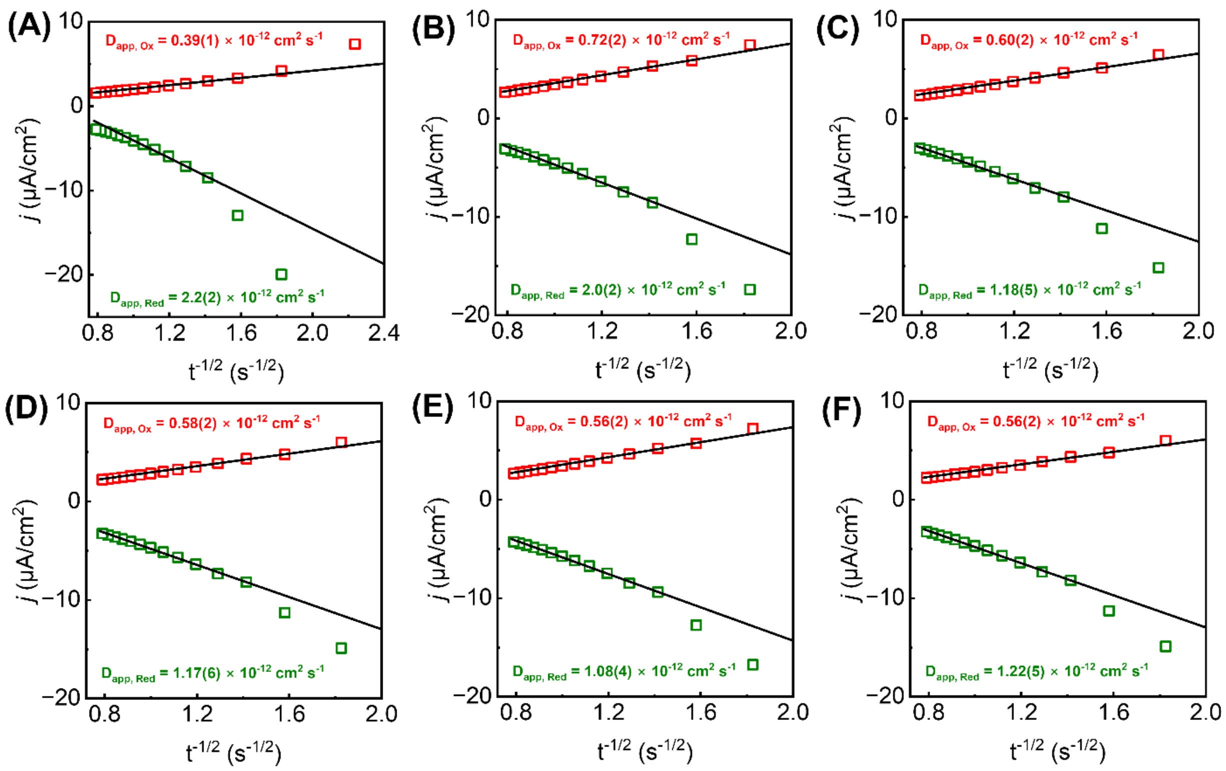

Figure S13. Representative Cottrell plots of Ce-MOF-808|FTO at $E_{CPE,a}$ and $E_{CPE,c}$, and the derived D_{app} values in pH 8-adjusted MOPS buffer. Concentrations of MOPS were adjusted at **(A)** 0.1, **(B)** 0.2, **(C)** 0.4, **(D)** 0.6, **(E)** 0.8, or **(F)** 1.0 M.

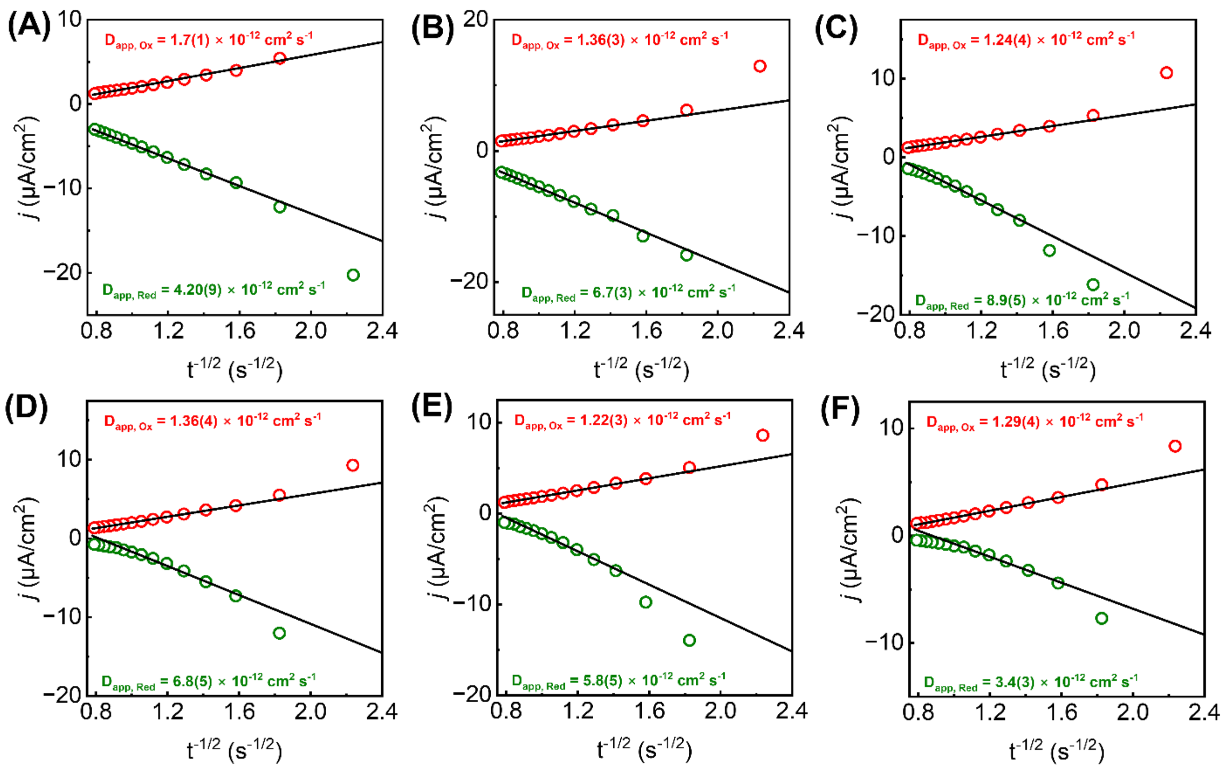

Figure S14. Representative Cottrell plots of Ce-MOF-808|FTO at $E_{CPE,a}$ and $E_{CPE,c}$, and the derived D_{app} values in pH 7-adjusted Tris buffer. Concentrations of Tris were adjusted at **(A)** 0.1, **(B)** 0.2, **(C)** 0.4, **(D)** 0.6, **(E)** 0.8, or **(F)** 1.0 M.

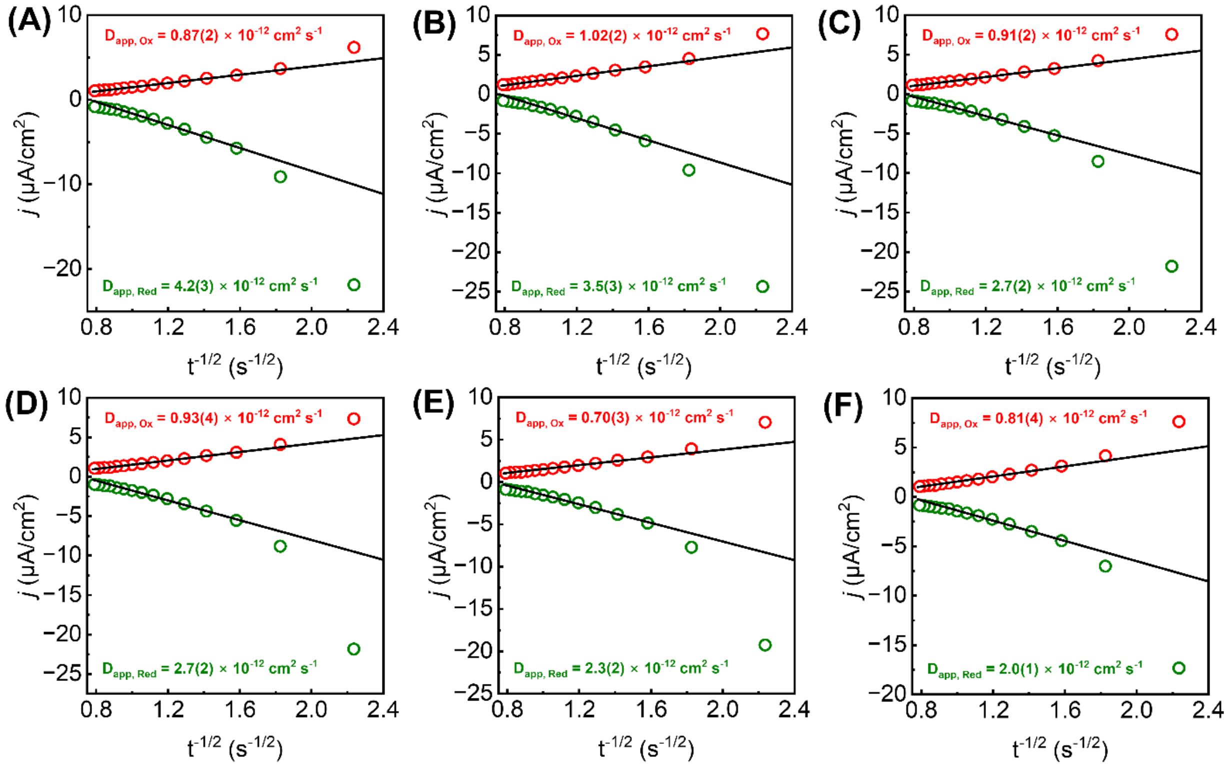

Figure S15. Representative Cottrell plots of Ce-MOF-808|FTO at $E_{CPE,a}$ and $E_{CPE,c}$, and the derived D_{app} values in pH 8-adjusted Tris buffer. Concentrations of Tris were adjusted at **(A)** 0.1, **(B)** 0.2, **(C)** 0.4, **(D)** 0.6, **(E)** 0.8, or **(F)** 1.0 M.

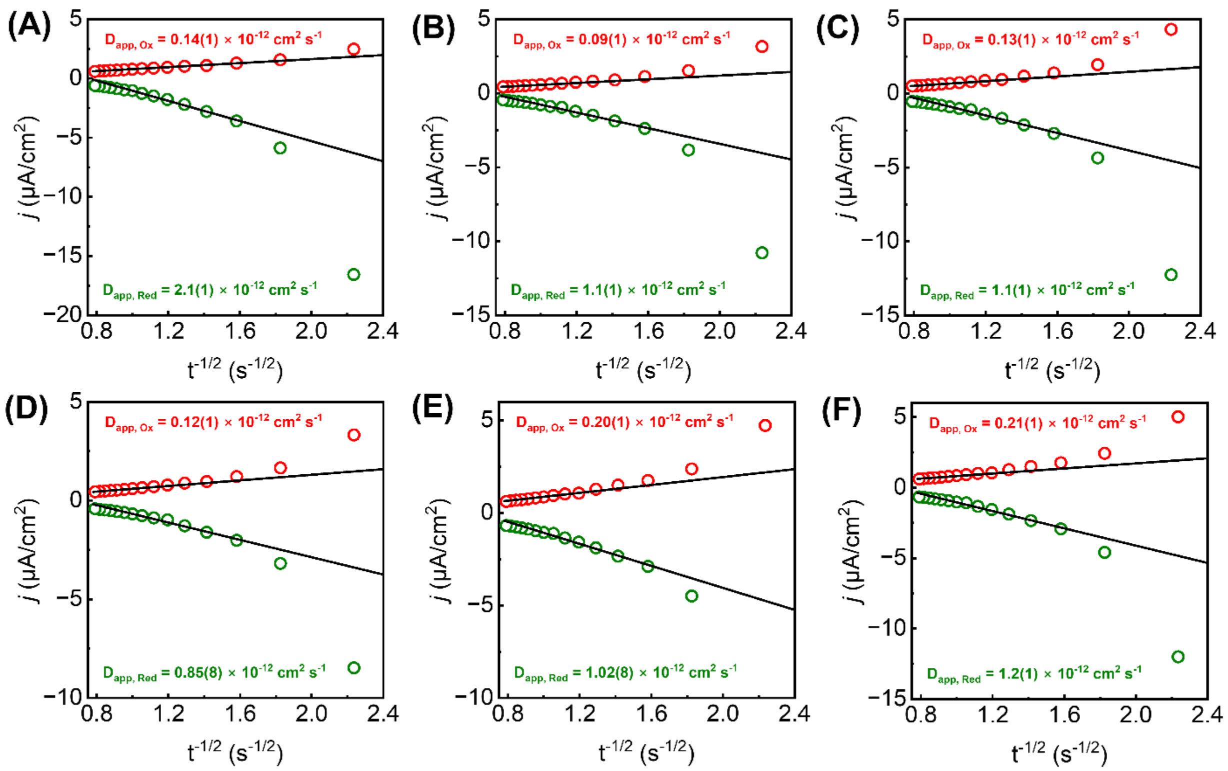

Figure S16. Representative Cottrell plots of Ce-MOF-808|FTO at $E_{CPE,a}$ and $E_{CPE,c}$, and the derived D_{app} values in pH 9-adjusted Tris buffer. Concentrations of Tris were adjusted at **(A)** 0.1, **(B)** 0.2, **(C)** 0.4, **(D)** 0.6, **(E)** 0.8, or **(F)** 1.0 M.

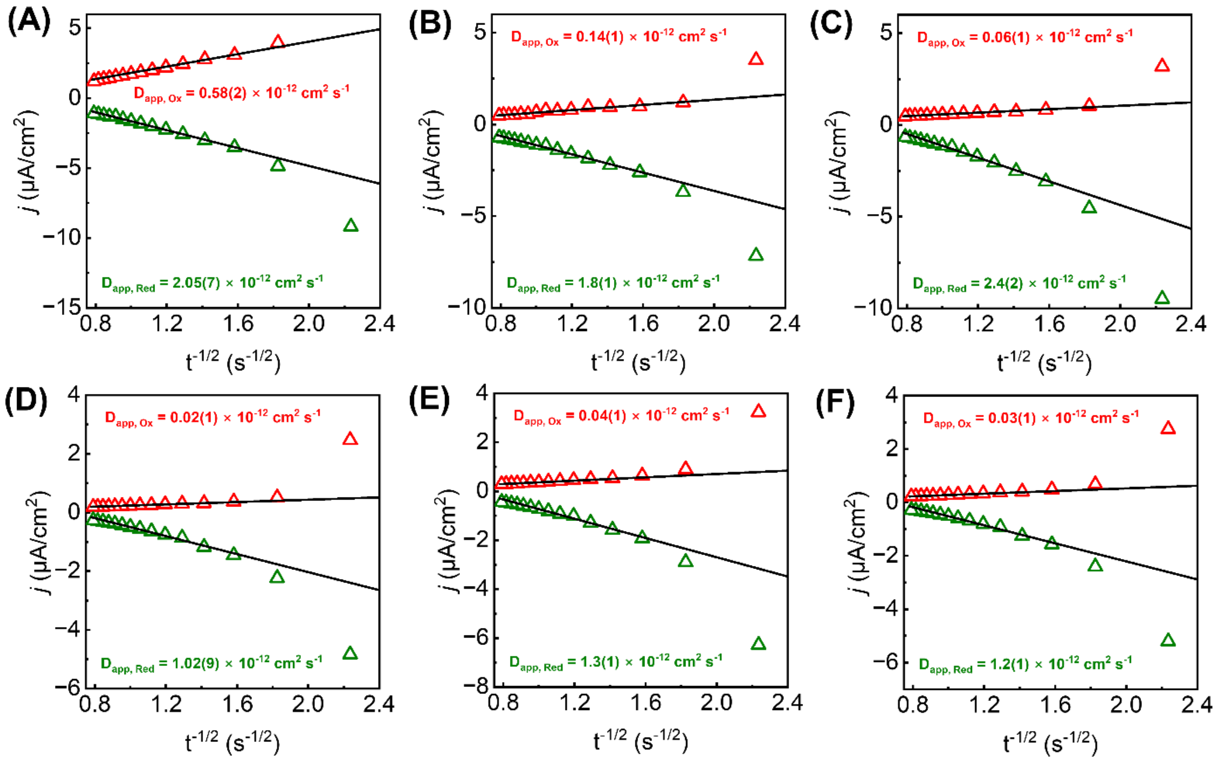

Figure S17. Representative Cottrell plots of Ce-MOF-808|FTO at $E_{CPE,a}$ and $E_{CPE,c}$, and the derived D_{app} values in pH 8-adjusted borate buffer. Concentrations of Tris were adjusted at **(A)** 0.1, **(B)** 0.2, **(C)** 0.4, **(D)** 0.6, **(E)** 0.8, or **(F)** 1.0 M.

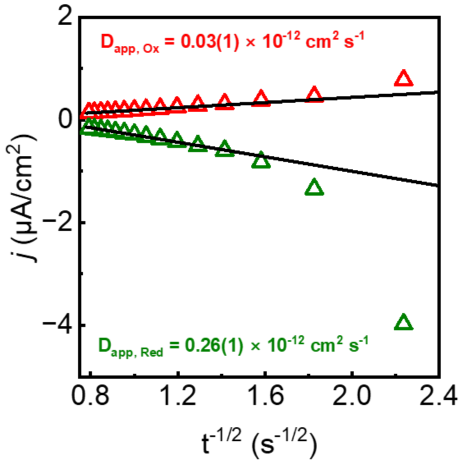

Figure S18. Representative Cottrell plot of Ce-MOF-808|FTO at $E_{CPE,a}$ and $E_{CPE,c}$, and the derived D_{app} values in pH 9-adjusted 0.1 M borate buffer.

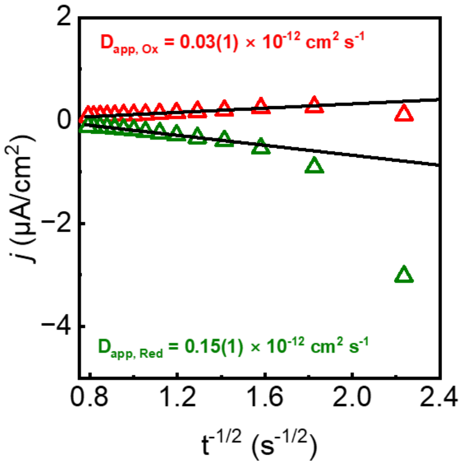

Figure S19. Representative Cottrell plot of Ce-MOF-808|FTO at $E_{CPE,a}$ and $E_{CPE,c}$, and the derived D_{app} values in pH 10-adjusted 0.1 M borate buffer.

Table S5. D_{app} values of Ce-MOF-808|FTO determined from Cottrell equations in electrolytes with distinct pHs, buffers, and concentrations.

Buffer	pH	Concentration (M)	$D_{app.Ox}$ (10^{-12} cm^2 s^{-1})	$D_{app.Red}$ (10^{-12} cm^2 s^{-1})
MOPS	7	0.1	1.1(2)	2.4(4)
	8	0.1	0.51(5)	3.2(5)
		0.2	0.58(9)	1.5(4)
		0.4	0.57(2)	0.9(5)
		0.6	0.54(3)	1.2(1)
		0.8	0.58(3)	1.2(1)
		1.0	0.7(1)	2.2(5)
Tris	7	0.1	1.5(2)	3.6(4)
		0.2	1.31(4)	5(1)
		0.4	1.3(1)	8(1)
		0.6	1.35(1)	6.3(8)
		0.8	1.24(2)	5.6(8)
		1.0	1.29(4)	3.7(4)
	8	0.1	0.89(4)	4.7(5)
		0.2	1.03(1)	3.4(2)
		0.4	0.97(5)	2.5(2)
		0.6	1.1(3)	2.65(9)
		0.8	0.8(1)	2.3(3)
		1.0	0.79(4)	1.92(7)
	9	0.1	0.13(1)	2.1(5)
		0.2	0.11(3)	1.3(3)
		0.4	0.11(2)	1.3(2)
		0.6	0.13(2)	0.9(1)
		0.8	0.17(2)	0.9(2)
		1.0	0.17(3)	1.2(7)
H_3BO_3	8	0.1	0.56(6)	1.8(3)
		0.2	0.14(7)	1.9(2)
		0.4	0.05(1)	2.3(5)
		0.6	0.02(1)	1.02(1)
		0.8	0.03(1)	1.25(8)
		1.0	0.03(1)	1.2(3)
	9	0.1	0.05(3)	0.5(2)
	10	0.1	0.04(2)	0.10(4)

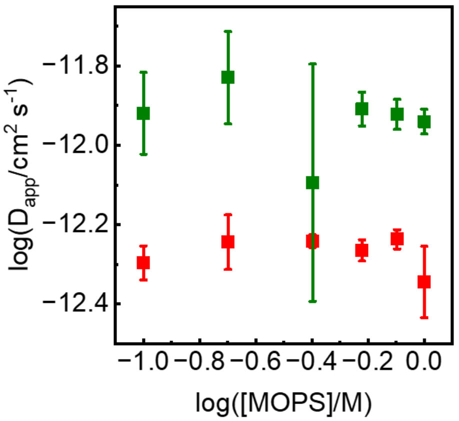

Figure S20. Plot showing log(D_{app}) vs. log([MOPS]). pH values of all electrolytes were adjusted to 8.

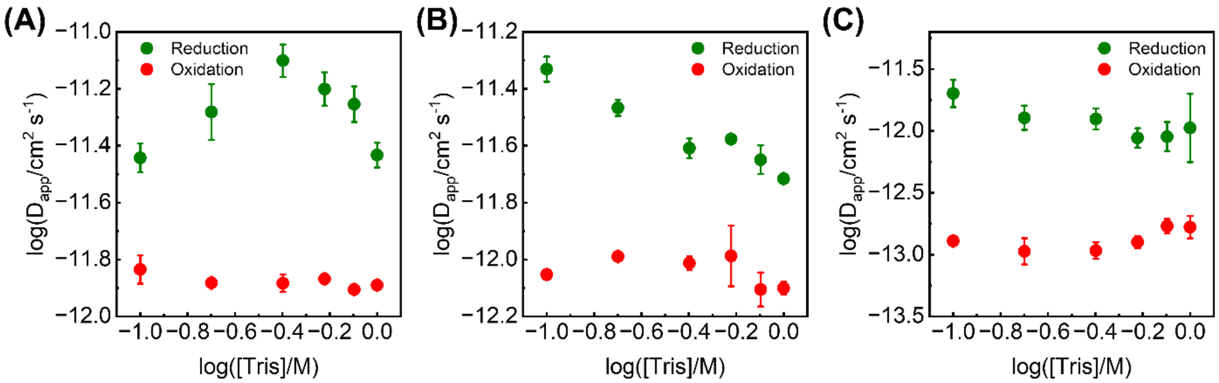

Figure S21. Plot showing log(D_{app}) vs. log([Tris]). pH values of electrolytes were adjusted to **(A)** 7, **(B)** 8, or **(C)** 9.

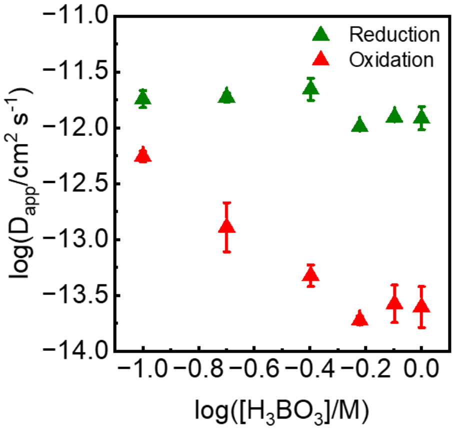

Figure S22. Plot showing log(D_{app}) vs. log([H_3BO_3]). pH values of all electrolytes were adjusted to 8.

4.5 *Investigation of Redox Anisotropy between $D_{app,Ox}$ and $D_{app,Red}$*

$D_{app,Red}$ of Ce-MOF-808|FTO was consistently larger than $D_{app,Ox}$ by at least 4 times in all electrolytes. As described in the main text and above, for all D_{app} measurements, $E_{CPE,a}$ was applied first to determine $D_{app,Ox}$. Subsequently, $E_{CPE,c}$ was applied to determine $D_{app,Red}$. While pre-electrolysis at $E_{CPE,a}$ or $E_{CPE,c}$ were performed at all measurements to ensure that all redox-active sites are reduced or oxidized, this order of D_{app} measurements was altered at pH 8-adjusted 0.1 M MOPS buffer to ensure that this is not causing the apparent anisotropy in D_{app} values. As shown below, while $D_{app,Red}$ indeed decrease, it is still larger by more than 4 times compared to $D_{app,Ox}$. Thus, the observed anisotropy is independent of the experimental protocol; see Figure S23.

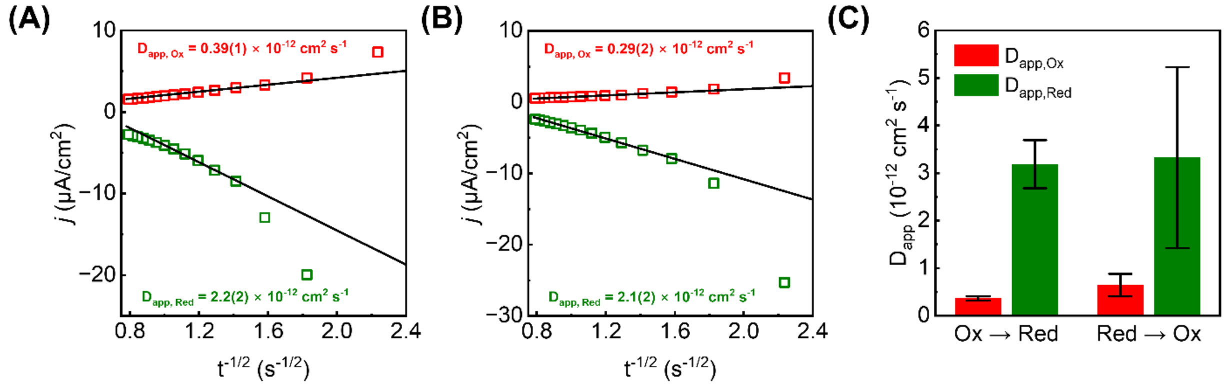

Figure S23. Representative Cottrell plots of Ce-MOF-808|FTO at $E_{CPE,a}$ and $E_{CPE,c}$ by applying **(A)** $E_{CPE,a}$ first then $E_{CPE,c}$, or **(B)** $E_{CPE,c}$ first then $E_{CPE,a}$. **(C)** Average D_{app} values measured from the Cottrell plots.

4.6 *Attempts to Measure Intrinsic Electron Transfer Rate Constants*

The rate constants described in the main text, derived from the D_{app} values, are related to the *hopping* kinetics of H^+/e^- within the MOF lattice. To measure the intrinsic rate constant of the electron transfer reaction (k_{ET}) that initiates the PCET hopping reaction, we have attempted to use the Nicholson method and electrochemical impedance spectroscopy (EIS). As described below, both attempts were unsuccessful in determining k_{ET} values intrinsic to Ce-MOF-808.

First, we have attempted to measure k_{ET} values using the Nicholson method.7,8 CVs of Ce-MOF-808|FTO were measured at υ ranging between 0.01 to 10 V s^{-1} in pH 8-adjusted 0.1 M Tris buffer (Figure S24A). This method assumes that a dimensionless kinetic parameter (Ψ) linearly correlates with $\upsilon^{-1/2}$; see eq. S2. In eq. S2, D in the denominator can refer to $D_{app,Ox}$ or $D_{app,Red}$. Ψ is correlated to the CV-derived peak-to-peak separations (ΔE_p's) according to eq. S3. For both equations, other parameters refer to their usual definition. The slope is correlated with k_{ET}.

$$\Psi = \frac{\left(\frac{D_{app,Ox}}{D_{app,Red}}\right)^{\frac{\alpha}{2}} k_{ET}}{\left(\frac{n\pi D F \upsilon}{RT}\right)^{\frac{1}{2}}} \tag{S2}$$

$$\Psi = 2.18\left(\frac{\alpha}{\pi}\right)^{\frac{1}{2}} \exp\left(-\frac{\alpha^2 nF}{RT}\Delta E_p\right) \tag{S3}$$

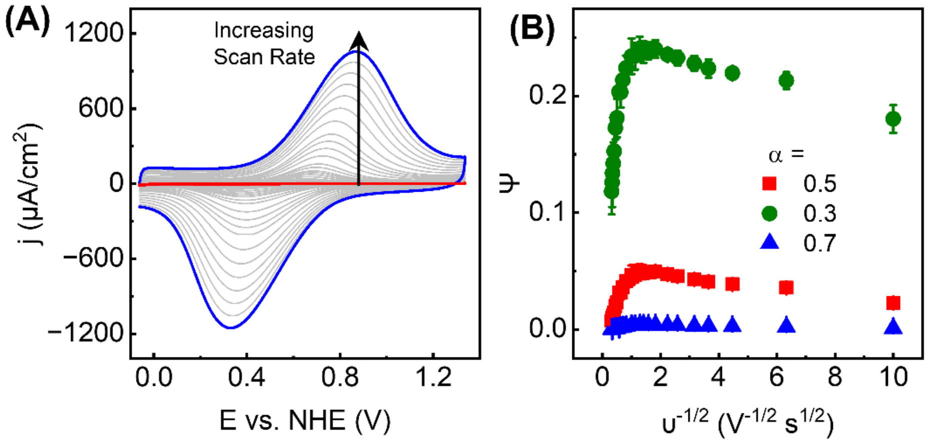

Figure S24. Representative CVs of Ce-MOF-808|FTO in scan rates from 0.01 to 10 V s^{-1}. **(B)** Plots of CV-derived Ψ vs. $υ^{-1/2}$ plots with α = 0.3, 0.5, or 0.7.

As shown in Figure S24B, Ψ values did not scale with $υ^{-½}$ in a linear fashion. This may be due to two reasons. First, eq. S2 assumes that $D_{app,Ox} \approx D_{app,Red}$, which we have shown in Figures 2C and 2D in the main text, that this assumption is not valid for Ce-MOF-808. We have also described in the main text that α in eq. S3 may range between 0.3 to 0.7. Even in these cases, Ψ values did not linearly scale with $υ^{-½}$. We suspect that the chemical reaction of buffer binding to the node after electron transfer is the reason behind this anomaly.

Next, we attempted to use EIS to measure k_{ET} values, which are inversely proportional to the charge-transfer resistance (R_{CT}; eq. S4).[9] EIS measurements were collected using four freshly prepared in an identical electrolyte to that used above. All electrodes were held at the excitation potential of 0.5 V vs. NHE with the frequency range and AC amplitude of 0.03 to 100000 Hz and 10 mV, respectively.

The parameters, A and C, in eq. S4 refer to the area and concentration of Ce. C was determined from the average of charges passed from CPEs. Figure S25 and Table S6 show that both the R_{CT} and double-layer capacitance (C_{dl}) derived from the fits of the Nyquist plot, using the Randles circuit reported in the following reference. In Table S6, R_s refers to the series resistance.

$$R_{CT} = \frac{RT}{n^2F^2ACk_{ET}} \tag{S4}$$

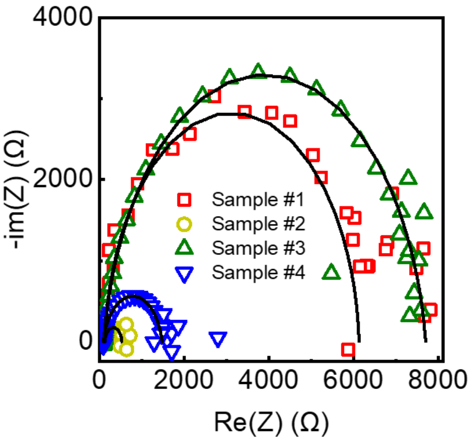

Figure S25. EIS-derived Nyquist plots of three Ce-MOF-808|FTO.

Table S6. R_s, R_{CT}, and C_{dl} from fits of Nyquist plots using the Randles circuit.*

Sample Number	R_s (Ω)	R_{CT} (kΩ)	k_{ET} (10^{-7} cm s^{-1})	C_{dl} (μF)
1	121(3)	6.0(3)	7.2(4)	2.8(3)
2	121(3)	0.40(1)	107(4)	3.1(8)
3	97(3)	7.6(6)	5.7(5)	3.9(4)
4	86(3)	1.4(1)	31(2)	10(2)

*All standard errors are 1σ of fits using a standard Randles circuit.

To further demonstrate that R_{CT} and C_{dl} are correlated with the amount of Ce-MOF-808 on FTO, we have employed ^{1}H NMR of Ce-MOF-808|FTO digested in ~1 M NaOD/D_2O using our reported procedure.[10,11] Figure S26A is a representative ^{1}H NMR spectrum. The amount of MOF per unit geometric area of the FTO electrode (Γ in nmol cm^{-2}) is plotted against R_{CT} or C_{dl} shown in Table 6; see Figures S26B and S26C. Clearly, C_{dl} linearly scaled with Γ, suggesting that the electroactive surface area is largely dictated by the amount of Ce-MOF-808 close to the underlying FTO surface. In contrast, R_{CT} seemingly exhibited a 'volcano-shaped' trend with respect to Γ. This is likely due to an increase in electrical resistance with excessive Ce-MOF-808.

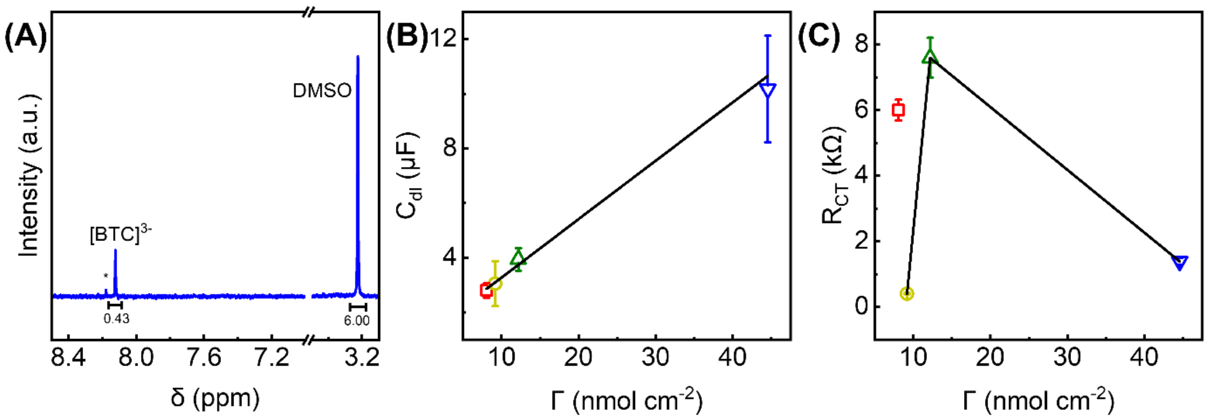

Figure S26. (A) Representative ^{1}H NMR spectrum of Ce-MOF-808 digested in *ca.* 1 M NaOD/D_2O. The peak denoted with * is likely due to the presence of formate. Plots of ^{1}H NMR-derived Γ vs. **(B)** C_{dl} or **(C)** R_{CT}. Error bars on **(B)** and **(C)** represent 1σ of fits from Table S6.

From the above, we concluded that there is a large sample-to-sample variation in R_{CT} and C_{dl}. This is likely due to the difference in surface coverages of MOFs. C_{dl}, for example, is sensitive to electrochemically active Ce cations. Consequently, the R_{CT} ranged between 400 to >7500 Ω. In contrast, D_{app} values had smaller sample-to-sample variation because these values refer to PCET hopping after the initial charge transfer. Thus, we prefer not to focus on k_{ET} values for this study.

5 Investigation of Ce-MOF-808–Buffer Interactions

5.1 Details on Isothermal Titration Calorimetry

Isothermal titration calorimetry (ITC) of Ce-MOF-808 was performed following the modified procedure reported previously. Briefly, 4.8 mg of Ce-MOF-808 suspension in 3.05 mL of 0.1 M NaCl solution and buffer solutions were both prepared on the same day of analysis. The pH values of both solutions were adjusted to the desired value. Buffer concentrations varied between 8-50 mM, which depended on the limit of detection of the calorimeter and the saturation point of Ce-MOF-808—observed as a plateau in the enthalpy signals following each buffer injection; see Figure S26-S30. A 'background' titration between the buffer solution and 0.1 M NaCl without the MOF was subtracted from those with the MOF to determine thermodynamic parameters that represent buffer-MOF interactions. The exact reaction conditions are summarized in Table S7.

As shown in Figures S29-S30, injection of pH 9 or 10-adjusted borate solution into 0.1 M NaCl solution without the MOF exhibited significant endothermic features. Speculations on this endothermic, but entropically driven, borate-node interactions can be found in the main text.

Table S7. Working solutions and measurement conditions used in Isothermal Titration Calorimetry.

Buffer	Buffer concentration (mM)	Ce-MOF-808 concentration (mM)	Injection volume (µL)	Matrix pH (0.1 M NaCl)
MOPS	10	1.0	1.5	8
Tris	25	1.0	1.5	8
Tris	8.0	1.0	1.5	9
H_3BO_3	50	1.0	1.5	9
H_3BO_3	25	1.0	1.5	10

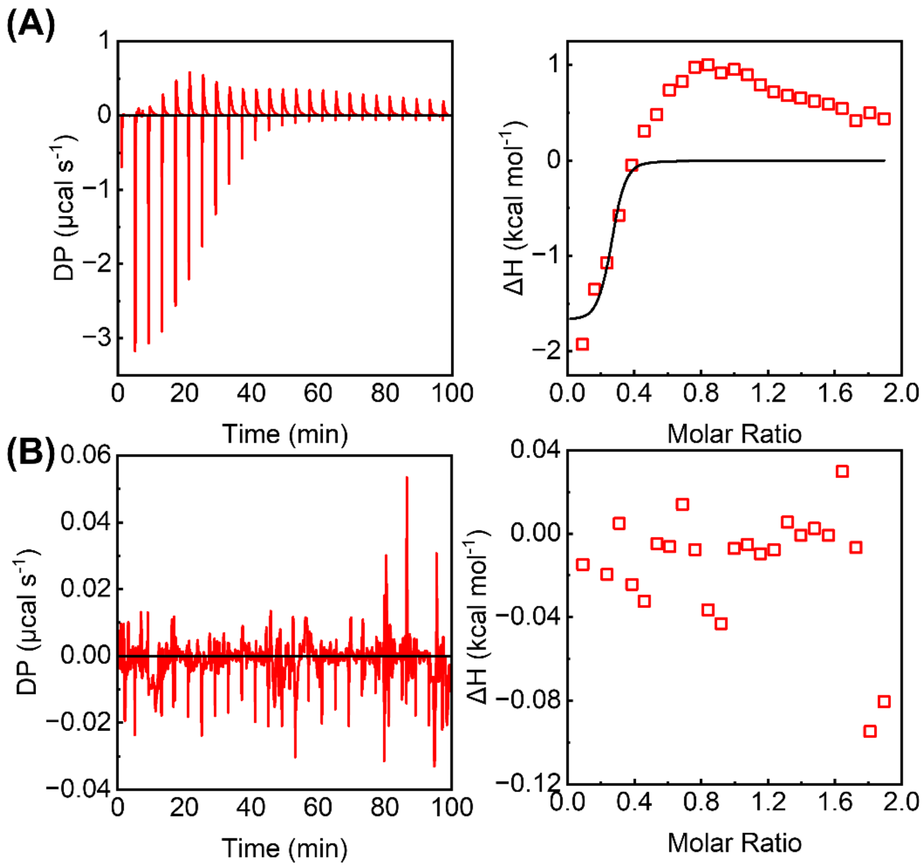

Figure S26. Representative ITC thermograms for the titrations of **(A)** Ce-MOF-808 suspensions or **(B)** blank saline solution using pH 8-adjusted 10 mM MOPS.

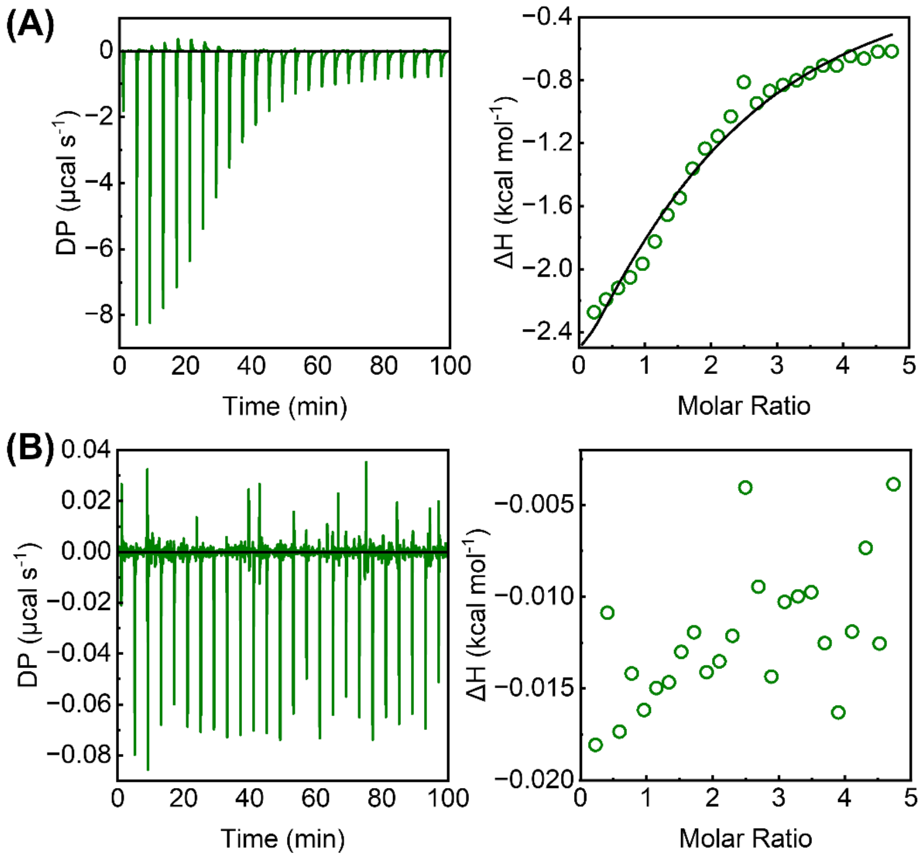

Figure S27. Representative ITC thermograms for the titrations of **(A)** Ce-MOF-808 suspensions or **(B)** blank saline solution using pH 8-adjusted 25 mM Tris.

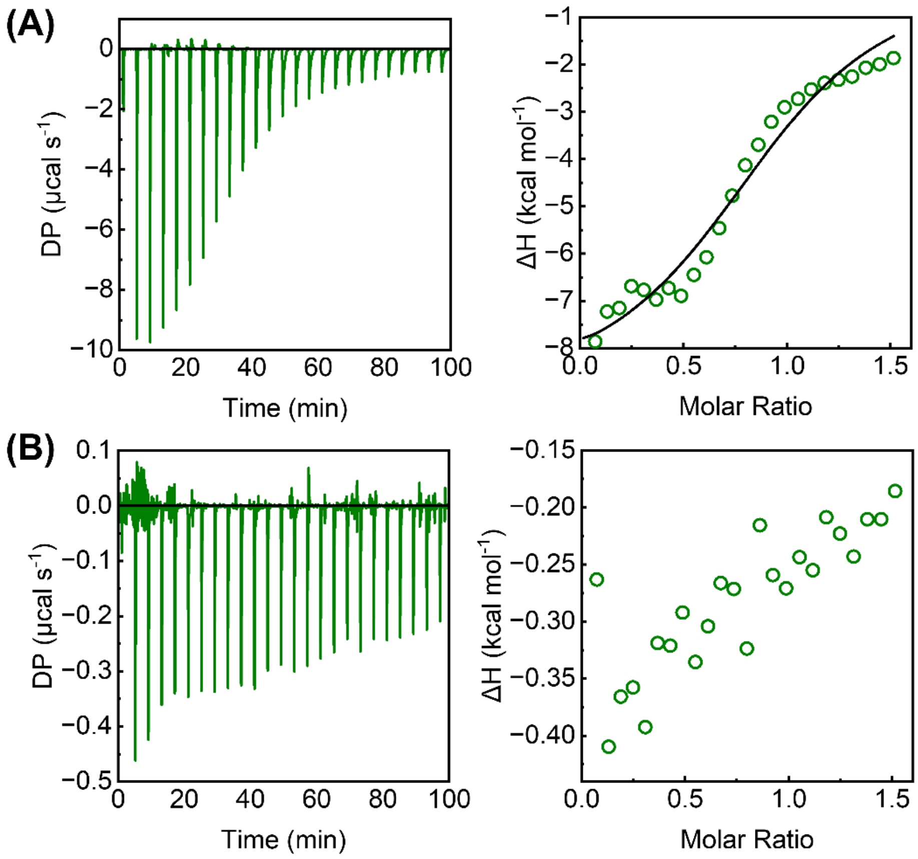

Figure S28. Representative ITC thermograms for the titrations of **(A)** Ce-MOF-808 suspensions or **(B)** blank saline solution using pH 9-adjusted 8.0 mM Tris.

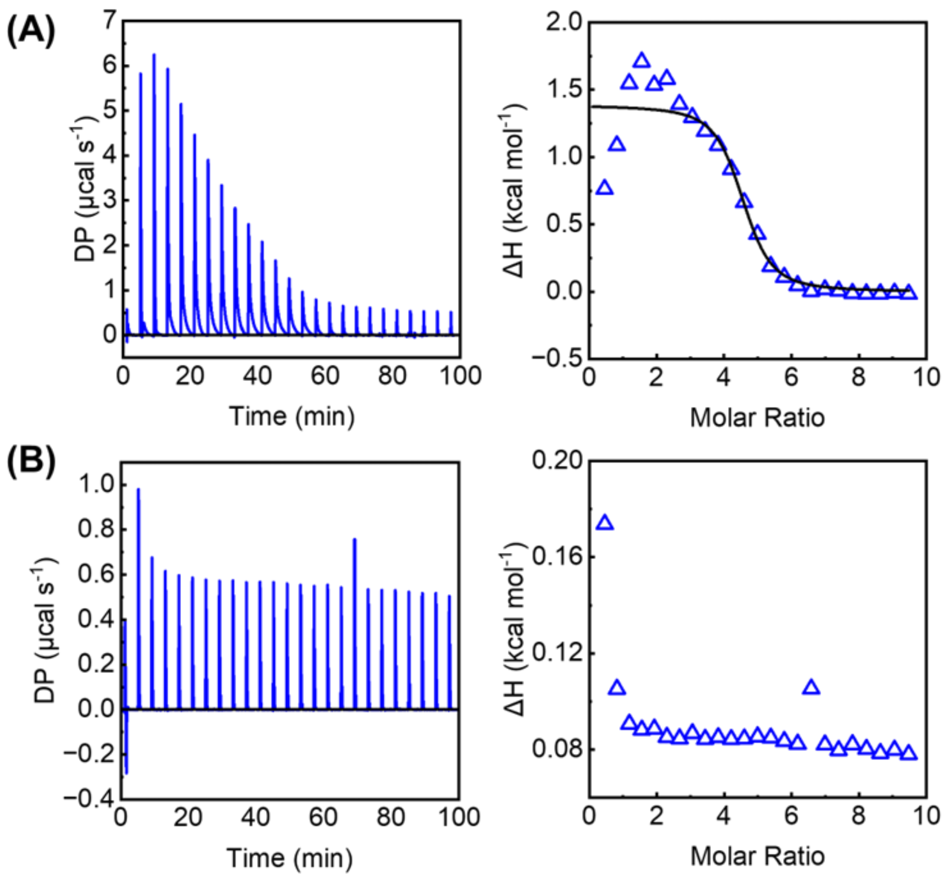

Figure S29. Representative ITC thermograms for the titrations of **(A)** Ce-MOF-808 suspensions or **(B)** blank saline solution using pH 9-adjusted 50 mM boric acid.

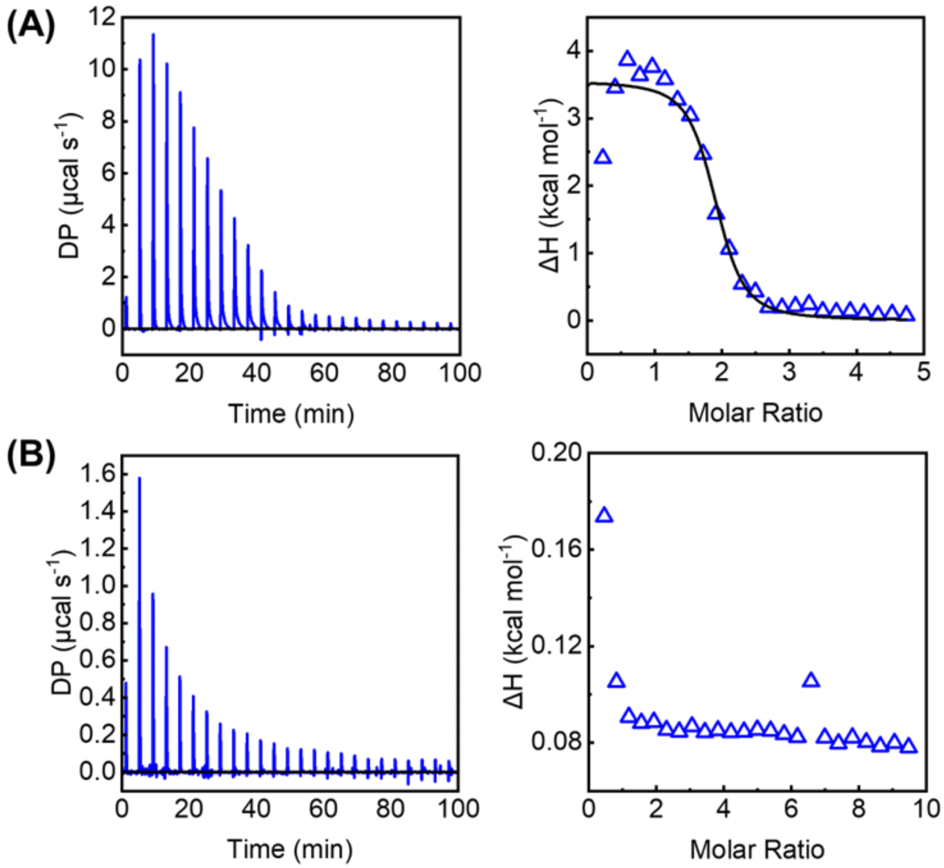

Figure S30. Representative ITC thermograms for the titrations of **(A)** Ce-MOF-808 suspensions or **(B)** blank saline solution using pH 10-adjusted 25 mM boric acid.

6 Computational Details

6.1 Details on Computational Methods

All calculations were performed using ORCA 6.0 software.[12] A truncated cluster model of Ce-MOF-808 was employed, following our previous work.[3] In this model, the benzene-1,3,5-tricarboxylate linkers were replaced by formate groups to yield the composition $Ce_6(H_2O)_6(OH)_6(\mu_3\text{-}OH)_4(\mu_3\text{-}O)_4(\text{formate})_6$. No structural constraints were applied during geometry optimizations.

Assessment of multiconfigurational character. Fractional Occupation Density (FOD) analysis[13] was performed with ωB97X/def2-SVPD[14,15] and the SMD[16] implicit solvation model for water at a smearing temperature of 10500 K. The value obtained was N_{FOD} = 0.695 |e|, with fractional density localized mainly on Ce atoms. Additional tests with UKS and RKS methods showed no instabilities. Unrestricted KS calculations converged to restricted solutions, and κ-MP2/UHF also collapsed to RHF. Together with the fact that Ce^{4+} is formally f^0, these results indicate no significant multireference character, and therefore DFT is an appropriate method for describing this system.

Level of theory. To test how adequate is the chosen level of theory we compared bond dissociation free energies (BDFEs) obtained at the ωB97M-V/def2-TZVPPD/SMD(water)//ωB97X-D4/def2-SVPD/SMD(water) level with the experimental results. As shown in Table S8, results obtained with our method of choice are consistent with both prior computations and experimental thermochemistry.[3] All further reported calculations were therefore performed at this composite level of theory. For reduced clusters, the doublet spin-state was assumed. We found that spin densities were localized predominantly on a single Ce atom adjacent to a protonation site, yielding a formal $(Ce^{3+}(OH))Ce^{4+}{}_5O_{19}H_{22}$ cluster. Harmonic frequency calculations were performed for all optimized structures, and no imaginary modes were found, confirming them as local minima.

Atom colors of all Ce_6 clusters are identical here onwards: Ce^{4+} = light yellow, O = red, C = brown, H = white, N = blue, B = pink, S = yellow. All table entries here onwards refer to the labels on the corresponding figures. XYZ coordinates are provided in *Section 6.4*.

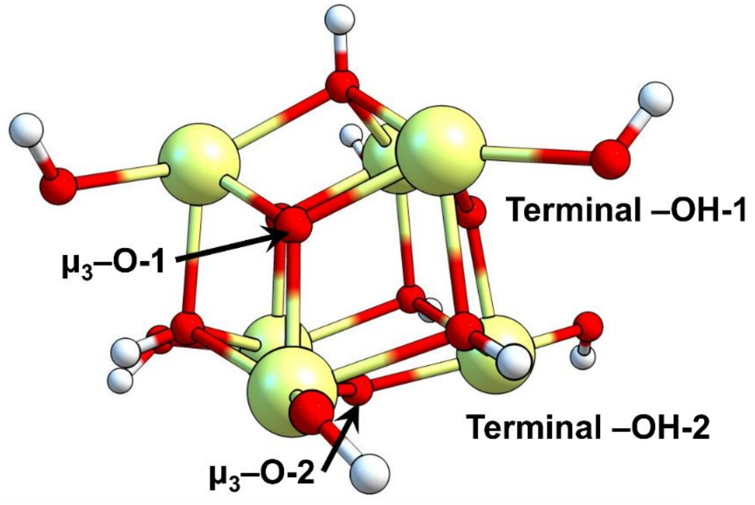

Figure S31. Optimized structure of Ce-MOF-808 cluster model with different protonation sites highlighted. Formate and water ligands are not shown for clarity.

Table S8. Computed and experimental BDFEs and structural changes for different protonation sites in the Ce-MOF-808 cluster model.

Protonation site	BDFE (kcal mol^{-1})	Ce−O(H) distance change[a] (Å)
Terminal –OH-1	85.76	0.432
Terminal –OH-2	86.30	0.427
μ_3–O-1	75.96	0.191
μ_3–O-2	73.23	0.180
Experimental[b]	78 ± 2	–

[a]For proton topologies μ_3-O-1 and μ_3-O-2, the average Ce–O(H) distances between the three neighboring Ce atoms were calculated [b]Experimental BDFE value from Ref. [3] is listed.

6.2 Proton Transfer between Buffer and Ce_6 Nodes

To evaluate the acidity of the Ce_6 node, we estimated the Gibbs free energy change for the reaction:

$$Ce_6O_{20}H_{22} = Ce_6O_{20}H_{21}^- + H^+$$

The pK_a values were then computed using the relation $\Delta G = -RT * \ln(K_a)$. It is well known that assigning an absolute ΔG for a solvated proton computationally is challenging.[17] We therefore followed the approach of Ref. 12, adopting a value of −270 kcal mol^{-1} for $\Delta G_{solv}(H^+)$. Several possible Brønsted-acidic sites of the Ce_6 cluster were considered. Their numbering is shown in Figure S30. As summarized in Table S9, the most acidic protons are the μ_3-OH groups, with the corresponding pK_a values of 7-9. Additionally, we also estimated the acid-base free energies (ΔG_{ab}) for the reactions of the type:

$$Ce_6O_{20}H_{22} + A^- = Ce_6O_{20}H_{19}^- + HA$$

where A^- and HA represent the deprotonated and protonated buffer molecules, respectively. For Tris, A^- corresponds to neutral Tris and HA to protonated $[TrisH]^+$. Six non-equivalent deprotonation sites (terminal –OH/OH_2 and μ_3–OH positions) were tested with the numbering shown in Figure S32. Corresponding reaction free energies are summarized in Table S9, and the resulting optimized structures of the deprotonated node are shown in Figure S33. Notably, only for $[H_2BO_3]^-$ the proton abstraction from the Ce_6 cluster is thermodynamically favorable, whereas Tris and MOPS do not show such behavior.

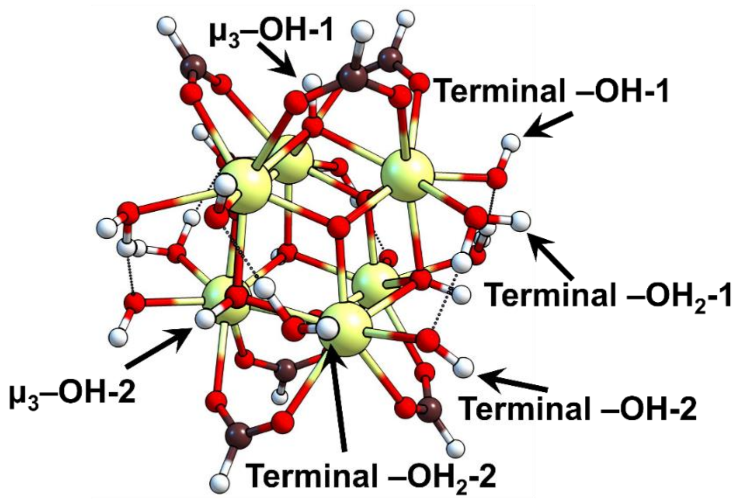

Figure S32. Optimized structure of Ce-MOF-808 cluster model with different deprotonation sites highlighted.

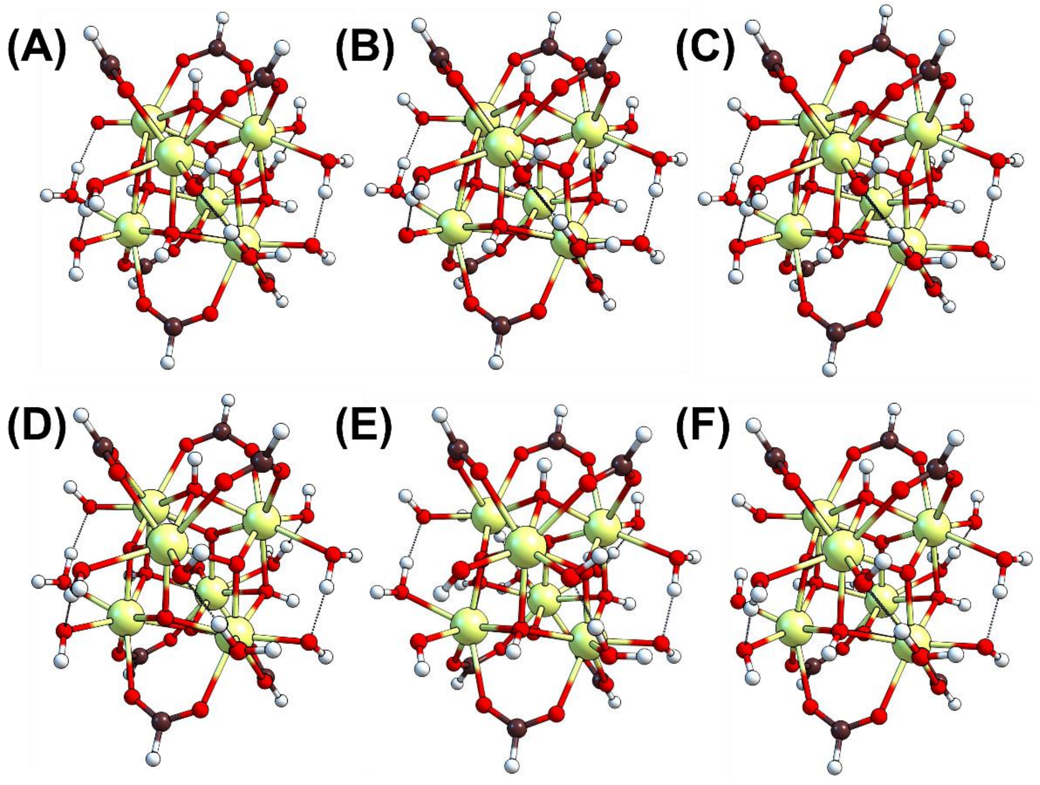

Figure S33. Optimized structures of deprotonated Ce_6 nodes.

Table S9. Computed pK_a values and acid-base reaction free energies (ΔG_{ab}) for different protonation sites of the Ce-MOF-808 cluster model with various buffers.

Table Entry	Deprotonation Site	pK_a	ΔG_{ab} (kcal mol^{-1})		
			Borate	Tris	MOPS
A	Terminal –OH-1	11.3	–5.66	9.72	6.70
B	Terminal –OH-2	10.7	–6.47	8.91	5.89
C	μ_3–OH-1	7.2	–11.19	4.18	1.17
D	μ_3–OH-2	8.9	–8.86	6.51	3.50
E	Terminal –OH_2-1	10.5	–6.65	8.72	5.71
F	Terminal –OH_2-2	10.4	–6.91	8.46	5.44

The above computational model does not treat the H_2O molecules in the solvent explicitly. A fully explicit treatment of the pore environment would, in principle, require a simulation of at least one MOF unit cell that is composed of ~1400 atoms. This remains a challenge in the field and is well beyond the scope of this work. Nevertheless, we attempted to introduce one water molecule at the close proximity of the node to examine its effect on pK_a and ΔG_{ab}. The results are summarized in Table S10. The optimized structures are shown in Figure S34.

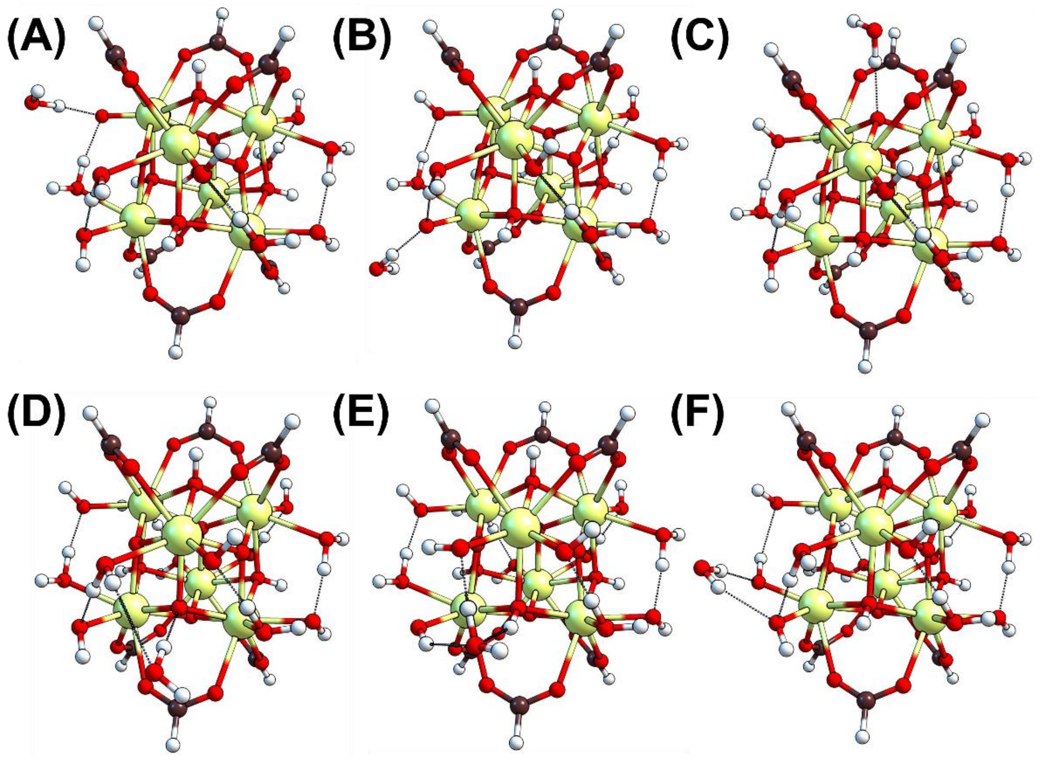

Figure S34. Optimized structures of deprotonated Ce_6 nodes with one H_2O molecule as an explicit solvent molecule.

Table S10. Computed pK_a values and acid-base reaction free energies (ΔG_{ab}) with various buffers for different protonation sites of the Ce-MOF-808 cluster model with one explicit water molecule coordinated.

Table Entry	Deprotonation Site	pK_a	ΔG_{ab} (kcal mol^{-1})		
			Borate	Tris	MOPS
A	Terminal –OH-1	9.2	–8.45	3.91	6.92
B	Terminal –OH-2	9.8	–7.65	4.71	7.72
C	μ_3–OH-1	7.6	–10.69	1.67	4.68
D	μ_3–OH-2	7.8	–10.39	1.97	4.99
E	Terminal –OH_2-1	8.9	–8.84	3.52	6.53
F	Terminal –OH_2-2	10.5	–6.68	5.68	8.69

6.3 Coordination of Buffer to Ce_6 Nodes

To investigate direct buffer-node coordination, substitution reactions were considered in which a buffer donor replaces a terminal –OH_2 or –OH ligand on the Ce_6 cluster. All associated free energies of insertion (ΔG_{Buf}) are listed in Table S11, and the optimized geometries are shown in

Figures S35-S37; for each buffer, structures that are thermodynamically most favorable are reproduced as Figures 4A-C in the main text. The ΔG_{Buf} of reduced species for the most energetically favorable insertions for Borate and Tris were generated by protonating the terminal –OH site of the Ce atom to which the buffer molecule was inserted, thereby reducing that Ce^{4+} center to Ce^{3+}. The resulting ΔG_{Buf} are 3.21 and 1.97 kcal mol^{-1} for borate and Tris, respectively. Figures 4D and E in the main text represent the geometry-optimized structure of these reduced nodes.

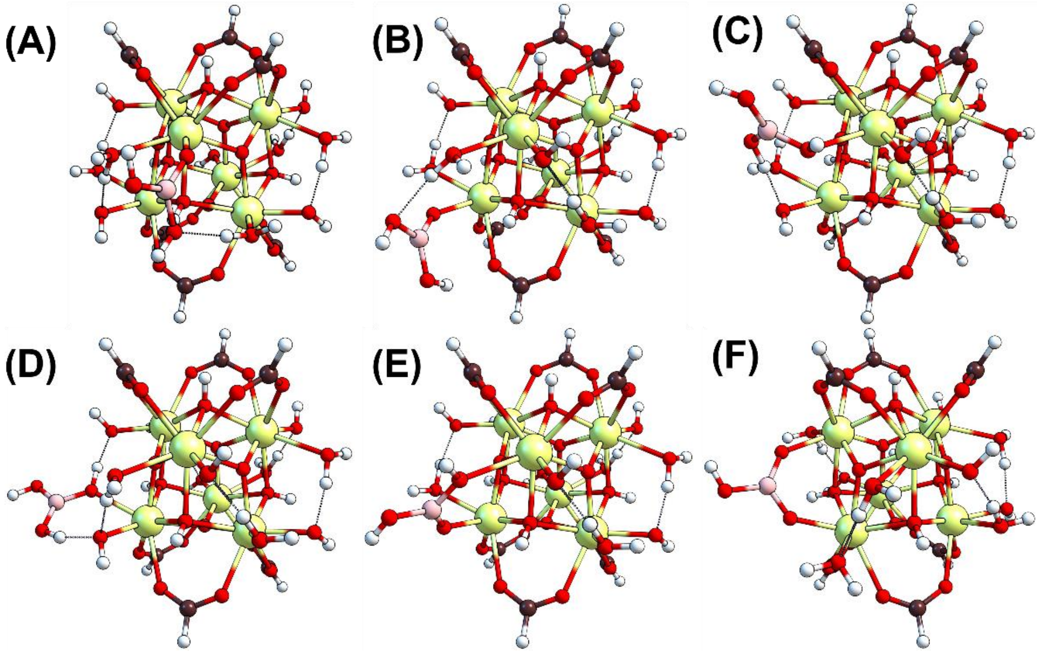

Figure S35. Optimized structures of oxidized Ce_6 nodes with node-bound borate.

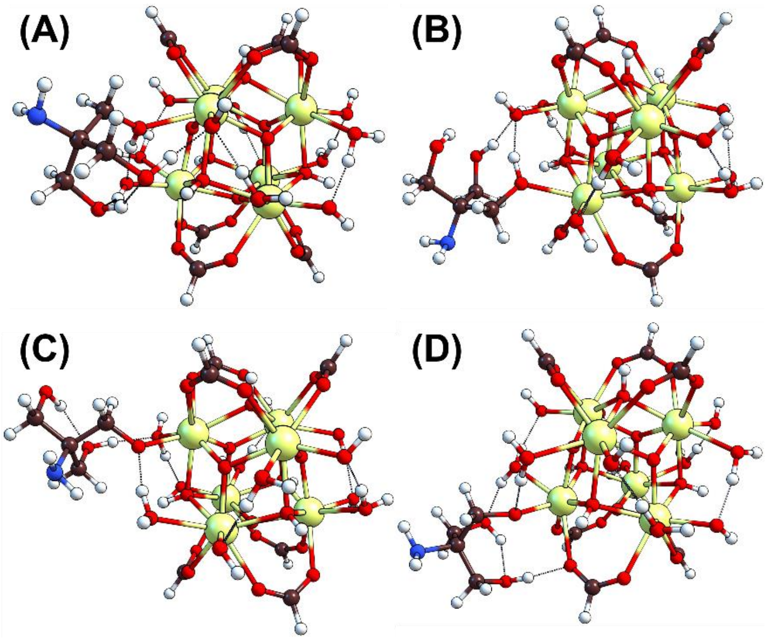

Figure S36. Optimized structures of oxidized Ce_6 nodes with node-bound Tris.

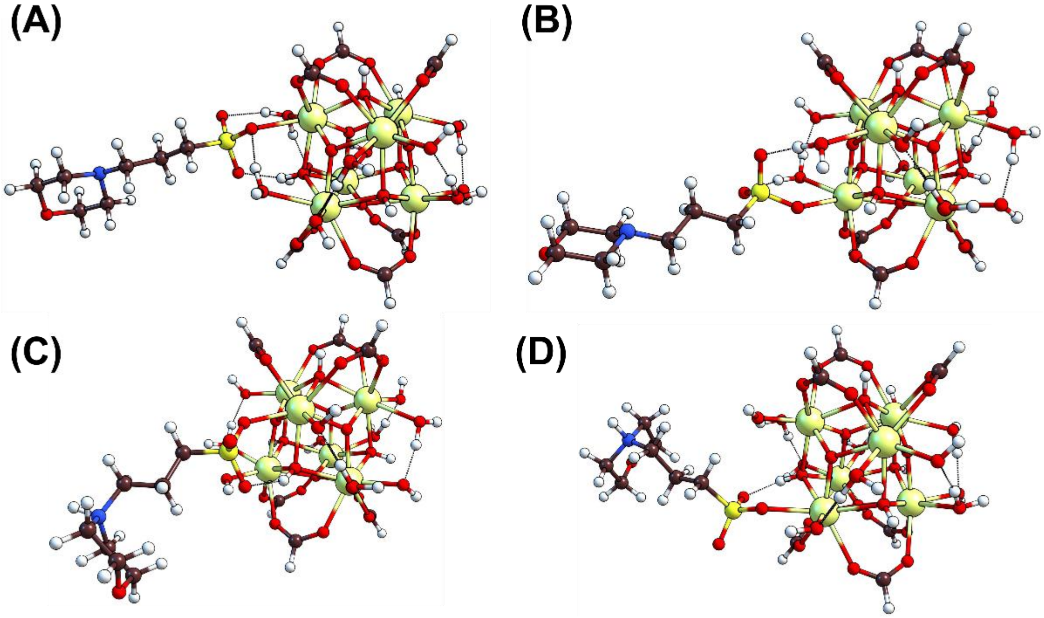

Figure S37. Optimized structures of oxidized Ce_6 nodes with node-bound MOPS.

Table S11. Calculated free energies of buffer insertion (ΔG_{Buf}) into Ce_6 cluster sites for borate, Tris, and MOPS.

Table Entry	Insertion site	ΔG_{Buf} (kcal mol^{-1})		
		Borate	Tris	MOPS
A	Terminal –OH-1	8.87	0.28	20.89
B	Terminal –OH-2	9.48	1.57	20.39
C	Terminal $–OH_2$-1	2.03	–2.11	1.54
D	Terminal $–OH_2$-2	–0.29	2.27	0.74
E	Terminal –OH-1 and $–OH_2$-1	4.00	–	–
F	Terminal –OH-2 and –OH-1	3.49	–	–

6.4 XYZ Coordinates

$Ce_6(H_2O)_6(OH)_6(\mu_3\text{–}OH)_4(\mu_3\text{–}O)_4(HCO_2)_6$

C	1.13097	-2.50622	3.972311
O	-2.55588	-0.87501	3.49507
O	-0.01381	-2.68075	3.487556
O	-1.48292	-3.89417	1.385105
Ce	-1.29901	-1.71248	1.607768
H	-1.18208	-4.45068	2.113656
O	3.614532	-2.28854	1.060925
O	2.657437	-3.22084	-1.29816
O	0.715728	-1.68798	0.713438
O	2.0399	-1.7694	3.499516
H	3.33216	-2.69661	0.202356
H	4.532638	-2.01908	0.932244
H	-0.50749	-4.25723	0.038266
H	3.337736	-0.43662	-1.08219
C	-2.73814	0.279987	3.969019
C	1.61258	2.242982	3.959081
O	-2.31621	1.358877	3.484802
O	-0.00026	-0.00046	2.654448
O	0.15509	4.277625	1.042916
O	0.520739	2.660546	3.483664
O	-1.81955	0.218029	0.713567
Ce	-0.83425	1.976412	1.605572
H	-3.25614	3.262917	2.102019
O	3.685353	2.197624	-0.78732
O	4.112028	0.654922	1.376527
O	1.098661	1.459998	0.710658
O	2.333393	1.332727	3.482012
O	2.434032	-0.3117	-0.76941
Ce	2.130503	-0.27213	1.604867
H	4.44471	1.192368	2.105502
H	3.940184	1.686267	0.025532
C	-1.51613	-2.15916	-3.88859
O	-3.79089	-1.99607	1.060571
O	-4.11625	-0.69601	-1.29866
O	-1.48827	-1.96187	-0.76435
O	-2.19274	-1.1918	-3.46246
H	-4.00029	-1.5438	0.203365
H	-4.00986	-2.9264	0.924088
H	-4.34025	-1.40442	-1.91546
O	2.117561	-1.3123	-3.46031
O	-0.46065	-2.61812	-3.3732
O	0.063159	-4.29744	-0.77465
Ce	0.854575	-1.98085	-1.46203
H	3.381014	-3.06032	-1.91706
H	0.790837	-4.8975	-0.57262
C	-1.10592	2.379909	-3.89374
O	-3.74806	2.085831	-0.78397
O	-2.62515	3.234865	1.372579
O	-0.95542	2.256525	-0.77163
O	-2.03199	1.692509	-3.38336
Ce	-2.13983	0.239826	-1.46523
H	0.654293	4.229854	0.187155
H	-0.55511	4.914797	0.895904
H	-3.42856	2.563802	0.026665
H	-4.63206	1.756992	-0.58155
H	-1.29977	3.101633	-1.08371
C	2.61608	-0.24416	-3.89086
O	2.484837	0.901461	-3.38006
O	-0.00291	-0.00741	-1.84622
O	1.450463	3.905422	-1.30614
O	0.067944	2.48389	-3.46239
Ce	1.278742	1.724173	-1.46531
H	0.958272	4.450442	-1.93308
H	3.851406	3.127868	-0.59379
H	-0.00019	-0.00821	3.618012
H	-2.04882	-2.68345	-1.07308
H	-1.87777	-2.66145	-4.8074
H	-1.35694	2.944892	-4.81303
H	3.231699	-0.30957	-4.80952
H	-3.33078	0.343995	4.902898
H	1.966132	2.732014	4.888324
H	1.371673	-3.05106	4.906524

$Ce_6(H_2O)_7(OH)_5(\mu_3\text{–}OH)_4(\mu_3\text{–}O)_4(HCO_2)_6$
Terminal –OH-1

C	1.148103	-2.55296	3.963339
O	-2.56973	-0.87556	3.523009
O	-0.01156	-2.70144	3.504589
O	-1.47243	-3.89407	1.393476
Ce	-1.29597	-1.68345	1.610055
H	-1.12824	-4.41244	2.130731
O	3.634821	-2.32697	1.035469
O	2.67316	-3.24564	-1.33443
O	0.726655	-1.6918	0.705475
O	2.065905	-1.84123	3.471822
H	3.346879	-2.72905	0.176062
H	4.545668	-2.0398	0.894843
H	-0.49056	-4.25391	0.068278
H	3.363976	-0.47059	-1.11369
C	-2.82859	0.271544	3.981802
C	1.802191	2.157855	4.032769
O	-2.47513	1.371197	3.489728
O	0.034262	-0.01246	2.686069
O	0.126087	4.402241	1.056108
O	0.766348	2.6813	3.552327
O	-1.86992	0.17321	0.714204
Ce	-0.81459	2.044423	1.714361
H	-3.59785	3.565291	2.047186
O	3.739553	2.180439	-0.80493
O	4.179071	0.608656	1.323521
O	1.21321	1.447763	0.699552
O	2.465722	1.211296	3.530803
O	2.465983	-0.34855	-0.78609
Ce	2.152144	-0.27853	1.591759
H	4.521216	1.126191	2.061909
H	3.990937	1.650221	0.000237
C	-1.52684	-2.1794	-3.87599
O	-3.78773	-2.03743	1.061745
O	-4.1122	-0.73762	-1.27313
O	-1.47376	-1.98859	-0.76303
O	-2.19591	-1.20736	-3.4514
H	-3.9918	-1.58418	0.199981
H	-3.96772	-2.97401	0.912914
H	-4.31051	-1.4326	-1.91291
O	2.108487	-1.30921	-3.4842
O	-0.46377	-2.63646	-3.37196
O	0.094023	-4.31437	-0.73682
Ce	0.859838	-1.98769	-1.45974
H	3.393498	-3.0274	-1.93925
H	0.827591	-4.89542	-0.50312
C	-1.08399	2.364306	-3.88674
O	-3.83942	2.065075	-0.89101
O	-2.93756	3.55793	1.340856
O	-0.96371	2.257214	-0.74901
O	-2.00758	1.671442	-3.37935
Ce	-2.12067	0.234613	-1.42345
H	0.613213	4.309663	0.196301
H	-0.52299	5.101989	0.915971
H	-3.61762	2.623384	-0.1211
H	-4.70758	1.683732	-0.70928
H	-1.27466	3.092445	-1.11538
C	2.611866	-0.23573	-3.89778
O	2.490713	0.902525	-3.37028
O	0.010104	-0.0213	-1.82696
O	1.445358	3.921368	-1.26574
O	0.084639	2.488326	-3.44715
Ce	1.29412	1.716663	-1.41537
H	0.997533	4.454664	-1.93394
H	3.899062	3.106045	-0.58578
H	0.051496	-0.05387	3.648399
H	-2.04173	-2.70226	-1.07434
H	-1.90089	-2.69137	-4.78497
H	-1.33418	2.9169	-4.81448
H	3.223783	-0.29103	-4.8203
H	-3.43347	0.303212	4.910983
H	2.176903	2.569277	4.992753
H	1.394812	-3.10321	4.893952
H	-2.82502	4.481649	1.079141

$Ce_6(H_2O)_7(OH)_5(\mu_3\text{–}OH)_4(\mu_3\text{–}O)_4(HCO_2)_6$
Terminal –OH-1

C	1.115368	-2.53009	3.974414
O	-2.56876	-0.88366	3.493558
O	-0.03016	-2.70985	3.490851
O	-1.53595	-3.91249	1.394232
Ce	-1.30669	-1.71875	1.59571
H	-1.23982	-4.45838	2.132472
O	3.62507	-2.31719	1.076726
O	2.64665	-3.29034	-1.24105
O	0.703476	-1.71197	0.712243
O	2.030438	-1.80902	3.492307
H	3.324614	-2.73997	0.228576
H	4.533277	-2.03181	0.916961
H	-0.54442	-4.28562	0.055511
H	3.364584	-0.55635	-1.06754
C	-2.72445	0.273732	3.974521
C	1.620375	2.191581	4.007024
O	-2.29777	1.347867	3.487376
O	-0.00201	-0.03246	2.661348
O	0.102051	4.344664	1.145324
O	0.545511	2.629959	3.513272
O	-1.79514	0.202227	0.693692
Ce	-0.7825	1.956762	1.57795
H	-3.23512	3.176658	2.112468
O	3.760014	2.306949	-0.72011
O	4.129717	0.655354	1.382177
O	1.131712	1.453223	0.777114
O	2.337226	1.270642	3.542336
O	2.466681	-0.3618	-0.77598
Ce	2.123558	-0.27538	1.592658
H	4.483445	1.142762	2.135529
H	3.977128	1.757084	0.079858
C	-1.53813	-2.22304	-3.8994
O	-3.81685	-1.94431	1.038491
O	-4.09366	-0.68747	-1.33744
O	-1.50629	-1.99814	-0.76044
O	-2.20031	-1.23527	-3.49673
H	-3.99921	-1.50079	0.167846
H	-4.06632	-2.86874	0.917213
H	-4.28393	-1.41437	-1.94349
O	2.138268	-1.47889	-3.48168
O	-0.4992	-2.69924	-3.36676
O	0.041729	-4.34609	-0.74483
Ce	0.843574	-2.01106	-1.46444
H	3.357796	-3.13687	-1.87553
H	0.786239	-4.9065	-0.49555
C	-1.13058	2.44356	-3.92923
O	-3.72952	2.067927	-0.7842
O	-2.61657	3.205801	1.372841
O	-0.90917	2.265306	-0.78423
O	-2.0112	1.695189	-3.41698
Ce	-2.07791	0.229526	-1.4962
H	0.5851	4.431299	0.300762
H	-0.6469	4.950708	1.078629
H	-3.40176	2.534308	0.031958
H	-4.58682	1.687918	-0.55792
H	-1.30085	3.088925	-1.09594
C	2.63846	-0.43983	-3.98689
O	2.52081	0.733538	-3.55313
O	-0.01374	-0.10304	-1.89875
O	1.450087	4.330473	-1.32999
O	0.057335	2.563414	-3.55042
Ce	1.38816	1.721229	-1.56965
H	0.916099	4.826189	-1.96579
H	3.980708	3.218782	-0.49668
H	-0.00309	-0.03238	3.624306
H	-2.07972	-2.70563	-1.07565
H	-1.8998	-2.73064	-4.81597
H	-1.45107	3.048305	-4.80162
H	3.240374	-0.57841	-4.90799
H	-3.29925	0.340586	4.920015
H	1.964886	2.671843	4.945074
H	1.349653	-3.06022	4.919549
H	2.30281	4.784097	-1.29173

$Ce_6(H_2O)_6(OH)_6(\mu_3-OH)_5(\mu_3-O)_3(HCO_2)_6$ μ_3–O-1

C	1.200156	-2.51653	4.093487
O	-2.62303	-0.8056	3.663945
O	0.060053	-2.72989	3.629086
O	-1.43089	-4.16113	1.250689
Ce	-1.38165	-1.8463	1.711485
H	-1.18106	-4.80959	1.916992
O	3.656568	-2.33683	1.04078
O	2.706675	-3.19839	-1.31021
O	0.818678	-1.72926	0.722004
O	2.097262	-1.78521	3.575105
H	3.379968	-2.72812	0.167402
H	4.585302	-2.09227	0.944009
H	-0.50957	-4.30656	0.019812
H	3.333864	-0.41559	-1.07287
C	-2.71218	0.354487	4.125955
C	1.588888	2.232932	3.944242
O	-2.30151	1.415503	3.572486
O	0.004281	0.019619	2.605725
O	0.054214	4.296094	1.025373
O	0.513182	2.659115	3.43592
O	-1.90017	0.321754	0.714934
Ce	-0.9679	1.948532	1.637917
H	-3.36599	3.36344	2.068835
O	3.69542	2.176602	-0.81109
O	4.080083	0.61804	1.412799
O	1.25922	1.651304	0.772033
O	2.309771	1.3051	3.504411
O	2.42774	-0.28461	-0.76937
Ce	2.148363	-0.39856	1.628672
H	4.363627	1.186848	2.13917
H	3.91863	1.66164	0.004385
C	-1.48779	-2.11346	-3.89812
O	-3.91429	-2.00891	1.073369
O	-4.11167	-0.70103	-1.31219
O	-1.46232	-1.92987	-0.76746
O	-2.17062	-1.15364	-3.46628
H	-4.06983	-1.55991	0.204887
H	-4.20771	-2.92015	0.951259
H	-4.3343	-1.38719	-1.95373
O	2.143153	-1.33067	-3.46667
O	-0.44102	-2.58418	-3.3752
O	0.09377	-4.28856	-0.8087
Ce	0.863446	-1.98887	-1.43432
H	3.403056	-3.01145	-1.95209
H	0.820473	-4.90026	-0.64316
C	-1.13806	2.385309	-3.92221
O	-3.75468	2.15177	-0.84859
O	-2.70303	3.267135	1.37504
O	-0.90499	2.248915	-0.76781
O	-2.0484	1.7143	-3.37368
Ce	-2.14054	0.256956	-1.43136
H	0.578531	4.2376	0.195493
H	-0.66715	4.907277	0.830844
H	-3.46775	2.618217	-0.02088
H	-4.65674	1.847872	-0.69116
H	-1.22925	3.109352	-1.05947
C	2.645648	-0.28215	-3.92858
O	2.515228	0.883629	-3.4538
O	0.029674	0.026146	-1.8421
O	1.471946	3.874787	-1.38158
O	0.055987	2.477066	-3.53444
Ce	1.288127	1.718722	-1.59449
H	0.95631	4.434373	-1.97695
H	3.858717	3.104141	-0.60228
H	0.036187	0.045953	3.568792
H	-2.00908	-2.63987	-1.12361
H	-1.8353	-2.59995	-4.83101
H	-1.41129	2.950174	-4.83471
H	3.265486	-0.3725	-4.84189
H	-3.20085	0.476703	5.113741
H	1.925955	2.737746	4.872105
H	1.46916	-3.00934	5.049447
H	1.833436	2.352789	1.11247

$Ce_6(H_2O)_6(OH)_6(\mu_3\text{–}OH)_5(\mu_3\text{–}O)_3(HCO_2)_6$ μ_3–O-2

C	1.144515	-2.51921	3.935363
O	-2.5569	-0.95491	3.52221
O	-0.00539	-2.69145	3.462482
O	-1.49182	-3.88302	1.378834
Ce	-1.31522	-1.68286	1.597344
H	-1.19643	-4.41052	2.130883
O	3.674254	-2.23945	1.061503
O	2.690438	-3.27287	-1.27293
O	0.71806	-1.6962	0.674978
O	2.052227	-1.78647	3.454723
H	3.402717	-2.68693	0.224272
H	4.564384	-1.90286	0.897724
H	-0.52794	-4.27705	0.083624
H	3.302057	-0.44891	-1.10997
C	-2.77641	0.179598	4.040607
C	1.709602	2.187104	4.041341
O	-2.43191	1.287362	3.573049
O	0.004174	0.000685	2.624344
O	0.099927	4.450808	1.036817
O	0.661467	2.700188	3.580951
O	-1.91885	0.182903	0.67349
Ce	-0.91177	2.124102	1.701094
H	-3.57595	3.418875	1.917125
O	3.776322	2.178766	-0.8236
O	4.14479	0.657226	1.329297
O	1.214936	1.509258	0.670089
O	2.389489	1.265087	3.5115
O	2.403358	-0.32906	-0.78077
Ce	2.129302	-0.2283	1.593513
H	4.508726	1.173014	2.058434
H	4.013798	1.662368	-0.00256
C	-1.55709	-2.18294	-3.82544
O	-3.79531	-2.07206	1.055228
O	-4.17087	-0.74704	-1.2729
O	-1.45688	-1.94797	-0.77289
O	-2.23426	-1.21038	-3.41624
H	-4.03734	-1.62252	0.205874
H	-3.9512	-3.01337	0.907872
H	-4.43019	-1.39388	-1.94053
O	2.145628	-1.35316	-3.4171
O	-0.47581	-2.61066	-3.32786
O	0.059214	-4.34523	-0.7231
Ce	0.88777	-2.08459	-1.41506
H	3.400164	-3.19642	-1.92299
H	0.75955	-4.97291	-0.50728
C	-1.09119	2.411547	-3.85062
O	-3.78397	2.122692	-0.81151
O	-2.89691	3.33445	1.239744
O	-0.9349	2.221211	-0.77879
O	-2.00135	1.688305	-3.35704
Ce	-2.24988	0.275445	-1.39032
H	0.622664	4.374501	0.201613
H	-0.60038	5.08639	0.843816
H	-3.48503	2.64862	0.026671
H	-4.70155	1.861115	-0.67109
H	-1.27125	3.046285	-1.14807
C	2.638385	-0.27564	-3.83823
O	2.472673	0.871455	-3.34169
O	-0.00115	-0.01227	-2.09172
O	1.480661	3.974166	-1.27375
O	0.082912	2.532255	-3.42248
Ce	1.347287	1.807425	-1.38622
H	1.116335	4.547476	-1.95925
H	4.009841	3.09867	-0.65044
H	0.016014	-0.01853	3.587916
H	-2.02371	-2.65594	-1.10133
H	-1.93503	-2.72519	-4.71458
H	-1.36024	2.992908	-4.75489
H	3.279831	-0.33768	-4.73951
H	-3.33194	0.176552	5.000312
H	2.085038	2.581179	5.008033
H	1.392776	-3.06504	4.867549
H	-0.0117	-0.00574	-3.05935

$Ce_6(H_2O)_5(OH)_7(\mu_3\text{–}OH)_4(\mu_3\text{–}O)_4(HCO_2)_6^-$ Terminal $-OH_2$-1

C	1.054848	-2.49288	4.029445
O	-2.5437	-0.79861	3.624722
O	-0.1078	-2.6409	3.587976
O	-1.23897	-3.98628	1.409852
Ce	-1.42549	-1.74683	1.615136
H	-0.63944	-4.36979	2.061206
O	3.559368	-2.33474	1.061879
O	2.633726	-3.23202	-1.3035
O	0.671461	-1.70205	0.692798
O	1.975998	-1.80099	3.50793
H	3.281665	-2.72793	0.191963
H	4.473152	-2.04854	0.939775
H	-0.47494	-4.25409	0.012604
H	3.315881	-0.46483	-1.07657
C	-2.71212	0.371966	4.04664
C	1.590243	2.218371	3.958115
O	-2.33049	1.433831	3.4841
O	-0.04976	-0.00458	2.638631
O	0.150655	4.282378	1.03176
O	0.513875	2.658256	3.46844
O	-1.8415	0.254091	0.687606
Ce	-0.86039	1.96755	1.588347
H	-3.27798	3.277251	2.058463
O	3.680632	2.161212	-0.75796
O	4.071224	0.604107	1.394031
O	1.083754	1.45289	0.703633
O	2.298806	1.291967	3.49425
O	2.411276	-0.33197	-0.77121
Ce	2.06071	-0.31684	1.598998
H	4.362425	1.154508	2.130822
H	3.911836	1.636403	0.055797
C	-1.52169	-2.12482	-3.93469
O	-3.53131	-2.10079	1.223448
O	-4.14276	-0.62989	-1.3648
O	-1.52184	-1.95264	-0.80162
O	-2.20357	-1.16537	-3.501
H	-3.90025	-1.65948	0.440839
H	-4.32232	-1.44798	-1.84449
O	2.118331	-1.31859	-3.46318
O	-0.47181	-2.59375	-3.41607
O	0.016839	-4.30174	-0.86476
Ce	0.813072	-1.98126	-1.4633
H	3.353648	-3.03161	-1.91461
H	0.745653	-4.92041	-0.73811
C	-1.09667	2.400473	-3.90804
O	-3.75913	2.131118	-0.81748
O	-2.64421	3.269059	1.331253
O	-0.95697	2.275321	-0.79073
O	-2.03025	1.719941	-3.40441
Ce	-2.16312	0.255265	-1.47206
H	0.656648	4.223729	0.179949
H	-0.57057	4.900867	0.860303
H	-3.432	2.603649	-0.00337
H	-4.64327	1.805873	-0.61003
H	-1.29242	3.122267	-1.10577
C	2.627459	-0.25331	-3.88608
O	2.493802	0.894717	-3.37946
O	-0.01468	0.001759	-1.86676
O	1.471988	3.900804	-1.30168
O	0.078235	2.49381	-3.47453
Ce	1.269553	1.709848	-1.46407
H	0.972112	4.439291	-1.92803
H	3.841638	3.087878	-0.54456
H	-0.04899	-0.01369	3.601547
H	-2.09255	-2.65769	-1.12762
H	-1.87226	-2.61175	-4.86662
H	-1.3391	2.971503	-4.8265
H	3.257156	-0.32139	-4.79549
H	-3.25261	0.486503	5.008113
H	1.943275	2.703706	4.890144
H	1.31029	-3.01876	4.971681

$Ce_6(H_2O)_5(OH)_7(\mu_3\text{–}OH)_4(\mu_3\text{–}O)_4(HCO_2)_6^-$ Terminal –OH_2-2

C	1.121347	-2.51249	4.004375
O	-2.57382	-0.86546	3.517781
O	-0.02077	-2.69247	3.516919
O	-1.61846	-3.87295	1.494269
Ce	-1.31854	-1.72403	1.617485
H	-1.13427	-4.43835	2.107972
O	3.593928	-2.32997	1.071901
O	2.862837	-3.10563	-1.37992
O	0.695667	-1.71062	0.747183
O	2.033956	-1.78083	3.529516
H	3.382009	-2.67649	0.159274
H	4.524532	-2.07571	1.047258
H	-0.41922	-4.23109	-0.2205
H	3.321433	-0.47744	-1.06541
C	-2.74697	0.292751	3.985151
C	1.610762	2.234827	3.962178
O	-2.32518	1.368521	3.492272
O	-0.01371	-0.00911	2.671038
O	0.192023	4.253851	1.018728
O	0.520837	2.656938	3.485017
O	-1.835	0.220921	0.729353
Ce	-0.83412	1.960859	1.601988
H	-3.25029	3.262915	2.098573
O	3.682405	2.153514	-0.77982
O	4.104528	0.620279	1.392757
O	1.090568	1.43563	0.716898
O	2.328103	1.319904	3.490535
O	2.418296	-0.34765	-0.75547
Ce	2.106544	-0.30729	1.605702
H	4.405032	1.176518	2.121431
H	3.927448	1.642584	0.036484
C	-1.57082	-2.13681	-3.91672
O	-3.83493	-1.95822	1.078884
O	-4.15189	-0.66556	-1.27695
O	-1.53349	-1.9645	-0.74797
O	-2.26071	-1.18848	-3.46154
H	-4.03143	-1.50275	0.218742
H	-4.05274	-2.88775	0.936312
H	-4.36119	-1.38486	-1.886
O	2.071429	-1.346	-3.51086
O	-0.51378	-2.59909	-3.41898

O	0.258111	-4.13819	-0.90947
Ce	0.843977	-2.11916	-1.44585
H	3.538415	-2.67638	-1.91904
C	-1.10948	2.359026	-3.90588
O	-3.7468	2.095948	-0.78675
O	-2.61791	3.249133	1.370094
O	-0.95605	2.241725	-0.7763
O	-2.04331	1.686412	-3.39138
Ce	-2.13166	0.221501	-1.4623
H	0.685546	4.180685	0.159205
H	-0.50796	4.899809	0.862567
H	-3.41841	2.572724	0.02075
H	-4.61695	1.744401	-0.56388
H	-1.28864	3.088247	-1.09553
C	2.605038	-0.28852	-3.91286
O	2.503072	0.855339	-3.38306
O	-0.02533	-0.03961	-1.83399
O	1.470386	3.849461	-1.32629
O	0.070888	2.437686	-3.48649
Ce	1.266168	1.646241	-1.47554
H	0.94616	4.369312	-1.94846
H	3.80908	3.086355	-0.57019
H	-0.0134	-0.01141	3.634211
H	-2.08097	-2.69941	-1.04651
H	-1.93199	-2.61225	-4.85088
H	-1.35882	2.935316	-4.81914
H	3.22991	-0.34982	-4.82688
H	-3.33232	0.365743	4.923575
H	1.965145	2.726668	4.890221
H	1.35841	-3.04786	4.945597

$Ce_6(H_2O)_6(OH)_5(O)(\mu_3–OH)_4(\mu_3–O)_4(HCO_2)_6^–$ Terminal –OH-1

C	1.24955	-2.45221	4.062254
O	-2.59142	-0.91481	3.577594
O	0.105034	-2.67599	3.614648
O	-1.52662	-3.74071	1.473646
Ce	-1.3401	-1.87258	1.638265
O	3.650378	-2.30783	1.019618
O	2.657452	-3.25709	-1.31721
O	0.777102	-1.73275	0.679389
O	2.144084	-1.73595	3.520585
H	3.350424	-2.71516	0.165661
H	4.553442	-2.00585	0.861593
H	-0.53771	-4.22151	0.084246
H	3.357418	-0.43715	-1.11089
C	-2.73675	0.249197	4.028501
C	1.602486	2.23442	3.942892
O	-2.32604	1.317497	3.501416
O	0.004456	-0.03347	2.638259
O	0.11239	4.255797	1.058717
O	0.497941	2.631853	3.480579
O	-1.85199	0.236455	0.682172
Ce	-0.86661	1.901287	1.596348
H	-3.30296	3.176234	2.093146
O	3.700466	2.192876	-0.77386
O	4.120832	0.638034	1.363346
O	1.102608	1.440329	0.694099
O	2.33828	1.340089	3.459479
O	2.455861	-0.31408	-0.79354
Ce	2.115525	-0.30722	1.581711
H	4.403597	1.189182	2.102818
H	3.941029	1.665727	0.03718
C	-1.4934	-2.15284	-3.91883
O	-3.90124	-1.92116	1.045568
O	-4.13775	-0.67414	-1.35842
O	-1.48654	-1.9532	-0.81479
O	-2.16536	-1.17979	-3.50039
H	-4.04832	-1.484	0.168603
H	-4.17559	-2.83784	0.923306
H	-4.33415	-1.39896	-1.96475
O	2.136591	-1.30836	-3.47621
O	-0.44483	-2.6226	-3.39698
O	0.022051	-4.31011	-0.72677
Ce	0.854934	-1.97888	-1.45537
H	3.363164	-3.10219	-1.95729
H	0.748126	-4.90031	-0.49204
C	-1.09442	2.403473	-3.89496
O	-3.75301	2.109262	-0.81051
O	-2.66406	3.207893	1.371251
O	-0.94921	2.262113	-0.78191
O	-2.01652	1.704958	-3.39544
Ce	-2.12693	0.242267	-1.45462
H	0.623365	4.208028	0.210604
H	-0.62758	4.849451	0.879902
H	-3.43368	2.565032	0.016868
H	-4.63204	1.764907	-0.61266
H	-1.29289	3.110132	-1.08411
C	2.634077	-0.23849	-3.90192
O	2.497358	0.90856	-3.3941
O	0.016619	0.002867	-1.86215
O	1.462773	3.914721	-1.27627
O	0.08097	2.508129	-3.46533
Ce	1.286834	1.719254	-1.46059
H	0.957039	4.450813	-1.89983
H	3.851455	3.120301	-0.55669
H	0.004663	-0.03614	3.601227
H	-2.02058	-2.67877	-1.15584
H	-1.85229	-2.65417	-4.84017
H	-1.34865	2.981597	-4.80594
H	3.256607	-0.30157	-4.81692
H	-3.28508	0.350358	4.9877
H	1.95508	2.730203	4.870025
H	1.523986	-2.92258	5.028937

$Ce_6(H_2O)_6(OH)_5(O)(\mu_3\text{–}OH)_4(\mu_3\text{–}O)_4(HCO_2)_6^-$
Terminal –OH-2

C	1.154577	-2.52174	3.979276
O	-2.56157	-0.91207	3.520482
O	0.010616	-2.70146	3.496887
O	-1.48721	-3.91459	1.410109
Ce	-1.29756	-1.70904	1.603064
H	-1.15864	-4.44059	2.149042
O	3.628802	-2.29433	1.050186
O	2.672341	-3.23141	-1.29032
O	0.735299	-1.69402	0.727513
O	2.062604	-1.78172	3.507208
H	3.333194	-2.69581	0.189275
H	4.539931	-2.00972	0.906678
H	-0.47539	-4.26675	0.078808
H	3.348837	-0.43322	-1.08865
C	-2.7405	0.241645	4.000239
C	1.610158	2.241639	3.966489
O	-2.3182	1.324284	3.525326
O	0.015068	-0.01725	2.681245
O	0.165687	4.255078	1.042449
O	0.515605	2.651206	3.492889
O	-1.83098	0.190637	0.785306
Ce	-0.84727	1.933988	1.604651
H	-3.2653	3.22504	2.148441
O	3.656285	2.205406	-0.79844
O	4.12318	0.664923	1.372362
O	1.097253	1.439424	0.720414
O	2.342144	1.339765	3.488188
O	2.446974	-0.31807	-0.76936
Ce	2.130988	-0.27633	1.604502
H	4.424728	1.2263	2.096578
H	3.910667	1.69371	0.013704
C	-1.43614	-2.25132	-3.94291
O	-3.80429	-1.99354	1.012822
O	-3.99401	-0.57675	-1.31746
O	-1.4675	-2.00781	-0.75453
O	-2.11393	-1.27064	-3.56648
H	-3.9764	-1.50483	0.168325
H	-4.01587	-2.91641	0.824875
O	2.144902	-1.32354	-3.47353
O	-0.40743	-2.71961	-3.37313
O	0.107201	-4.31948	-0.72483
Ce	0.848191	-1.96973	-1.46177
H	3.386725	-3.02921	-1.9074
H	0.858243	-4.87454	-0.48388
C	-1.13949	2.389517	-3.93556
O	-3.76342	2.268782	-0.80723
O	-2.61108	3.26421	1.44069
O	-0.96841	2.238783	-0.75672
O	-2.08385	1.70665	-3.46601
Ce	-2.28191	0.189722	-1.50292
H	0.652336	4.195524	0.178014
H	-0.5557	4.878053	0.89031
H	-3.40817	2.677546	0.022358
H	-4.65312	1.966917	-0.59175
H	-1.30639	3.085078	-1.06981
C	2.630701	-0.2493	-3.90355
O	2.479858	0.897136	-3.39999
O	0.022519	-0.03638	-1.86118
O	1.432859	3.891007	-1.31516
O	0.029538	2.474665	-3.47556
Ce	1.225393	1.683622	-1.47879
H	0.892013	4.408698	-1.92476
H	3.767843	3.138432	-0.58113
H	0.017191	-0.02633	3.644133
H	-2.01226	-2.74627	-1.04865
H	-1.75867	-2.76997	-4.86885
H	-1.35379	2.977965	-4.85136
H	3.254453	-0.30954	-4.81783
H	-3.3344	0.300619	4.934506
H	1.960341	2.731129	4.897491
H	1.399388	-3.06694	4.913041

$Ce_6(H_2O)_6(OH)_6(\mu_3\text{–}OH)_3(\mu_3\text{–}O)_5(HCO_2)_6^-$ μ_3–OH-1

C	1.119608	-2.44046	4.036318
O	-2.52556	-0.89761	3.538114
O	-0.00999	-2.65175	3.530454
O	-1.46527	-3.88114	1.383516
Ce	-1.2355	-1.65459	1.616965
H	-1.08656	-4.40725	2.097753
O	3.612779	-2.27066	1.07403
O	2.658424	-3.24396	-1.29941
O	0.7553	-1.80983	0.684991
O	2.038122	-1.72948	3.546361
H	3.326437	-2.67296	0.215881
H	4.506788	-1.94148	0.919136
H	-0.5434	-4.23842	0.020819
H	3.326273	-0.45431	-1.05262
C	-2.67084	0.251546	4.035696
C	1.563797	2.208207	4.019351
O	-2.28647	1.335841	3.532534
O	-0.00182	-5.7E-05	2.41831
O	0.131221	4.269517	1.049969
O	0.491666	2.648975	3.52413
O	-1.94833	0.244695	0.68405
Ce	-0.815	1.889456	1.614706
H	-3.27698	3.131962	2.089396
O	3.731051	2.154199	-0.82224
O	4.089271	0.664402	1.374766
O	1.185755	1.558755	0.681434
O	2.304448	1.32242	3.526008
O	2.412682	-0.31939	-0.7773
Ce	2.04393	-0.24436	1.61661
H	4.357522	1.25173	2.091291
H	3.940428	1.644428	0.010455
C	-1.53931	-2.16214	-3.90193
O	-3.77044	-2.01717	1.066778
O	-4.13768	-0.68686	-1.29713
O	-1.48339	-1.93916	-0.77342
O	-2.2104	-1.19045	-3.47617
H	-3.97452	-1.56087	0.212047
H	-3.90916	-2.95681	0.894454
H	-4.36427	-1.3859	-1.92287
O	2.123796	-1.32521	-3.47792
O	-0.47569	-2.61688	-3.40062
O	0.003007	-4.31709	-0.81153
Ce	0.847005	-1.98977	-1.46807
H	3.37945	-3.08252	-1.92037
H	0.725477	-4.92225	-0.60635
C	-1.09793	2.398661	-3.90978
O	-3.72792	2.158509	-0.82569
O	-2.62937	3.203521	1.378128
O	-0.93739	2.243027	-0.78017
O	-2.02362	1.702472	-3.41153
Ce	-2.14625	0.253575	-1.46975
H	0.634557	4.215165	0.198893
H	-0.61637	4.852543	0.868755
H	-3.38997	2.58952	0.009869
H	-4.62263	1.854464	-0.63281
H	-1.27835	3.100846	-1.05691
C	2.627769	-0.25918	-3.90801
O	2.491081	0.890409	-3.40842
O	-0.0029	-0.00664	-1.84128
O	1.47208	3.91831	-1.30724
O	0.076762	2.497861	-3.47901
Ce	1.292572	1.722083	-1.47115
H	0.978933	4.457956	-1.93744
H	3.902745	3.082414	-0.62446
H	-2.05452	-2.66495	-1.04867
H	-1.91574	-2.67308	-4.81072
H	-1.34978	2.978083	-4.82054
H	3.254663	-0.33138	-4.81921
H	-3.1954	0.307352	5.0108
H	1.884152	2.652222	4.98346
H	1.335592	-2.93006	5.007306

$Ce_6(H_2O)_6(OH)_6(\mu_3\text{–}OH)_3(\mu_3\text{–}O)_5(HCO_2)_6^-$ μ_3–OH-2

C	1.131269	-2.51428	3.960971
O	-2.55731	-0.87744	3.460248
O	-0.01528	-2.67005	3.479891
O	-1.51993	-3.91344	1.392403
Ce	-1.28917	-1.70003	1.512937
H	-1.16752	-4.42681	2.129101
O	3.659261	-2.26679	1.02955
O	2.622249	-3.21758	-1.27508
O	0.808457	-1.7537	0.776702
O	2.047621	-1.76882	3.508877
H	3.330673	-2.67144	0.181136
H	4.564171	-1.98376	0.849077
H	-0.56095	-4.26495	0.054478
H	3.308429	-0.46812	-1.09662
C	-2.73376	0.264523	3.961257
C	1.611057	2.23905	3.985716
O	-2.289	1.356715	3.522777
O	0.006601	0.004876	2.629658
O	0.169139	4.295212	1.043151
O	0.532231	2.67482	3.497918
O	-1.90461	0.289554	0.776521
Ce	-0.83444	1.980528	1.613329
H	-3.27528	3.239514	2.107674
O	3.703174	2.205198	-0.77807
O	4.138405	0.677944	1.394689
O	1.102734	1.460607	0.71179
O	2.334821	1.33108	3.508404
O	2.42299	-0.29647	-0.75738
Ce	2.141714	-0.26815	1.607682
H	4.424483	1.255413	2.112562
H	3.94586	1.691232	0.03831
C	-1.54006	-2.22184	-3.87338
O	-3.81096	-2.00115	1.016842
O	-4.14714	-0.6821	-1.30207
O	-1.35107	-1.78971	-0.69591
O	-2.21396	-1.251	-3.45154
H	-4.02431	-1.53749	0.162426
H	-4.00683	-2.93354	0.863132
H	-4.35613	-1.3869	-1.92721
O	2.128972	-1.31674	-3.44706
O	-0.46846	-2.66228	-3.37557
O	0.006332	-4.31286	-0.76358
Ce	0.776572	-1.96148	-1.41575
H	3.314619	-3.0251	-1.91916
H	0.747194	-4.89201	-0.54858
C	-1.11078	2.373954	-3.89142
O	-3.73617	2.093815	-0.7943
O	-2.62272	3.27179	1.397833
O	-0.93716	2.251121	-0.75868
O	-2.01177	1.658514	-3.37986
Ce	-2.08969	0.175579	-1.42491
H	0.66805	4.234178	0.186621
H	-0.54572	4.9238	0.883442
H	-3.41314	2.562789	0.016685
H	-4.5956	1.719891	-0.56628
H	-1.31119	3.072036	-1.09737
C	2.621114	-0.2533	-3.88888
O	2.504706	0.900867	-3.38505
O	0.0372	0.047231	-1.95255
O	1.474029	3.929218	-1.29883
O	0.056672	2.540524	-3.4547
Ce	1.285465	1.728477	-1.47237
H	0.957544	4.460028	-1.91804
H	3.854533	3.135221	-0.57194
H	-0.01704	-0.03838	3.591357
H	-1.91927	-2.74548	-4.77385
H	-1.37472	2.91386	-4.82329
H	3.223669	-0.32213	-4.81717
H	-3.34557	0.311705	4.884943
H	1.9517	2.71003	4.929641
H	1.375684	-3.08554	4.879571

$Ce_6(H_2O)_6(OH)_6(\mu_3\text{–}OH)_4(\mu_3\text{–}O)_4(HCO_2)_6$ with H_2O Proximal to Terminal –OH-1

C	1.402573	-2.53662	3.86057
O	-2.36177	-1.04729	3.490991
O	0.25192	-2.74162	3.393628
O	-1.28572	-3.94787	1.263375
Ce	-1.13885	-1.78734	1.545621
O	3.772575	-2.17708	0.913969
O	2.816159	-3.09579	-1.45349
O	0.862355	-1.66982	0.622074
O	2.255803	-1.73085	3.40646
H	3.495913	-2.58248	0.052564
H	4.684594	-1.88715	0.786856
H	-0.305	-4.27475	-0.08894
H	3.404341	-0.29138	-1.19782
C	-2.58059	0.091889	3.98906
C	1.675981	2.231853	3.927003
O	-2.22279	1.1956	3.509847
O	0.124403	-0.04667	2.604821
O	0.106811	4.254359	1.08503
O	0.557052	2.611608	3.483679
O	-1.73783	0.14275	0.701591
Ce	-0.8018	1.917789	1.611899
H	-3.25222	3.111119	2.193652
O	3.660875	2.352937	-0.85881
O	4.182259	0.78564	1.263638
O	1.132497	1.491107	0.672128
O	2.421955	1.359469	3.418783
O	2.503147	-0.20336	-0.86579
Ce	2.239776	-0.21671	1.513551
H	4.518326	1.303271	2.005259
H	3.950115	1.834918	-0.06153
C	-1.43346	-2.13205	-3.95098
O	-3.64235	-2.09032	1.055258
O	-4.04112	-0.80875	-1.30139
O	-1.35773	-1.99104	-0.82678
O	-2.13539	-1.19651	-3.49594
H	-3.88338	-1.65157	0.199867
H	-3.87427	-3.02083	0.945992
H	-4.2327	-1.52564	-1.91931
O	2.174319	-1.16376	-3.56619
O	-0.35327	-2.56251	-3.46227
O	0.273862	-4.27252	-0.89659
Ce	0.970201	-1.91522	-1.56017
H	3.525511	-2.89088	-2.07579
H	1.025874	-4.84324	-0.69862
C	-1.19249	2.426005	-3.86911
O	-3.76908	1.95945	-0.71344
O	-2.64618	3.107612	1.442907
O	-0.97811	2.242696	-0.75482
O	-2.08107	1.691145	-3.35765
Ce	-2.09981	0.199115	-1.47061
H	0.588713	4.252719	0.21811
H	-0.6056	4.898526	0.989076
H	-3.45118	2.434589	0.099611
H	-4.62751	1.578467	-0.49257
H	-1.35514	3.082251	-1.04321
C	2.623211	-0.07016	-3.98688
O	2.456678	1.061234	-3.45434
O	0.037381	0.035698	-1.89286
O	1.356342	3.985934	-1.30263
O	-0.01474	2.563918	-3.45852
Ce	1.261908	1.80225	-1.5025
H	0.819212	4.514308	-1.9066
H	3.790006	3.284618	-0.64461
H	0.146782	-0.07309	3.567634
H	-1.90173	-2.72319	-1.1401
H	-1.79376	-2.63074	-4.87224
H	-1.48391	3.003502	-4.7684
H	3.224593	-0.09598	-4.91679
H	-3.14635	0.114939	4.941268
H	2.031215	2.720463	4.855817
H	1.694098	-3.12124	4.754649
O	-0.87375	-5.20498	3.946597
H	-0.48624	-4.3074	3.924674
H	-1.68699	-5.11362	4.45755
H	-1.1693	-4.55173	2.012455

$Ce_6(H_2O)_7(OH)_5(\mu_3\text{–}OH)_4(\mu_3\text{–}O)_4(HCO_2)_6$
Terminal –OH-1with Proximal H_2O

C	1.172915	-2.39772	4.116927
O	-2.63055	-0.83947	3.582503
O	0.014516	-2.585	3.689073
O	-1.5453	-3.71296	1.532175
Ce	-1.37412	-1.82326	1.665023
O	3.608684	-2.3153	1.072844
O	2.620787	-3.27386	-1.26101
O	0.740994	-1.72877	0.718356
O	2.085265	-1.72455	3.550548
H	3.313874	-2.73291	0.222053
H	4.521007	-2.03676	0.926174
H	-0.57955	-4.19541	0.107389
H	3.338759	-0.4677	-1.07376
C	-2.76747	0.328226	4.02635
C	1.588619	2.262992	3.946233
O	-2.33626	1.388751	3.500118
O	-0.02324	0.006578	2.65117
O	0.12242	4.276878	1.031349
O	0.49605	2.67466	3.468136
O	-1.86601	0.264593	0.691599
Ce	-0.87346	1.939432	1.590776
H	-3.29811	3.255881	2.066411
O	3.698804	2.173498	-0.76418
O	4.098432	0.634565	1.389433
O	1.094075	1.45053	0.701709
O	2.313849	1.350919	3.479806
O	2.436843	-0.32911	-0.76369
Ce	2.089172	-0.29879	1.608634
H	4.382078	1.190618	2.124961
H	3.930732	1.650967	0.052308
C	-1.51202	-2.16294	-3.891
O	-3.91925	-1.88071	1.068294
O	-4.15129	-0.66058	-1.34943
O	-1.51373	-1.94164	-0.78299
O	-2.18086	-1.18462	-3.47931
H	-4.0848	-1.45476	0.189023
H	-4.22828	-2.78986	0.977207
H	-4.31526	-1.40145	-1.9463
O	2.123144	-1.34645	-3.4387
O	-0.46807	-2.63547	-3.36286
O	-0.03324	-4.31051	-0.70695
Ce	0.827948	-1.98852	-1.42037
H	3.327959	-3.12929	-1.90194
H	0.685716	-4.90423	-0.45795
C	-1.08031	2.384852	-3.90352
O	-3.76043	2.122854	-0.821
O	-2.66379	3.248515	1.339916
O	-0.94892	2.269658	-0.79053
O	-2.01007	1.699043	-3.40009
Ce	-2.1382	0.253076	-1.44867
H	0.636816	4.220252	0.18557
H	-0.60718	4.881908	0.848359
H	-3.43669	2.5912	-0.00201
H	-4.63673	1.778276	-0.61144
H	-1.28145	3.118597	-1.10264
C	2.628465	-0.28369	-3.87312
O	2.49749	0.868801	-3.37613
O	0.004782	-0.00625	-1.84451
O	1.474741	3.901881	-1.29503
O	0.094024	2.484565	-3.46986
Ce	1.287597	1.705463	-1.45557
H	0.963651	4.425541	-1.92494
H	3.845677	3.102053	-0.54847
H	-0.02939	0.009302	3.614068
H	-2.05649	-2.66378	-1.11788
H	-1.86987	-2.6659	-4.81178
H	-1.326	2.955104	-4.82171
H	3.253005	-0.35914	-4.78575
H	-3.32783	0.440425	4.977214
H	1.941123	2.762216	4.871461
H	1.443318	-2.86164	5.087614
O	0.359824	-5.51625	2.407016
H	1.013437	-5.0212	2.914299
H	-0.31316	-4.84712	2.152321

$Ce_6(H_2O)_6(OH)_6(\mu_3–OH)_4(\mu_3–O)_4(HCO_2)_6$ with H_2O Proximal to Terminal –OH-2

C	1.221598	-2.53195	3.992039
O	-2.47766	-0.9084	3.599359
O	0.0639	-2.70245	3.537327
O	-1.41617	-3.92133	1.448971
Ce	-1.2592	-1.73786	1.681748
H	-1.08754	-4.47755	2.165565
O	3.632743	-2.29814	1.018454
O	2.635225	-3.20688	-1.33547
O	0.732647	-1.69889	0.739496
O	2.120098	-1.79711	3.496812
H	3.338905	-2.70172	0.161825
H	4.552806	-2.03872	0.883755
H	-0.49033	-4.2665	0.070355
H	3.309881	-0.42698	-1.10699
C	-2.65111	0.243162	4.084451
C	1.694291	2.209077	3.993
O	-2.24141	1.326078	3.597991
O	0.057876	-0.0264	2.70863
O	0.172171	4.257762	1.123391
O	0.593242	2.629792	3.54257
O	-1.79933	0.197606	0.811216
Ce	-0.80367	1.954392	1.69043
H	-3.23066	3.210265	2.22926
O	3.653202	2.213506	-0.79424
O	4.135836	0.651413	1.341674
O	1.110867	1.450356	0.751293
O	2.404413	1.299988	3.497333
O	2.413731	-0.30662	-0.77145
Ce	2.164366	-0.28637	1.611474
H	4.49231	1.172923	2.070948
H	3.928305	1.695287	0.00799
C	-1.58529	-2.12594	-3.82801
O	-3.75296	-2.05267	1.203697
O	-4.12378	-0.71444	-1.08111
O	-1.51125	-1.96731	-0.69188
O	-2.25076	-1.15942	-3.38496
H	-4.00159	-1.57683	0.36711
H	-3.96852	-2.98109	1.051358
O	2.037517	-1.2834	-3.46363
O	-0.52872	-2.59973	-3.32663
O	0.057691	-4.29758	-0.75916
Ce	0.822644	-1.97448	-1.44069
H	3.344223	-3.01908	-1.96364
H	0.795875	-4.89224	-0.57994
C	-1.1942	2.400255	-3.81216
O	-3.74145	2.137387	-0.69922
O	-2.60388	3.208582	1.495926
O	-0.97836	2.248184	-0.68264
O	-2.10636	1.711142	-3.2755
Ce	-2.17697	0.232661	-1.35724
H	0.643959	4.228191	0.251687
H	-0.52768	4.914666	1.021226
H	-3.41172	2.588165	0.122459
H	-4.66309	1.908633	-0.53263
H	-1.32438	3.097326	-0.98161
C	2.523956	-0.21084	-3.89627
O	2.400843	0.93042	-3.37296
O	-0.05085	-0.00047	-1.78745
O	1.399058	3.911851	-1.26644
O	-0.01609	2.510292	-3.40164
Ce	1.238627	1.730675	-1.42538
H	0.877181	4.447008	-1.87783
H	3.814753	3.142779	-0.59192
H	0.078331	-0.03942	3.671852
H	-2.0695	-2.69093	-1.00053
H	-1.95758	-2.61498	-4.74959
H	-1.46782	2.955707	-4.73
H	3.119429	-0.2666	-4.82868
H	-3.22346	0.300899	5.031213
H	2.068571	2.69382	4.916329
H	1.485036	-3.07957	4.918481
O	-4.75317	1.370325	-4.14239
H	-3.81585	1.580577	-3.97337
H	-4.96448	1.815516	-4.97067
H	-4.98232	-0.2955	-1.20987

$Ce_6(H_2O)_7(OH)_5(\mu_3{-}OH)_4(\mu_3{-}O)_4(HCO_2)_6$
Terminal –OH-2with Proximal H_2O

C	1.156519	-2.54129	3.981083
O	-2.54262	-0.89334	3.524962
O	0.010327	-2.70552	3.49826
O	-1.49497	-3.89209	1.39553
Ce	-1.28323	-1.69248	1.607165
H	-1.19565	-4.43438	2.13502
O	3.635171	-2.32077	1.058336
O	2.677305	-3.22793	-1.29824
O	0.74866	-1.68916	0.731638
O	2.073633	-1.81074	3.511952
H	3.343798	-2.71642	0.194289
H	4.555378	-2.0599	0.928861
H	-0.48286	-4.24598	0.067222
H	3.378585	-0.44139	-1.06998
C	-2.71197	0.258715	4.011788
C	1.657803	2.210576	4.003253
O	-2.27795	1.339959	3.544123
O	0.042482	-0.01857	2.694462
O	0.238596	4.26263	1.089463
O	0.570322	2.637397	3.528466
O	-1.79493	0.218189	0.795811
Ce	-0.79811	1.949831	1.633091
H	-3.20857	3.22497	2.178977
O	3.708128	2.197852	-0.75886
O	4.156262	0.636887	1.397118
O	1.141302	1.441395	0.747392
O	2.380452	1.303752	3.520005
O	2.477393	-0.31909	-0.75135
Ce	2.15772	-0.29057	1.621337
H	4.469483	1.184028	2.127193
H	3.955569	1.676682	0.049827
C	-1.4268	-2.18558	-3.93812
O	-3.79406	-1.94444	1.011683
O	-3.97169	-0.50451	-1.31031
O	-1.45513	-1.97148	-0.7544
O	-2.09234	-1.19847	-3.55539
H	-3.96552	-1.45357	0.169605
H	-4.01826	-2.86468	0.825802
O	2.166393	-1.30462	-3.4633
O	-0.40462	-2.67075	-3.37147
O	0.103522	-4.29956	-0.73376

Ce	0.864832	-1.95447	-1.4573
H	3.392717	-3.02021	-1.91235
H	0.85202	-4.85594	-0.4878
C	-1.09063	2.437892	-3.90018
O	-3.69315	2.3263	-0.81132
O	-2.55973	3.282335	1.467352
O	-0.91187	2.268018	-0.72601
O	-2.04365	1.765724	-3.43193
Ce	-2.22839	0.240195	-1.48292
H	0.723745	4.211846	0.224927
H	-0.46283	4.912225	0.95763
H	-3.35461	2.731494	0.025963
H	-4.60245	2.059836	-0.63473
H	-1.23827	3.120394	-1.0351
C	2.661111	-0.23179	-3.88646
O	2.521283	0.912524	-3.37491
O	0.055729	-0.00742	-1.84057
O	1.498548	3.903191	-1.28109
O	0.07983	2.505995	-3.44176
Ce	1.279006	1.700738	-1.44931
H	0.951811	4.421228	-1.88525
H	3.827539	3.127871	-0.53286
H	0.043912	-0.03294	3.657317
H	-2.0071	-2.7013	-1.05653
H	-1.75644	-2.69354	-4.86728
H	-1.29904	3.032519	-4.81315
H	3.283211	-0.29169	-4.80182
H	-3.30614	0.317376	4.945733
H	2.009352	2.687172	4.940371
H	1.394707	-3.09222	4.913233
O	-5.55837	0.795663	-3.17886
H	-4.91387	1.396665	-3.5734
H	-5.04226	0.295775	-2.50926

$Ce_6(H_2O)_6(OH)_6(\mu_3–OH)_4(\mu_3–O)_4(HCO_2)_6$ with H_2O Proximal to μ_3–O-1

C	1.122566	-2.37556	4.014748
O	-2.56959	-0.77024	3.485411
O	-0.01786	-2.57299	3.528182
O	-1.48128	-3.83096	1.444637
Ce	-1.29277	-1.641	1.626327
H	-1.17005	-4.36827	2.183163
O	3.614632	-2.23108	1.080113
O	2.651798	-3.21771	-1.25889
O	0.716684	-1.6486	0.724643
O	2.042933	-1.68006	3.504104
H	3.332252	-2.65975	0.232104
H	4.530848	-1.95945	0.943188
H	-0.51159	-4.21683	0.101105
H	3.335289	-0.43193	-1.08978
C	-2.72645	0.390618	3.952856
C	1.599892	2.332997	3.913778
O	-2.32589	1.46204	3.434558
O	-0.00104	0.082403	2.628422
O	0.180462	4.318119	0.958316
O	0.512198	2.750367	3.429557
O	-1.82513	0.269032	0.688892
Ce	-0.83264	2.032144	1.555481
H	-3.2547	3.337547	2.014828
O	3.685851	2.21289	-0.84251
O	4.112873	0.70989	1.34591
O	1.103026	1.509642	0.661781
O	2.33402	1.435841	3.432253
O	2.431486	-0.2945	-0.78269
Ce	2.122612	-0.2054	1.594023
H	4.436023	1.272076	2.060321
H	3.937375	1.71104	-0.02186
C	-1.52187	-2.19876	-3.86496
O	-3.7865	-1.94381	1.074876
O	-4.11782	-0.68102	-1.30604
O	-1.4883	-1.93749	-0.74544
O	-2.19643	-1.22192	-3.45752
H	-4.00123	-1.50347	0.21308
H	-4.00658	-2.87573	0.952437
H	-4.33131	-1.40601	-1.90706
O	2.121761	-1.34697	-3.45416
O	-0.46442	-2.64716	-3.34391
O	0.058363	-4.27973	-0.71143
Ce	0.850665	-1.97449	-1.44326
H	3.373313	-3.05672	-1.88016
H	0.793796	-4.86212	-0.48635
C	-1.10452	2.345199	-3.95632
O	-3.74638	2.1112	-0.84217
O	-2.62061	3.296697	1.288793
O	-0.95207	2.275342	-0.83152
O	-2.03113	1.667183	-3.43406
Ce	-2.13929	0.250524	-1.48763
H	0.669081	4.263928	0.097141
H	-0.50889	4.980981	0.828333
H	-3.42031	2.60353	-0.04172
H	-4.62455	1.778271	-0.62144
H	-1.29185	3.116987	-1.15732
C	2.622413	-0.28745	-3.90336
O	2.488087	0.868491	-3.41681
O	-0.00298	-0.00795	-1.86841
O	1.453661	3.91327	-1.39929
O	0.070221	2.454086	-3.52852
Ce	1.280946	1.727135	-1.51662
H	0.941735	4.435608	-2.02989
H	3.845581	3.145675	-0.65557
H	-2.0514	-2.66408	-1.03707
H	-1.88702	-2.72063	-4.77141
H	-1.35607	2.895832	-4.88415
H	3.242905	-0.37111	-4.81731
H	-3.27805	0.469894	4.91008
H	1.939504	2.811989	4.853096
H	1.348226	-2.86314	4.983322
O	0.05178	0.118802	5.393915
H	-0.54678	-0.56094	5.729129
H	-0.33207	0.95421	5.689654
H	0.003467	0.09203	3.60896

$Ce_6(H_2O)_6(OH)_6(\mu_3\text{–}OH)_5(\mu_3\text{–}O)_3(HCO_2)_6$ μ_3–O-1 with Proximal H_2O

C	1.171207	-2.43109	4.043599
O	-2.54174	-0.8516	3.541592
O	0.036983	-2.64126	3.547344
O	-1.45498	-3.85602	1.431939
Ce	-1.21402	-1.63234	1.663196
H	-1.09064	-4.38023	2.154995
O	3.632214	-2.25362	1.074852
O	2.659852	-3.23756	-1.28536
O	0.766385	-1.77112	0.715662
O	2.08542	-1.72071	3.544498
H	3.340268	-2.66356	0.222479
H	4.521478	-1.9177	0.907647
H	-0.52865	-4.22306	0.078947
H	3.331498	-0.4485	-1.0538
C	-2.6844	0.298609	4.037293
C	1.651122	2.303482	3.966321
O	-2.28643	1.379656	3.537203
O	0.030002	0.027741	2.490782
O	0.16235	4.286438	1.039382
O	0.54758	2.707834	3.510064
O	-1.90557	0.260174	0.721005
Ce	-0.78952	1.916245	1.638989
H	-3.22285	3.209371	2.11585
O	3.730201	2.169984	-0.82789
O	4.122909	0.665819	1.346654
O	1.189143	1.556605	0.688365
O	2.377405	1.404865	3.474869
O	2.420561	-0.31074	-0.77112
Ce	2.073707	-0.22222	1.630884
H	4.411896	1.238139	2.067205
H	3.955002	1.654893	-0.00197
C	-1.55323	-2.17278	-3.85478
O	-3.75057	-2.0068	1.108296
O	-4.12852	-0.67754	-1.25354
O	-1.47327	-1.92925	-0.73045
O	-2.21767	-1.19391	-3.43509
H	-3.96239	-1.54852	0.257084
H	-3.8686	-2.94793	0.92891
H	-4.35097	-1.37978	-1.87728
O	2.109125	-1.33438	-3.46624
O	-0.48841	-2.62667	-3.35517
O	0.021645	-4.30397	-0.75082
Ce	0.848818	-1.98301	-1.43962
H	3.374792	-3.07272	-1.91247
H	0.751018	-4.89784	-0.53708
C	-1.10943	2.389725	-3.8943
O	-3.72143	2.155816	-0.77789
O	-2.59079	3.240368	1.388
O	-0.92765	2.251471	-0.766
O	-2.03219	1.699641	-3.38214
Ce	-2.1385	0.259621	-1.43525
H	0.657477	4.229609	0.184101
H	-0.59051	4.864064	0.863094
H	-3.37604	2.60435	0.04505
H	-4.60997	1.845382	-0.5675
H	-1.26955	3.107701	-1.04657
C	2.614612	-0.27172	-3.90258
O	2.485161	0.880148	-3.40605
O	-0.00104	-0.00394	-1.8288
O	1.476525	3.920734	-1.32848
O	0.069759	2.487631	-3.4758
Ce	1.295639	1.724494	-1.46971
H	0.973098	4.450426	-1.95909
H	3.893406	3.098582	-0.62468
H	-2.04825	-2.65485	-0.99821
H	-1.93724	-2.69152	-4.75591
H	-1.36882	2.9641	-4.80605
H	3.236174	-0.34913	-4.81701
H	-3.22324	0.359276	5.003558
H	2.015401	2.796351	4.889602
H	1.3966	-2.92362	5.010184
O	0.065362	0.025262	5.339357
H	0.051421	0.050856	4.360801
H	-0.28948	0.879884	5.609661

$Ce_6(H_2O)_6(OH)_6(\mu_3–OH)_4(\mu_3–O)_4(HCO_2)_6$ with H_2O Proximal to μ_3–O-2

C	1.149265	-2.48587	3.9801
O	-2.56498	-0.88023	3.460623
O	0.004311	-2.66205	3.496803
O	-1.40976	-3.9039	1.386984
Ce	-1.27188	-1.71082	1.593514
H	-1.06439	-4.43121	2.117328
O	3.641043	-2.25904	1.06988
O	2.680228	-3.20244	-1.28794
O	0.750165	-1.68377	0.7214
O	2.057431	-1.74794	3.506826
H	3.360314	-2.67355	0.214096
H	4.559935	-1.99198	0.942096
H	-0.46866	-4.2613	0.034266
H	3.358765	-0.41299	-1.06856
C	-2.75032	0.269661	3.945879
C	1.597496	2.254117	3.97529
O	-2.31816	1.353477	3.481902
O	0.006129	0.007866	2.652201
O	0.167813	4.281283	1.057959
O	0.504493	2.666544	3.498298
O	-1.81437	0.224822	0.716441
Ce	-0.83567	1.981795	1.606778
H	-3.2604	3.274954	2.095964
O	3.691894	2.235655	-0.76827
O	4.122876	0.691273	1.392406
O	1.105741	1.475854	0.717932
O	2.327239	1.351837	3.496026
O	2.454415	-0.28596	-0.75876
Ce	2.145012	-0.24886	1.612545
H	4.453979	1.2246	2.12506
H	3.945197	1.723685	0.044992
C	-1.49169	-2.17692	-3.8758
O	-3.74566	-2.07985	1.011977
O	-4.08506	-0.72717	-1.31315
O	-1.44379	-1.9322	-0.76577
O	-2.16266	-1.19943	-3.46513
H	-3.96187	-1.60708	0.166764
H	-3.89998	-3.01537	0.827684
H	-4.27617	-1.44036	-1.93582
O	2.159508	-1.28998	-3.44851
O	-0.41483	-2.60534	-3.37859
O	0.053613	-4.29994	-0.81336
Ce	0.877067	-1.96197	-1.4576
H	3.401196	-3.04044	-1.90947
H	0.758996	-4.94352	-0.67915
C	-1.08929	2.386515	-3.89417
O	-3.74603	2.067788	-0.7931
O	-2.63552	3.229215	1.362356
O	-0.94634	2.264821	-0.76848
O	-2.01142	1.693195	-3.38469
Ce	-2.11619	0.236111	-1.46695
H	0.66253	4.246171	0.199231
H	-0.51679	4.951239	0.940019
H	-3.43127	2.549765	0.017414
H	-4.6241	1.725659	-0.58659
H	-1.29512	3.107328	-1.08241
C	2.653347	-0.21869	-3.87623
O	2.507788	0.927407	-3.36958
O	0.024626	0.012624	-1.85262
O	1.449739	3.929168	-1.30386
O	0.082262	2.502447	-3.45915
Ce	1.292125	1.744311	-1.45868
H	0.934204	4.461674	-1.9228
H	3.845107	3.166663	-0.56753
H	0.001692	-0.00248	3.615719
H	-1.87535	-2.7143	-4.76491
H	-1.34156	2.946147	-4.81643
H	3.278739	-0.28059	-4.78853
H	-3.35661	0.3244	4.871625
H	1.944529	2.740325	4.90854
H	1.391613	-3.03073	4.913991
O	-2.76446	-4.13765	-1.77086
H	-1.89805	-4.56754	-1.702
H	-3.28783	-4.53217	-1.06165
H	-2.01566	-2.66357	-1.07757

$Ce_6(H_2O)_6(OH)_6(\mu_3{-}OH)_5(\mu_3{-}O)_3(HCO_2)_6$ μ_3–O-2 with Proximal H_2O

C	1.154472	-2.51115	3.952263
O	-2.55722	-0.91252	3.41646
O	0.013182	-2.68052	3.463604
O	-1.45159	-3.93359	1.375993
Ce	-1.25963	-1.71937	1.489249
H	-1.07207	-4.41783	2.118916
O	3.687564	-2.24334	1.03471
O	2.68633	-3.18691	-1.28946
O	0.834672	-1.74223	0.755604
O	2.066042	-1.75864	3.502456
H	3.375302	-2.64667	0.180057
H	4.595832	-1.95949	0.87373
H	-0.474	-4.27509	0.036305
H	3.346675	-0.43402	-1.08889
C	-2.74801	0.225738	3.921415
C	1.587214	2.234991	3.994894
O	-2.30774	1.322832	3.492088
O	0.013853	-0.00406	2.61861
O	0.160645	4.286536	1.052459
O	0.504448	2.659506	3.50569
O	-1.87653	0.263041	0.746091
Ce	-0.83579	1.967957	1.601005
H	-3.28618	3.216854	2.085326
O	3.71403	2.240341	-0.76496
O	4.144029	0.71147	1.402467
O	1.111407	1.468506	0.7118
O	2.326751	1.342888	3.512038
O	2.456186	-0.27277	-0.75762
Ce	2.157695	-0.2547	1.606408
H	4.441688	1.259073	2.138642
H	3.957813	1.726916	0.0517
C	-1.47943	-2.22715	-3.89373
O	-3.76511	-2.07346	0.955897
O	-4.09683	-0.72692	-1.3611
O	-1.32298	-1.81839	-0.73459
O	-2.13364	-1.23119	-3.50181
H	-3.97051	-1.61091	0.100528
H	-3.90228	-3.01173	0.77302
H	-4.24709	-1.45998	-1.9714
O	2.167655	-1.2889	-3.46023
O	-0.40869	-2.66405	-3.39017
O	0.100085	-4.31692	-0.7761
Ce	0.828795	-1.95257	-1.43549
H	3.379356	-2.97125	-1.92557
H	0.861201	-4.86141	-0.54244
C	-1.07737	2.383408	-3.90128
O	-3.73185	2.047648	-0.81821
O	-2.63721	3.236435	1.371825
O	-0.92806	2.249191	-0.76911
O	-1.97978	1.666581	-3.39474
Ce	-2.06091	0.166963	-1.45437
H	0.663968	4.2406	0.197469
H	-0.54697	4.925895	0.904089
H	-3.41826	2.522414	-0.00634
H	-4.591	1.669587	-0.59596
H	-1.30476	3.070728	-1.1035
C	2.66254	-0.22138	-3.89007
O	2.540206	0.928286	-3.37881
O	0.071902	0.051167	-1.95057
O	1.471937	3.946056	-1.28889
O	0.088587	2.547561	-3.45978
Ce	1.305517	1.744733	-1.47031
H	0.948448	4.468757	-1.90923
H	3.85531	3.171482	-0.55678
H	-0.0154	-0.04789	3.580143
H	-1.87274	-2.77584	-4.77253
H	-1.33791	2.92649	-4.8322
H	3.272493	-0.28322	-4.81388
H	-3.36934	0.263302	4.839086
H	1.916285	2.701649	4.945088
H	1.398486	-3.07584	4.874911
O	-3.02417	-3.84719	-1.61301
H	-2.45183	-4.4154	-2.14105
H	-2.42792	-3.13878	-1.28915

$Ce_6(H_2O)_6(OH)_6(\mu_3\text{–}OH)_4(\mu_3\text{–}O)_4(HCO_2)_6$ with H_2O Proximal to Terminal –OH_2-1

C	1.161157	-2.46384	4.032255
O	-2.54316	-0.85016	3.542249
O	0.009643	-2.64091	3.564166
O	-1.39635	-3.93976	1.480893
Ce	-1.28352	-1.71244	1.674569
H	-1.09961	-4.48787	2.216078
O	3.618591	-2.28431	1.090937
O	2.652765	-3.24384	-1.2541
O	0.714809	-1.69864	0.764653
O	2.068403	-1.74085	3.535449
H	3.333748	-2.70256	0.238008
H	4.536905	-2.01831	0.956303
H	-0.45763	-4.29643	0.121559
H	3.319509	-0.46453	-1.07151
C	-2.71799	0.307859	4.012278
C	1.636163	2.280923	3.944518
O	-2.29201	1.382545	3.522544
O	0.013304	0.013018	2.688779
O	0.151017	4.26586	1.01647
O	0.537348	2.687133	3.475415
O	-1.81993	0.196044	0.766396
Ce	-0.83252	1.975046	1.620541
H	-3.24122	3.278782	2.132247
O	3.658886	2.178812	-0.81602
O	4.113339	0.664542	1.362234
O	1.092826	1.450536	0.716281
O	2.350532	1.360182	3.477462
O	2.417771	-0.33597	-0.7544
Ce	2.137507	-0.26474	1.623809
H	4.448264	1.203714	2.089003
H	3.923474	1.679652	0.001214
C	-1.56668	-2.24227	-3.80358
O	-3.76873	-2.07228	1.14972
O	-4.14741	-0.70342	-1.2123
O	-1.49977	-2.00647	-0.68665
O	-2.24532	-1.27455	-3.38088
H	-4.00422	-1.51826	0.368483
H	-4.36303	-1.46763	-1.76208
O	2.082192	-1.37209	-3.42937
O	-0.49564	-2.68044	-3.30261
O	0.081957	-4.3391	-0.71251
Ce	0.837578	-2.01995	-1.41253
H	3.371435	-3.06342	-1.87335
H	0.827487	-4.92238	-0.52608
C	-1.16167	2.297551	-3.8816
O	-3.76476	2.050693	-0.74132
O	-2.62839	3.227512	1.38885
O	-0.97647	2.216843	-0.75795
O	-2.08068	1.614868	-3.35228
Ce	-2.16567	0.191192	-1.41373
H	0.636609	4.212785	0.153437
H	-0.55598	4.908855	0.879871
H	-3.44152	2.543108	0.059353
H	-4.64066	1.711503	-0.52112
H	-1.32303	3.057658	-1.07932
C	2.571917	-0.30833	-3.87994
O	2.439929	0.843857	-3.38342
O	-0.02994	-0.05817	-1.81212
O	1.417646	3.866997	-1.34548
O	0.015316	2.412927	-3.46168
Ce	1.250782	1.682766	-1.46802
H	0.900344	4.39119	-1.97021
H	3.817334	3.112545	-0.63268
H	0.022206	0.01952	3.652406
H	-2.06857	-2.73301	-0.96902
H	-1.94142	-2.76438	-4.70578
H	-1.42217	2.846186	-4.808
H	3.179428	-0.38366	-4.803
H	-3.30727	0.378775	4.94758
H	2.002735	2.790261	4.857519
H	1.409561	-2.9935	4.973034
O	-3.62759	-4.70732	0.205538
H	-3.45955	-4.68958	-0.74467
H	-2.74762	-4.55094	0.627576
H	-3.89482	-2.9882	0.82685

$Ce_6(H_2O)_5(OH)_7(\mu_3\text{–}OH)_4(\mu_3\text{–}O)_4(HCO_2)_6^-$
Terminal –OH_2-1 with Proximal H_2O

C	1.143648	-2.4398	4.097928
O	-2.49542	-0.78723	3.679339
O	-0.01641	-2.61842	3.659037
O	-1.17923	-3.99639	1.508176
Ce	-1.34906	-1.76293	1.697537
H	-0.59616	-4.39761	2.163171
O	3.614028	-2.29852	1.110143
O	2.667028	-3.25263	-1.22856
O	0.722231	-1.7163	0.766182
O	2.051887	-1.74235	3.56313
H	3.330233	-2.71217	0.25211
H	4.526306	-2.01386	0.974249
H	-0.42968	-4.29072	0.112244
H	3.321901	-0.47564	-1.06051
C	-2.66278	0.38219	4.104578
C	1.615819	2.281806	3.940181
O	-2.29251	1.446265	3.538846
O	-0.0048	0.008317	2.685459
O	0.112976	4.261864	1.003065
O	0.525228	2.693523	3.45832
O	-1.85891	0.200908	0.774817
Ce	-0.86388	1.947036	1.608161
H	-3.28367	3.255656	2.080396
O	3.654695	2.172856	-0.79045
O	4.096167	0.655788	1.378002
O	1.079546	1.440671	0.712567
O	2.332615	1.356543	3.486033
O	2.420984	-0.34275	-0.74463
Ce	2.102581	-0.28745	1.62793
H	4.401755	1.203054	2.111283
H	3.904106	1.667704	0.030172
C	-1.52204	-2.28359	-3.82133
O	-3.49177	-2.31825	1.450252
O	-4.16221	-0.65451	-1.37706
O	-1.49011	-1.99676	-0.70065
O	-2.19051	-1.30166	-3.42212
H	-4.10764	-1.58128	1.356973
H	-4.2662	-1.60524	-1.51554
O	2.105236	-1.38703	-3.41831
O	-0.4571	-2.72526	-3.30716
O	0.065995	-4.35348	-0.76148
Ce	0.829115	-2.0275	-1.39268
H	3.376336	-3.05423	-1.85253
H	0.812782	-4.94451	-0.60951
C	-1.15403	2.28517	-3.89265
O	-3.78007	2.053285	-0.78638
O	-2.65939	3.229649	1.345527
O	-0.98644	2.204062	-0.76938
O	-2.07452	1.602543	-3.36927
Ce	-2.17218	0.163112	-1.41118
H	0.611595	4.201575	0.147227
H	-0.61611	4.872225	0.836562
H	-3.45168	2.540932	0.017128
H	-4.65186	1.708079	-0.55993
H	-1.33727	3.040513	-1.09583
C	2.599729	-0.32346	-3.86231
O	2.460507	0.831084	-3.37184
O	-0.019	-0.0628	-1.82616
O	1.411314	3.863654	-1.33894
O	0.022373	2.403519	-3.46832
Ce	1.244532	1.6664	-1.46266
H	0.895309	4.379401	-1.97132
H	3.800371	3.106103	-0.59536
H	0.005195	0.018258	3.648408
H	-2.10385	-2.70609	-0.96936
H	-1.89607	-2.82004	-4.71627
H	-1.40962	2.834516	-4.82068
H	3.221019	-0.39919	-4.77679
H	-3.19194	0.493201	5.072722
H	1.974963	2.792736	4.856086
H	1.407088	-2.94172	5.050912
O	-3.66157	-3.7258	-0.84389
H	-3.67572	-3.28807	0.046724
H	-3.39434	-4.63635	-0.67415

$Ce_6(H_2O)_6(OH)_6(\mu_3–OH)_4(\mu_3–O)_4(HCO_2)_6$ with H_2O Proximal to Terminal $–OH_2$-2

C	1.169398	-2.48355	3.928499
O	-2.56328	-0.81284	3.519748
O	0.008504	-2.63404	3.475178
O	-1.56074	-3.81304	1.444425
Ce	-1.30634	-1.6426	1.635109
H	-1.19274	-4.36561	2.14492
O	3.762774	-2.02048	1.102814
O	2.724746	-3.14325	-1.13482
O	0.730838	-1.61934	0.754254
O	2.05487	-1.70467	3.48
H	3.4649	-2.47312	0.273939
H	4.631063	-1.65104	0.897856
H	-0.58788	-4.16884	0.049599
H	3.335187	-0.34059	-1.0379
C	-2.76072	0.345008	3.982207
C	1.528523	2.348664	3.994614
O	-2.35852	1.423991	3.482472
O	-0.02218	0.08418	2.671622
O	0.094941	4.346413	1.020276
O	0.45043	2.763458	3.486695
O	-1.83709	0.273296	0.721694
Ce	-0.8809	2.048936	1.601747
H	-3.31495	3.325152	2.079597
O	3.646451	2.309624	-0.79421
O	4.053828	0.88492	1.438165
O	1.062589	1.551133	0.722507
O	2.261778	1.436105	3.540547
O	2.430406	-0.2151	-0.72828
Ce	2.117034	-0.14504	1.639938
H	4.310582	1.479152	2.153642
H	3.889571	1.838048	0.047505
C	-1.45782	-2.15365	-3.85168
O	-3.79751	-1.91903	1.075987
O	-4.10711	-0.69062	-1.3213
O	-1.47444	-1.91194	-0.73279
O	-2.15352	-1.1913	-3.44666
H	-4.01188	-1.49408	0.206514
H	-4.0246	-2.85165	0.973362
H	-4.29963	-1.41934	-1.92499
O	2.166669	-1.29614	-3.37681
O	-0.40563	-2.59457	-3.31311
O	0.056303	-4.25078	-0.69785
Ce	0.876501	-1.92862	-1.39372
H	3.42348	-3.12307	-1.79882
C	-1.12355	2.386098	-3.91317
O	-3.78575	2.087791	-0.80157
O	-2.68805	3.276716	1.347777
O	-0.99083	2.301641	-0.78546
O	-2.04609	1.696158	-3.39924
Ce	-2.14185	0.269135	-1.46453
H	0.591953	4.304085	0.163185
H	-0.60079	5.002308	0.888232
H	-3.47695	2.581475	0.003493
H	-4.66005	1.737475	-0.59251
H	-1.33815	3.142094	-1.10687
C	2.639494	-0.2263	-3.83349
O	2.470048	0.929745	-3.3599
O	0.00151	0.031336	-1.82014
O	1.38756	3.971383	-1.33304
O	0.047407	2.503829	-3.4777
Ce	1.253293	1.785492	-1.46377
H	0.872976	4.492255	-1.96268
H	3.796656	3.249569	-0.63758
H	-0.01656	0.069073	3.635266
H	-2.03152	-2.63627	-1.04151
H	-1.7959	-2.66982	-4.77172
H	-1.37532	2.939485	-4.83908
H	3.267927	-0.30222	-4.74254
H	-3.34866	0.410363	4.918807
H	1.856526	2.841027	4.931275
H	1.447098	-3.09125	4.812161
O	2.320121	-5.01851	0.841322
H	2.660456	-4.38681	0.172798
H	2.146339	-4.469	1.617318
H	0.800859	-4.73932	-0.30241

$Ce_6(H_2O)_5(OH)_7(\mu_3\text{–}OH)_4(\mu_3\text{–}O)_4(HCO_2)_6^-$
Terminal –OH_2-2 with Proximal H_2O

C	1.108267	-2.4791	4.02153
O	-2.59434	-0.83413	3.529197
O	-0.03286	-2.66419	3.53382
O	-1.68065	-3.84097	1.551167
Ce	-1.33769	-1.69953	1.637754
H	-1.10428	-4.41036	2.075342
O	3.60586	-2.27113	1.109017
O	2.845535	-3.08563	-1.3269
O	0.681761	-1.69451	0.766759
O	2.017423	-1.74246	3.547549
H	3.383346	-2.62765	0.20458
H	4.525617	-1.98307	1.056558
H	-0.51748	-4.20485	-0.23753
H	3.303625	-0.46582	-1.04433
C	-2.76652	0.324862	3.995547
C	1.576745	2.27378	3.972952
O	-2.34488	1.399612	3.500374
O	-0.03405	0.020437	2.682664
O	0.176891	4.272356	1.015496
O	0.488458	2.694415	3.490388
O	-1.85513	0.241567	0.740755
Ce	-0.85754	1.987814	1.607083
H	-3.25727	3.319812	2.10569
O	3.660371	2.176962	-0.77459
O	4.078644	0.671442	1.413304
O	1.069693	1.45974	0.726246
O	2.29487	1.356814	3.50652
O	2.400702	-0.327	-0.73729
Ce	2.087984	-0.27246	1.624871
H	4.358321	1.240114	2.14078
H	3.902869	1.67479	0.048527
C	-1.56795	-2.12681	-3.8972
O	-3.85017	-1.93732	1.093693
O	-4.16456	-0.66245	-1.27468
O	-1.54571	-1.94846	-0.72871
O	-2.26757	-1.18482	-3.44462
H	-4.0527	-1.49077	0.230889
H	-4.07225	-2.86784	0.965049
H	-4.36153	-1.38926	-1.87898
O	2.068868	-1.34699	-3.47456
O	-0.51216	-2.58474	-3.39162
O	0.207758	-4.13901	-0.87743
Ce	0.828539	-2.09248	-1.41701
H	3.526443	-2.68907	-1.88316
C	-1.12843	2.361494	-3.90127
O	-3.7724	2.098412	-0.7794
O	-2.64156	3.271053	1.364596
O	-0.97458	2.254893	-0.77412
O	-2.06175	1.689064	-3.38497
Ce	-2.15016	0.234048	-1.45032
H	0.669715	4.20244	0.155863
H	-0.50988	4.934847	0.871549
H	-3.44642	2.584323	0.023399
H	-4.63854	1.740138	-0.55148
H	-1.30317	3.101691	-1.09698
C	2.592751	-0.28943	-3.88985
O	2.481478	0.859219	-3.37239
O	-0.03874	-0.03102	-1.8214
O	1.449199	3.864049	-1.33426
O	0.050643	2.445231	-3.4791
Ce	1.249615	1.661566	-1.46845
H	0.911805	4.373735	-1.9537
H	3.780375	3.112236	-0.57147
H	-0.03522	0.020289	3.6459
H	-2.10349	-2.67429	-1.0307
H	-1.91712	-2.60067	-4.83656
H	-1.377	2.931985	-4.81818
H	3.216444	-0.35549	-4.80406
H	-3.35038	0.399051	4.934614
H	1.928437	2.768388	4.900382
H	1.34795	-3.01473	4.961859
O	2.347895	-5.46669	0.150303
H	2.9557	-4.81805	-0.23865
H	1.486949	-5.10237	-0.16279

$Ce_6(H_3BO_3)(H_2O)_5(OH)_6(\mu_3–OH)_4(\mu_3–O)_4(HCO_2)_6$ Terminal –OH_2-1

C	1.152851	-2.69195	3.969131
O	-2.54958	-1.0999	3.387065
O	0.019912	-2.882	3.462108
O	-1.39117	-4.13318	1.337991
Ce	-1.23211	-1.95259	1.549704
H	-1.13492	-4.68589	2.086283
O	3.705237	-2.46533	1.118237
O	2.816821	-3.43387	-1.25735
O	0.802785	-1.90954	0.699794
O	2.064269	-1.95032	3.509014
H	3.464046	-2.88202	0.251956
H	4.627305	-2.19286	1.030886
H	-0.36078	-4.49952	0.029349
H	3.449489	-0.63778	-1.04961
C	-2.76557	0.061719	3.828781
C	1.574939	2.061029	3.929635
O	-2.34153	1.136928	3.336739
O	0.022016	-0.21006	2.607669
O	0.189261	4.046713	0.96106
O	0.489623	2.462594	3.424384
O	-1.74279	-0.03699	0.618485
Ce	-0.80158	1.748321	1.520377
H	-3.22636	3.103767	1.895549
O	3.745394	2.009409	-0.77436
O	4.14735	0.493255	1.412428
O	1.145929	1.245628	0.671294
O	2.31654	1.155798	3.476455
O	2.536762	-0.52277	-0.75979
Ce	2.180376	-0.46691	1.606443
H	4.457883	1.033717	2.149012
H	3.98407	1.517884	0.055601
C	-1.28074	-2.43665	-3.96324
O	-3.71258	-2.24797	0.924724
O	-3.99252	-1.07591	-1.53647
O	-1.36167	-2.22727	-0.82862
O	-1.97462	-1.47102	-3.56302
H	-3.91773	-1.86278	0.037701
H	-3.95654	-3.18018	0.867597
H	-4.07428	-1.82452	-2.14027
O	2.301306	-1.54969	-3.44208
O	-0.24758	-2.89693	-3.40435
O	0.222602	-4.5407	-0.77395
Ce	1.003041	-2.22559	-1.46763
H	3.547133	-3.26171	-1.86539
H	0.950682	-5.13487	-0.55615
C	-1.02071	2.107577	-3.95541
O	-2.60138	2.994749	1.168615
O	-0.8832	1.994932	-0.85835
O	-1.95068	1.423819	-3.44193
Ce	-2.01097	-0.04774	-1.55521
H	0.682267	4.012541	0.102034
H	-0.51503	4.694577	0.834088
H	-1.24255	2.830862	-1.17808
C	2.783882	-0.47712	-3.87828
O	2.61524	0.672549	-3.38601
O	0.118864	-0.26401	-1.89239
O	1.498944	3.674774	-1.38579
O	0.155224	2.199991	-3.53054
Ce	1.369502	1.492747	-1.5053
H	0.989521	4.192037	-2.02279
H	3.899741	2.944943	-0.59676
H	0.00327	-0.20861	3.57127
H	-1.90251	-2.96054	-1.14537
H	-1.60253	-2.93711	-4.89752
H	-1.27444	2.676967	-4.8708
H	3.419641	-0.54004	-4.783
H	-3.39232	0.136926	4.738969
H	1.899548	2.560896	4.863394
H	1.379255	-3.22691	4.912438
O	-5.70336	0.946683	-1.86301
B	-5.02302	1.905149	-1.17118
O	-3.72321	1.666552	-0.76947
O	-5.58909	3.119097	-0.86307
H	-5.18701	0.100569	-1.85408
H	-3.36015	2.278059	-0.05673
H	-6.50322	3.196097	-1.16162

$Ce_6(H_3BO_3)(H_2O)_5(OH)_6(\mu_3\text{–}OH)_4(\mu_3\text{–}O)_4(HCO_2)_6$ Terminal –OH_2-2

C	1.354555	-2.77805	3.942485
O	-2.45236	-1.36167	3.365875
O	0.225949	-3.01002	3.443164
O	-1.06668	-4.30956	1.306888
Ce	-1.06634	-2.11286	1.541016
H	-0.68144	-4.8203	2.02912
O	3.84616	-2.40155	1.057224
O	2.981972	-3.33746	-1.33517
O	0.930899	-1.95661	0.675292
O	2.22848	-1.99288	3.482701
H	3.612035	-2.80136	0.180471
H	4.759993	-2.10173	0.974487
H	-0.11613	-4.58497	-0.05132
H	3.50304	-0.54016	-1.06593
C	-2.73318	-0.23057	3.850573
C	1.551732	1.966044	3.993054
O	-2.33229	0.880023	3.42742
O	0.095229	-0.34137	2.630516
O	0.047109	3.947326	1.086753
O	0.441664	2.323142	3.51052
O	-1.71626	-0.20588	0.678289
Ce	-0.83704	1.597806	1.594962
H	-3.32172	2.748314	2.079678
O	3.70234	2.107523	-0.73575
O	4.183687	0.568878	1.419967
O	1.140126	1.204914	0.722203
O	2.333328	1.110596	3.509775
O	2.59032	-0.46689	-0.76313
Ce	2.258474	-0.4694	1.608886
H	4.486393	1.105029	2.162901
H	3.971865	1.608836	0.079988
C	-1.22049	-2.49287	-3.95257
O	-3.98619	-1.06336	-1.44608
O	-1.27377	-2.33725	-0.82012
O	-1.95502	-1.57384	-3.51744
H	-4.08221	-1.98277	-1.72448
O	2.357776	-1.44854	-3.47484
O	-0.14309	-2.89669	-3.43478
O	0.433415	-4.57477	-0.88218
Ce	1.110883	-2.21209	-1.50585
H	3.697936	-3.1318	-1.94954
H	1.18448	-5.15907	-0.72461
C	-1.04844	2.063308	-3.9008
O	-3.70476	1.61917	-0.84249
O	-2.68427	2.770632	1.355647
O	-0.93775	1.901788	-0.77646
O	-1.94138	1.3168	-3.41544
Ce	-2.00135	-0.15479	-1.51057
H	0.541312	3.953834	0.226861
H	-0.67698	4.576567	0.980088
H	-3.42813	2.081638	-0.00726
H	-4.58447	1.259077	-0.67858
H	-1.31886	2.734215	-1.08019
C	2.805354	-0.34936	-3.88301
O	2.606574	0.779492	-3.35694
O	0.152169	-0.29007	-1.86241
O	1.372551	3.691162	-1.25719
O	0.111154	2.225283	-3.44877
Ce	1.336314	1.507686	-1.44883
H	0.86288	4.212695	-1.89016
H	3.819148	3.044456	-0.53818
H	0.079129	-0.35759	3.594002
H	-1.76238	-3.09725	-1.15809
H	-1.54512	-3.00207	-4.88124
H	-1.3167	2.62822	-4.81508
H	3.435983	-0.36853	-4.79338
H	-3.39835	-0.22382	4.736196
H	1.862444	2.45883	4.935388
H	1.611811	-3.31427	4.87703
O	-4.96622	-1.00863	1.037004
B	-4.62954	-2.14844	1.692463
O	-3.56483	-2.92161	1.224163
O	-5.30698	-2.59119	2.799189
H	-4.55897	-0.96443	0.130649
H	-3.52379	-3.79629	1.634974
H	-6.06191	-2.0354	3.02768

$Ce_6(H_2BO_3)(H_2O)_6(OH)_5(\mu_3–OH)_4(\mu_3–O)_4(HCO_2)_6$ Terminal –OH-1

C	0.915159	-2.72216	3.879729
O	-2.64041	-0.79988	3.389165
O	-0.24182	-2.78445	3.396492
O	-1.62323	-3.8992	1.232647
Ce	-1.41658	-1.70613	1.503052
H	-1.25186	-4.44323	1.93753
O	3.428776	-2.63567	0.980246
O	2.437904	-3.41002	-1.41957
O	0.588615	-1.80132	0.618592
O	1.886434	-2.06421	3.414893
H	3.1418	-2.98914	0.099218
H	4.373429	-2.45548	0.896685
H	-0.76116	-4.25767	-0.14054
H	3.324946	-0.71254	-1.10723
C	-2.72685	0.347893	3.903758
C	1.752239	1.935639	3.999263
O	-2.20581	1.402641	3.46354
O	-0.00945	-0.12595	2.603028
O	0.521424	4.154672	1.138183
O	0.702055	2.449983	3.52561
O	-1.80271	0.282752	0.66566
Ce	-0.67857	1.942494	1.61201
H	-2.98852	3.410534	2.129097
O	3.865567	1.886799	-0.74247
O	4.15558	0.262295	1.376325
O	1.222058	1.302436	0.719403
O	2.40889	0.988984	3.499983
O	2.430012	-0.52095	-0.80284
Ce	2.108985	-0.52049	1.569085
H	4.542403	0.73058	2.125872
H	4.073352	1.344976	0.064696
C	-1.60478	-1.9665	-4.02877
O	-3.89313	-2.28146	1.198403
O	-1.64875	-1.86213	-0.87514
O	-2.22132	-0.96604	-3.58551
H	-4.54918	-1.98644	0.54063
H	-3.86413	-3.24441	1.115271
O	2.073824	-1.41941	-3.52047
O	-0.59089	-2.50703	-3.51246
O	-0.24071	-4.30656	-0.99149
Ce	0.728995	-2.04114	-1.56237
H	3.17387	-3.26384	-2.02753
H	0.437589	-4.9804	-0.86447
C	-0.85709	2.510484	-3.87465
O	-3.51763	2.398533	-0.82946
O	-2.35834	3.343449	1.401494
O	-0.7482	2.292716	-0.75421
O	-1.83734	1.883171	-3.38834
Ce	-2.08466	0.406609	-1.51273
H	1.003435	4.112028	0.272712
H	-0.0937	4.89447	1.062998
H	-3.19061	2.799177	0.018796
H	-4.43419	2.138564	-0.67873
H	-1.01713	3.173015	-1.04226
C	2.659304	-0.38193	-3.91475
O	2.602095	0.758076	-3.37788
O	0.02755	-0.00336	-1.88462
O	1.774553	3.767726	-1.22976
O	0.316583	2.516026	-3.43111
Ce	1.445975	1.612373	-1.44898
H	1.322959	4.36264	-1.84173
H	4.095144	2.799764	-0.53185
H	-0.02582	-0.15427	3.566262
H	-2.22237	-2.55811	-1.21736
H	-1.98707	-2.42094	-4.96375
H	-1.0558	3.11274	-4.78263
H	3.287252	-0.47118	-4.82281
H	-3.32238	0.431268	4.834163
H	2.129127	2.365299	4.948268
H	1.106849	-3.30054	4.804972
O	-5.79973	-1.79935	-0.86306
B	-4.93032	-1.3085	-1.83511
O	-4.8994	-1.95219	-3.05836
O	-4.15768	-0.24906	-1.5238
H	-6.29046	-2.58179	-1.14238
H	-4.18088	-1.58938	-3.59966

$Ce_6(H_2BO_3)(H_2O)_6(OH)_5(\mu_3–OH)_4(\mu_3–O)_4(HCO_2)_6$ Terminal –OH-2

C	1.347315	-2.82296	3.959754
O	-2.38641	-1.3026	3.650871
O	0.191464	-3.01293	3.507939
O	-1.26803	-4.20679	1.401436
Ce	-1.17187	-2.0281	1.693741
H	-0.90479	-4.76964	2.095803
O	3.726511	-2.44121	0.967068
O	2.725835	-3.31159	-1.39717
O	0.809309	-1.91083	0.738421
O	2.221551	-2.04998	3.479314
H	3.436385	-2.8254	0.10007
H	4.638448	-2.15345	0.83454
H	-0.35375	-4.48982	-0.00068
H	3.333873	-0.52477	-1.09657
C	-2.61172	-0.16437	4.145382
C	1.673417	1.898521	4.118173
O	-2.25039	0.940126	3.669259
O	0.101712	-0.31861	2.751071
O	0.350974	3.937976	1.358777
O	0.538205	2.275317	3.716982
O	-1.80846	-0.06401	0.889729
Ce	-0.81038	1.67363	1.806935
O	3.610655	2.115434	-0.71119
O	4.155367	0.510749	1.377999
O	1.098949	1.232434	0.832982
O	2.417781	1.053509	3.562609
O	2.437723	-0.43552	-0.75129
Ce	2.212715	-0.48852	1.633478
H	4.507033	1.018716	2.118999
H	3.908619	1.583001	0.073141
C	-1.5844	-2.30049	-3.78765
O	-3.65321	-2.40055	1.236598
O	-4.08935	-1.03487	-1.0133
O	-1.42154	-2.19542	-0.67384
O	-2.28019	-1.37714	-3.29855
H	-3.90781	-1.92672	0.398471
H	-3.85283	-3.3325	1.084044
H	-4.37136	-1.66789	-1.68533
O	2.060649	-1.34712	-3.46552
O	-0.48185	-2.72125	-3.34356
O	0.180076	-4.48218	-0.83967
Ce	0.882043	-2.12042	-1.45408
H	3.419095	-3.09152	-2.03241
H	0.925156	-5.0784	-0.69924
C	-1.23651	2.241973	-3.68786
O	-3.92655	1.77295	-0.89625
O	-1.02397	2.043244	-0.54108
O	-2.11498	1.490206	-3.18884
Ce	-2.17543	0.010781	-1.27834
H	0.725647	4.014636	0.445189
H	-0.02586	4.79464	1.587554
H	-3.67981	2.690263	-0.66123
H	-4.70808	1.545396	-0.37954
H	-1.42544	2.887758	-0.79038
C	2.522183	-0.25199	-3.86892
O	2.377459	0.870472	-3.31246
O	-0.04423	-0.15838	-1.73957
O	1.338715	3.772426	-1.136
O	-0.06544	2.401984	-3.26381
Ce	1.20272	1.588184	-1.33702
H	0.742045	4.294253	-1.6875
H	3.746562	3.043057	-0.48399
H	0.125035	-0.35214	3.713779
H	-1.98926	-2.90909	-0.98808
H	-1.97341	-2.79528	-4.69919
H	-1.52732	2.815838	-4.58982
H	3.113865	-0.26856	-4.80522
H	-3.18781	-0.14047	5.091337
H	2.046053	2.36146	5.053141
H	1.632556	-3.3889	4.868539
O	-2.91276	4.228826	-0.21014
B	-2.66844	4.295137	1.165124
O	-2.68458	5.558918	1.730094
O	-2.42596	3.162915	1.848237
H	-3.09659	5.081029	-0.6224
H	-2.49065	5.532176	2.674937

$Ce_6(\mu_2\text{-}HBO_3)(H_2O)_6(OH)_4(\mu_3\text{–}OH)_4(\mu_3\text{–}O)_4(HCO_2)_6$
Terminal –OH-1 and –OH-2

C	1.11036	-2.40388	4.062586
O	-2.58247	-0.80815	3.51541
O	-0.03587	-2.58668	3.581353
Ce	-1.28402	-1.69794	1.659606
O	3.679493	-2.23273	1.253239
O	3.013954	-3.21899	-1.27226
O	0.688876	-1.62079	0.770214
O	2.020396	-1.68173	3.572451
H	3.551857	-2.65324	0.38015
H	4.609264	-1.97485	1.286868
H	3.311163	-0.48552	-1.07811
C	-2.75679	0.351117	3.979706
C	1.609362	2.323135	3.956094
O	-2.32022	1.424456	3.493696
O	-0.00741	0.060803	2.684725
O	0.213598	4.283463	1.006139
O	0.513792	2.728812	3.48014
O	-1.83414	0.255717	0.753138
Ce	-0.83917	2.012518	1.609208
H	-3.22492	3.368032	2.117692
O	3.682348	2.149775	-0.84842
O	4.103051	0.721796	1.375112
O	1.101216	1.473619	0.708135
O	2.333856	1.411634	3.485799
O	2.416023	-0.37298	-0.73748
Ce	2.128035	-0.21808	1.638851
H	4.424265	1.293729	2.082673
H	3.92757	1.682663	-0.00472
C	-1.63015	-2.12089	-3.83319
O	-3.75549	-2.08409	1.125626
O	-4.16228	-0.66871	-1.16349
O	-1.5441	-1.89469	-0.7289
O	-2.3065	-1.15724	-3.39866
H	-3.98792	-1.60389	0.288591
H	-3.96482	-3.0119	0.959041
H	-4.42112	-1.34648	-1.80068
O	2.081179	-1.44043	-3.39941
O	-0.57138	-2.58073	-3.32543
Ce	0.740519	-2.01312	-1.3976
H	3.686965	-2.9309	-1.90388
C	-1.12977	2.351863	-3.88977
O	-3.76355	2.161502	-0.75367
O	-2.60963	3.305419	1.377191
O	-0.96218	2.268764	-0.76614
O	-2.06973	1.702942	-3.35601
Ce	-2.19442	0.278055	-1.40888
H	0.683387	4.222513	0.135297
H	-0.4572	4.968619	0.896751
H	-3.4351	2.637768	0.055747
H	-4.65933	1.86406	-0.55453
H	-1.28483	3.117754	-1.09038
C	2.585888	-0.38524	-3.85342
O	2.442	0.77666	-3.38288
O	-0.0461	-0.02363	-1.81497
O	1.457185	3.858548	-1.36751
O	0.053595	2.424011	-3.47688
Ce	1.266063	1.672232	-1.47798
H	0.93894	4.378337	-1.99503
H	3.845567	3.089109	-0.70107
H	-0.01452	0.069609	3.648251
H	-2.10217	-2.63419	-0.99496
H	-1.99461	-2.61927	-4.75286
H	-1.37511	2.911013	-4.8142
H	3.220331	-0.47733	-4.75703
H	-3.35774	0.42839	4.907474
H	1.964351	2.823791	4.878445
H	1.347088	-2.92734	5.009972
O	0.296812	-4.02648	-0.71224
O	-0.53238	-5.96232	0.329915
B	-0.47172	-4.56117	0.288113
O	-1.16869	-3.8367	1.215383
H	-1.09206	-6.26806	1.052885
H	2.985544	-4.18349	-1.32693

$Ce_6(\mu_2\text{-}H_2BO_3)(H_2O)_5(OH)_5(\mu_3\text{–}OH)_4(\mu_3\text{–}O)_4(HCO_2)_6$ Terminal –OH-2 and –OH_2-1

C	0.990862	-2.75051	3.851479
O	-2.6036	-0.87028	3.383937
O	-0.16226	-2.82634	3.359884
O	-1.71461	-3.87586	1.232894
Ce	-1.3604	-1.72466	1.50449
H	-1.46197	-4.45888	1.959317
O	3.508002	-2.59878	0.968528
O	2.497952	-3.40567	-1.42137
O	0.653668	-1.8099	0.627576
O	1.951021	-2.06355	3.40673
H	3.21714	-2.96347	0.094284
H	4.446537	-2.39564	0.869397
H	-0.73648	-4.28377	-0.12179
H	3.360577	-0.68414	-1.11837
C	-2.71615	0.281172	3.88968
C	1.773337	1.937829	4.011481
O	-2.20391	1.339762	3.451735
O	0.035315	-0.13559	2.613992
O	0.522425	4.155217	1.140188
O	0.719712	2.447998	3.539508
O	-1.72048	0.267367	0.661894
Ce	-0.6508	1.933079	1.622439
H	-2.98567	3.358837	2.137094
O	3.874858	1.917806	-0.75496
O	4.18795	0.30203	1.372102
O	1.246926	1.310203	0.71969
O	2.44051	1.003443	3.50382
O	2.464234	-0.50657	-0.80961
Ce	2.152741	-0.50829	1.566291
H	4.563238	0.781587	2.120455
H	4.089473	1.380381	0.052956
C	-1.62609	-1.99441	-3.96621
O	-1.55221	-1.90141	-0.85843
O	-2.2406	-1.00239	-3.50054
O	2.09281	-1.41522	-3.52809
O	-0.58599	-2.52053	-3.48672
O	-0.16261	-4.32729	-0.93041
Ce	0.780188	-2.05075	-1.55638
H	3.225736	-3.26467	-2.04038
H	0.511767	-4.99489	-0.7578
C	-0.87193	2.491337	-3.86786
O	-3.53474	2.321785	-0.809
O	-2.36031	3.300538	1.40449
O	-0.74403	2.281527	-0.74982
O	-1.84506	1.854847	-3.37998
Ce	-2.05492	0.367098	-1.48854
H	0.999398	4.113908	0.271376
H	-0.09931	4.889778	1.067313
H	-3.19662	2.732541	0.030888
H	-4.43987	2.040683	-0.62823
H	-1.01919	3.158836	-1.04045
C	2.668071	-0.37242	-3.92513
O	2.606429	0.765971	-3.38623
O	0.053329	-0.01307	-1.87715
O	1.763392	3.776273	-1.23424
O	0.304118	2.505145	-3.42909
Ce	1.452771	1.616038	-1.45178
H	1.298779	4.363564	-1.84387
H	4.089449	2.834406	-0.54413
H	0.026484	-0.16879	3.577043
H	-2.15686	-2.57307	-1.19498
H	-2.03604	-2.45213	-4.88769
H	-1.07837	3.095644	-4.77307
H	3.289955	-0.4563	-4.83785
H	-3.32854	0.359562	4.809296
H	2.142611	2.360673	4.966579
H	1.188234	-3.34478	4.765398
O	-3.88541	-1.73617	0.903575
B	-4.64171	-1.19958	-0.15341
O	-5.99839	-1.4699	-0.08857
O	-4.06233	-0.4909	-1.13044
H	-4.42845	-2.20256	1.551296
H	-6.47967	-1.0793	-0.82754

$Ce_6(Tris)(H_2O)_5(OH)_6(\mu_3{-}OH)_4(\mu_3{-}O)_4(HCO_2)_6$
Terminal –OH_2-1

C	1.116705	-2.66196	3.877168
O	-2.52264	-1.26293	3.437137
O	0.001427	-2.89557	3.353607
O	-1.27762	-4.14985	1.186166
Ce	-1.32089	-1.96059	1.459965
H	-0.90332	-4.66285	1.912664
O	3.663975	-2.19361	1.066157
O	2.86527	-3.11004	-1.34688
O	0.729947	-1.77437	0.630112
O	1.984041	-1.84309	3.462184
H	3.454945	-2.58842	0.179884
H	4.571553	-1.87103	1.003051
H	-0.26328	-4.39784	-0.13477
H	3.339783	-0.30058	-1.02732
C	-2.78609	-0.13952	3.945436
C	1.268804	2.120633	3.994883
O	-2.52467	0.981119	3.443119
O	-0.13721	-0.19141	2.580379
O	-0.28743	4.121893	1.091922
O	0.170483	2.477508	3.487028
O	-1.9367	-0.06352	0.598262
Ce	-1.08971	1.734235	1.558632
H	-3.64072	2.737246	2.036967
O	3.497536	2.33906	-0.65519
O	3.947225	0.77626	1.483772
O	0.909986	1.392506	0.723425
O	2.060929	1.263285	3.532024
O	2.419588	-0.24434	-0.74439
Ce	2.025763	-0.29722	1.608421
H	4.188402	1.319247	2.244185
H	3.750181	1.826344	0.158639
C	-1.18154	-2.2459	-4.11355
O	-4.05104	-0.96998	-1.86431
O	-1.41564	-2.16156	-0.9401
O	-1.93255	-1.32909	-3.70948
H	-4.11863	-1.71566	-2.47352
O	2.27438	-1.18377	-3.46802
O	-0.17063	-2.70542	-3.51119
O	0.316879	-4.38064	-0.94385
Ce	0.980834	-1.99872	-1.53182
H	3.58756	-2.88327	-1.94601
H	1.068114	-4.9563	-0.75832
C	-1.17937	2.309843	-3.93
O	-3.91836	1.703141	-0.93076
O	-2.99115	2.818782	1.328132
O	-1.14726	2.078121	-0.80955
O	-2.07583	1.54445	-3.4825
Ce	-2.15348	0.013201	-1.62655
H	0.245399	4.14475	0.255058
H	-1.03166	4.7192	0.94641
H	-3.68195	2.151161	-0.07552
H	-4.79965	1.328498	-0.81638
H	-1.53266	2.911538	-1.10441
C	2.709353	-0.07234	-3.85369
O	2.480503	1.047727	-3.31929
O	0.013164	-0.07691	-1.90886
O	1.147632	3.911207	-1.19211
O	-0.02997	2.469413	-3.45155
Ce	1.151556	1.727491	-1.43174
H	0.655141	4.436304	-1.83555
H	3.602251	3.272791	-0.43706
H	-0.15234	-0.22614	3.542933
H	-1.8548	-2.92151	-1.33999
H	-1.42202	-2.70421	-5.09328
H	-1.43373	2.896473	-4.83466
H	3.357998	-0.06843	-4.75182
H	-3.30075	-0.1468	4.926086
H	1.56023	2.615815	4.942353
H	1.372575	-3.22832	4.794589
C	-5.12127	-2.45539	1.37847
C	-6.19559	-3.06422	0.453211
C	-5.05193	-3.21404	2.713813
C	-5.35219	-0.94515	1.560053
N	-3.80953	-2.61182	0.719466
H	-5.91538	-4.10197	0.223869
H	-6.21799	-2.49919	-0.49372
O	-7.49734	-3.11279	1.031664
H	-4.17845	-2.87149	3.276649
H	-4.92882	-4.29024	2.503575
O	-6.18468	-3.00172	3.552695
H	-4.59003	-0.52855	2.227942
H	-5.25173	-0.45339	0.584678
O	-6.6575	-0.62749	2.037816
H	-3.87689	-2.18252	-0.20748

H	-3.64263	-3.60482	0.556839
H	-6.96537	-3.30671	3.068449
H	-7.73452	-2.20253	1.265213
H	-6.75337	-1.03828	2.908839

$Ce_6(Tris)(H_2O)_5(OH)_6(\mu_3–OH)_4(\mu_3–O)_4(HCO_2)_6$ Terminal $–OH_2$-2

C	0.859064	-2.67647	3.936173
O	-2.7358	-0.78194	3.489567
O	-0.2947	-2.77341	3.455706
O	-2.10824	-3.78082	1.595297
Ce	-1.56068	-1.69472	1.582741
H	-1.6787	-4.37916	2.218682
O	3.287902	-2.64666	0.999553
O	2.380928	-3.36445	-1.43182
O	0.463067	-1.84365	0.666675
O	1.816482	-2.00319	3.460454
H	3.034066	-2.97568	0.097234
H	4.240297	-2.49421	0.969092
H	3.160235	-0.73403	-1.11506
C	-2.82194	0.379284	3.974481
C	1.658727	1.992311	3.96048
O	-2.3186	1.425648	3.496284
O	-0.12283	-0.10455	2.634051
O	0.370949	4.160449	1.085596
O	0.596006	2.494274	3.500835
O	-1.92463	0.281759	0.702878
Ce	-0.81148	1.948476	1.612534
H	-3.12686	3.413123	2.136244
O	3.69671	1.868988	-0.78546
O	4.019951	0.260253	1.344283
O	1.0725	1.296747	0.705788
O	2.309303	1.040141	3.46472
O	2.268546	-0.54045	-0.80336
Ce	1.972341	-0.52142	1.567231
H	4.389707	0.758431	2.083206
H	3.915167	1.331564	0.021596
C	-1.78061	-2.0178	-3.95874
O	-4.06999	-1.72313	1.030918
O	-4.28859	-0.44423	-1.33699
O	-1.76023	-1.92902	-0.79648
O	-2.42322	-1.04716	-3.49175
H	-4.24871	-1.2844	0.159832
H	-4.3797	-2.6325	0.93806
H	-4.5421	-1.13712	-1.95993
O	1.894943	-1.46349	-3.51746
O	-0.74413	-2.53567	-3.46189
Ce	0.575831	-2.10187	-1.51138
H	3.095394	-3.09925	-2.02398
C	-1.07579	2.439662	-3.87658
O	-3.71605	2.2915	-0.768
O	-2.51298	3.33107	1.396471
O	-0.91837	2.266488	-0.75931
O	-2.04638	1.811647	-3.37215
Ce	-2.24378	0.344322	-1.47238
H	0.845745	4.100538	0.216535
H	-0.24918	4.894757	0.998933
H	-3.36523	2.733304	0.050554
H	-4.62354	2.028405	-0.57385
H	-1.19521	3.13954	-1.06087
C	2.469065	-0.42782	-3.9261
O	2.412987	0.718137	-3.39796
O	-0.14056	-0.05522	-1.85973
O	1.599534	3.736946	-1.28714
O	0.104943	2.450095	-3.45181
Ce	1.266391	1.572212	-1.46959
H	1.129013	4.314422	-1.90154
H	3.91706	2.784951	-0.57827
H	-0.11635	-0.11667	3.597533
H	-2.41063	-2.56604	-1.11358
H	-2.15657	-2.4663	-4.89905
H	-1.29055	3.038375	-4.78358
H	3.087152	-0.51836	-4.84107
H	-3.40062	0.482839	4.913525
H	2.053876	2.440891	4.893448
H	1.067412	-3.23538	4.870223
C	-0.64074	-5.65371	-1.40645
C	-0.67395	-6.85968	-0.44427
C	-2.0516	-5.30389	-1.91028
C	0.341115	-5.88883	-2.56599
N	-0.15304	-4.488	-0.64313
H	-1.25764	-6.58247	0.444542
H	0.35655	-7.0839	-0.11974
O	-1.28789	-8.02636	-0.98471
H	-2.00388	-4.37767	-2.49211
H	-2.71235	-5.13773	-1.04296
O	-2.60496	-6.29324	-2.77268
H	0.30913	-5.03268	-3.25761
H	1.358451	-5.96568	-2.16061
O	0.097558	-7.10255	-3.27218
H	0.723459	-4.74123	-0.18639

H	-0.82047	-4.29838	0.110128
H	-2.62156	-7.12705	-2.27973
H	-0.77575	-8.28167	-1.76618
H	-0.79071	-7.04344	-3.65177

Ce_6(Tris-depr)(H_2O)$_6$(OH)$_5$(μ_3–OH)$_4$(μ_3–O)$_4$(HCO_2)$_6$ Terminal –OH-1

C	1.704349	-2.3606	4.024418
O	-2.11501	-0.95804	3.788673
O	0.527395	-2.60448	3.663143
Ce	-0.94741	-1.80117	1.850526
O	3.905087	-2.15267	0.864656
O	2.773849	-3.21861	-1.36388
O	0.967385	-1.70901	0.772256
O	2.534414	-1.62244	3.424929
H	3.57319	-2.60087	0.045283
H	4.793348	-1.84073	0.651001
H	-0.18982	-4.37182	0.281124
H	3.346268	-0.40423	-1.29708
C	-2.31644	0.205304	4.236263
C	1.950126	2.374074	3.80041
O	-1.97313	1.283162	3.693775
O	0.339844	0.02263	2.713901
O	0.166661	4.238642	0.970978
O	0.799469	2.722127	3.417683
O	-1.62518	0.103892	0.925113
Ce	-0.68012	1.920905	1.67809
H	-3.11006	3.110658	2.356783
O	3.599322	2.257043	-1.12146
O	4.28675	0.813484	1.04146
O	1.194575	1.459617	0.630271
O	2.67133	1.485506	3.283193
O	2.47011	-0.30033	-0.90742
Ce	2.37874	-0.18794	1.485257
H	4.663216	1.387866	1.719219
H	3.94514	1.782279	-0.31981
C	-1.66623	-2.43086	-3.58373
O	-3.49922	-2.08392	1.575037
O	-4.07522	-1.00675	-0.79102
O	-1.35791	-2.11617	-0.49117
O	-2.34702	-1.48251	-3.12322
H	-3.79302	-1.69861	0.701971
H	-3.94947	-1.57267	2.259295
H	-4.30308	-1.75861	-1.35239
O	1.965859	-1.40819	-3.52478
O	-0.53971	-2.81531	-3.16624
O	0.301381	-4.38679	-0.57778
Ce	0.910815	-2.05651	-1.40238
H	3.425971	-3.03918	-2.05319
H	1.07084	-4.95295	-0.44416
C	-1.45432	2.11774	-3.77733
O	-3.79833	1.820605	-0.43523
O	-2.549	3.088635	1.572221
O	-1.02052	2.123905	-0.6855
O	-2.29468	1.404966	-3.16406
Ce	-2.16827	0.027416	-1.19065
H	0.576793	4.189555	0.069573
H	-0.5657	4.862664	0.892456
H	-3.43107	2.342803	0.327741
H	-4.64779	1.467165	-0.14569
H	-1.42498	2.942139	-0.9967
C	2.37197	-0.33541	-4.03388
O	2.23168	0.821302	-3.55104
O	-0.06385	-0.14095	-1.76702
O	1.255313	3.849351	-1.48299
O	-0.25252	2.292031	-3.45953
Ce	1.16693	1.656209	-1.55872
H	0.666378	4.340603	-2.06985
H	3.733917	3.199151	-0.96337
H	0.42231	0.059557	3.673389
H	-1.89874	-2.88366	-0.71211
H	-2.09185	-2.98632	-4.4424
H	-1.81532	2.63899	-4.68578
H	2.905891	-0.40694	-5.00179
H	-2.85052	0.275762	5.204339
H	2.362099	2.908439	4.679264
H	2.054327	-2.83969	4.960128
C	-2.43224	-5.95244	1.93891
C	-1.70566	-4.77963	2.621856
C	-3.44255	-5.44991	0.895536
C	-3.1426	-6.80522	3.003043
N	-1.50435	-6.84395	1.237153
H	-0.96103	-5.19871	3.321018
H	-2.42203	-4.19435	3.224617
O	-1.07119	-3.94277	1.684608
H	-3.91622	-6.31861	0.418683
H	-2.91861	-4.87716	0.11337
O	-4.48116	-4.66129	1.467888
H	-3.6596	-7.63492	2.491916
H	-2.39296	-7.24277	3.67995
O	-4.05677	-6.06939	3.808975

H	-0.75401	-7.11616	1.869029
H	-1.06467	-6.34806	0.465949
H	-4.1575	-3.74427	1.568142
H	-4.53087	-5.46945	3.20828

Ce_6(Tris-depr)$(H_2O)_6(OH)_5(\mu_3\text{–}OH)_4(\mu_3\text{–}O)_4(HCO_2)_6$ Terminal –OH-2

C	1.471019	-2.10513	4.280142
O	-2.35976	-0.7359	3.716948
O	0.33522	-2.38554	3.827457
O	-1.23567	-3.83502	1.935994
Ce	-1.04628	-1.65364	1.905316
H	-0.90734	-4.30324	2.713221
O	3.888516	-1.94929	1.415441
O	0.962421	-1.54107	1.025823
O	2.317503	-1.32662	3.757626
H	3.745945	-2.63181	2.084006
H	3.691459	-2.37915	0.560904
H	-0.11402	-4.35977	0.577261
H	3.538686	-0.29625	-0.88956
C	-2.63543	0.443931	4.068568
C	1.542825	2.708568	3.882107
O	-2.29947	1.497333	3.472785
O	0.122421	0.245142	2.798447
O	-0.03032	4.344077	0.770661
O	0.434568	3.002591	3.355296
O	-1.68683	0.14572	0.82428
Ce	-0.85166	2.038473	1.546268
H	-3.37179	3.178226	1.905085
O	3.669496	2.3667	-0.84415
O	4.17477	1.098931	1.457016
O	1.129548	1.588144	0.711185
O	2.335227	1.812126	3.50088
O	2.626692	-0.23581	-0.58199
Ce	2.267598	0.041249	1.773684
H	4.457894	1.722578	2.13651
H	3.943779	1.960689	0.022123
C	-1.14316	-2.67886	-3.50224
O	-3.53508	-2.08194	1.376209
O	-3.88914	-1.13266	-1.13766
O	-1.1887	-2.1444	-0.42864
O	-1.87374	-1.70498	-3.19943
H	-3.75439	-1.77022	0.461315
H	-3.70591	-3.03185	1.377254
H	-4.01669	-1.93578	-1.65854
O	2.299496	-1.42627	-3.24276
O	-0.08322	-3.03045	-2.91607
O	0.68313	-4.27392	0.016196
Ce	1.177782	-2.08168	-1.10007
H	0.741934	-4.97744	-0.65901
C	-1.07795	1.881796	-3.97491
O	-3.73748	1.686935	-0.86227
O	-2.73644	3.11565	1.181751
O	-0.9622	2.088091	-0.85242
O	-1.95792	1.178803	-3.40742
Ce	-1.973	-0.078	-1.35135
H	0.474284	4.251144	-0.07818
H	-0.78114	4.915674	0.566474
H	-3.46823	2.261968	-0.09743
H	-4.58597	1.292352	-0.62744
H	-1.36277	2.872597	-1.24451
C	2.786511	-0.385	-3.7467
O	2.633982	0.786856	-3.31058
O	0.171517	-0.19497	-1.67696
O	1.325687	3.838963	-1.52311
O	0.080056	2.11089	-3.54843
Ce	1.314361	1.6431	-1.4794
H	0.79068	4.276946	-2.19717
H	3.731664	3.322837	-0.73192
H	0.11415	0.336089	3.757668
H	-1.6837	-2.93676	-0.66609
H	-1.45628	-3.29334	-4.36909
H	-1.35734	2.339187	-4.94436
H	3.404855	-0.50404	-4.65734
H	-3.23445	0.557845	4.993543
H	1.843712	3.303528	4.767046
H	1.766676	-2.58558	5.233723
C	3.838784	-5.28218	-2.10785
C	3.736204	-4.35576	-0.87453
C	4.044839	-4.48889	-3.41058
C	2.642706	-6.23778	-2.23638
N	5.010408	-6.15639	-1.94716
H	4.769344	-4.06468	-0.617
H	3.339732	-4.93785	-0.02355
O	2.984363	-3.17978	-1.04036
H	4.505621	-5.16768	-4.14236
H	4.744174	-3.65526	-3.23716
O	2.833995	-4.00699	-3.98246
H	2.7667	-6.8407	-3.15066
H	2.645871	-6.9242	-1.37758
O	1.371613	-5.59912	-2.2426

H	4.985632	-6.58875	-1.02498
H	5.858573	-5.593	-1.97073
H	2.709605	-3.06606	-3.75282
H	1.400125	-4.91629	-2.93254

$Ce_6(MOPS)(H_2O)_5(OH)_6(\mu_3–OH)_4(\mu_3–O)_4(HCO_2)_6$ Terminal $–OH_2$-1

C	1.7229	-2.87349	3.63083
O	-2.26533	-1.91786	3.209078
O	0.643959	-3.22536	3.099456
O	-1.02831	-4.48935	1.27848
Ce	-0.85948	-2.34893	1.279744
H	-0.3567	-4.99831	1.74802
O	4.132391	-1.88495	0.832662
O	3.351133	-2.79684	-1.52204
O	1.15909	-1.89488	0.452033
O	2.467659	-1.92242	3.260214
H	3.919594	-2.28529	-0.05697
H	4.960371	-1.40346	0.715011
H	3.491558	0.016283	-1.15703
C	-2.64622	-0.8657	3.789963
C	1.242341	1.850292	4.054377
O	-2.42754	0.313804	3.417147
O	0.096148	-0.52741	2.507224
O	-0.51966	3.838154	1.29847
O	0.095326	2.101123	3.591041
O	-1.73227	-0.45465	0.557612
Ce	-1.08813	1.352863	1.619645
H	-3.71483	2.093409	2.170575
O	3.329983	2.633553	-0.61521
O	4.00903	1.024637	1.416089
O	0.914585	1.291829	0.732858
O	2.123451	1.135769	3.517932
O	2.576675	-0.05965	-0.86122
Ce	2.242882	-0.30155	1.49061
H	4.181327	1.557639	2.201694
H	3.656873	2.105766	0.162762
C	-0.943	-2.24073	-4.26619
O	-3.27769	-2.97816	0.625379
O	-3.7961	-1.59384	-1.62331
O	-0.9486	-2.38013	-1.09266
O	-1.8348	-1.5391	-3.72617
H	-3.55026	-2.52344	-0.21287
H	-3.28607	-3.92284	0.427327
H	-3.83284	-2.27023	-2.31171
O	2.524114	-0.81733	-3.61555
O	0.215792	-2.44124	-3.8225
Ce	1.339556	-1.9243	-1.74755
H	3.444511	-3.75211	-1.61527
C	-1.38411	2.207943	-3.82459
O	-3.94877	1.153147	-0.83429
O	-3.08976	2.267891	1.456496
O	-1.23834	1.832511	-0.72376
O	-2.16501	1.312533	-3.4027
Ce	-2.01148	-0.3076	-1.61716
H	-0.02684	3.971956	0.447665
H	-1.3154	4.379837	1.228674
H	-3.73574	1.593891	0.031111
H	-4.76564	0.658816	-0.69785
H	-1.73672	2.619804	-0.97169
C	2.805516	0.359834	-3.94011
O	2.435135	1.410453	-3.34489
O	0.137685	-0.10417	-1.96769
O	0.826688	3.94103	-1.04767
O	-0.25267	2.489499	-3.35945
Ce	1.063992	1.783802	-1.40461
H	0.229792	4.427792	-1.6299
H	3.303217	3.555383	-0.33268
H	0.091725	-0.61706	3.466569
H	-1.28283	-3.20084	-1.51185
H	-1.199	-2.72266	-5.23017
H	-1.7337	2.802213	-4.69188
H	3.442443	0.498832	-4.83597
H	-3.23359	-0.99718	4.720346
H	1.491738	2.310187	5.031355
H	2.065906	-3.45042	4.513026
C	-3.75545	-8.9299	0.047756
N	-2.43956	-9.56177	-0.32863
C	-2.57602	-10.4548	-1.53377
C	-1.24793	-8.64543	-0.33093
H	-2.23342	-10.2018	0.443632
O	-4.69818	-9.44602	-2.12445
C	-4.54594	-8.42605	-1.14903
C	-3.43061	-9.83932	-2.62974
H	-4.08499	-7.53817	-1.60696
H	-5.54726	-8.14929	-0.798
H	-3.60113	-10.6052	-3.39576
H	-2.93657	-8.97856	-3.10519
H	-4.32737	-9.71564	0.555327
H	-3.55009	-8.12032	0.757118
H	-3.0501	-11.376	-1.17527

H	-1.56803	-10.6875	-1.89556
H	-1.25975	-8.14887	0.647076
H	-0.37228	-9.30517	-0.37408
C	-1.19979	-7.61541	-1.44929
H	-2.11877	-7.02036	-1.46439
H	-1.0834	-8.10215	-2.42556
C	-0.02188	-6.68189	-1.19128
H	-0.09078	-6.21073	-0.20296
H	0.948785	-7.18515	-1.28786
S	-0.00233	-5.32574	-2.35117
O	1.039735	-4.37753	-1.7953
O	0.377363	-5.85465	-3.68393
O	-1.36898	-4.7153	-2.3308

$Ce_6(MOPS)(H_2O)_5(OH)_6(\mu_3–OH)_4(\mu_3–O)_4(HCO_2)_6$ Terminal $–OH_2$-2

C	-0.21623	-2.59586	4.40156
O	-3.3062	-0.30935	3.169716
O	-1.27047	-2.57417	3.717429
O	-2.55882	-3.6404	1.414466
Ce	-1.93275	-1.52749	1.584571
H	-2.48655	-4.18123	2.210299
O	1.933238	-4.11533	-0.75686
O	0.147865	-1.99164	1.099574
O	0.897225	-2.10294	4.07818
H	-1.46472	-4.28873	0.286533
H	3.225575	-1.54389	-0.1791
C	-3.32019	0.893807	3.549005
C	1.233874	1.921243	4.309634
O	-2.59741	1.822767	3.111486
O	-0.51217	-0.02972	2.775877
O	0.832972	3.977582	1.053283
O	0.395138	2.530923	3.588356
O	-1.87862	0.400485	0.527147
Ce	-0.71034	1.982612	1.510597
H	-2.86715	3.744425	1.51238
O	4.103542	1.109062	-0.05538
O	3.750079	-0.07748	2.271188
O	1.189508	1.016764	1.010288
O	1.805578	0.839377	4.035437
O	2.350088	-1.12477	-0.10553
Ce	1.671012	-0.8113	2.15918
H	3.915791	0.571161	2.965819
H	4.061359	0.684707	0.849359
C	-1.31167	-2.33829	-3.84621
O	-4.29765	-1.32918	0.595977
O	-3.93849	-0.1855	-1.83881
O	-1.76588	-1.90928	-0.77448
O	-1.82833	-1.22413	-3.58432
H	-4.25888	-0.92355	-0.30789
H	-4.70863	-2.19438	0.477256
H	-4.17503	-0.89309	-2.45193
O	2.296849	-2.16633	-2.82587
O	-0.48067	-2.96414	-3.13497
O	-0.81007	-4.51433	-0.42727
Ce	0.600588	-2.46904	-1.02813
H	2.876026	-3.89283	-0.73551
H	-0.25916	-5.22687	-0.08184
C	0.056938	2.014201	-3.94851
O	-3.08998	2.495228	-1.37095
O	-2.13772	3.554841	0.909972
O	-0.36032	2.100645	-0.85346
O	-1.06805	1.56648	-3.59381
Ce	-1.80225	0.316769	-1.66845
H	1.438529	3.769171	0.294471
H	0.324723	4.750765	0.777932
H	-2.81762	2.960762	-0.53582
H	-4.04268	2.355966	-1.30994
H	-0.45077	2.971237	-1.25804
C	3.090166	-1.26505	-3.17984
O	3.141361	-0.08885	-2.72114
O	0.26288	-0.38323	-1.64796
O	2.385754	3.176877	-1.00847
O	1.134673	1.906922	-3.31538
Ce	1.800172	1.05439	-1.0985
H	2.097985	3.739758	-1.73824
H	4.41382	2.012406	0.079634
H	-0.66957	0.045798	3.723269
H	-2.40733	-2.52043	-1.15501
H	-1.61407	-2.82084	-4.79676
H	0.091835	2.55381	-4.91551
H	3.821229	-1.51531	-3.97417
H	-4.04436	1.152767	4.346812
H	1.486277	2.39484	5.279286
H	-0.26893	-3.09967	5.387209
C	5.990252	-8.11755	-1.32731
N	6.158046	-8.08727	0.170029
C	7.591423	-7.83953	0.559629
C	5.133898	-7.29392	0.933097
H	5.970754	-9.04899	0.467158
O	8.121325	-7.0181	-1.65919
C	6.752812	-7.01194	-2.03707
C	8.250524	-6.74948	-0.27075
H	6.314978	-6.0202	-1.84549
H	6.704234	-7.20594	-3.11519
H	9.320528	-6.74492	-0.03125
H	7.842899	-5.75236	-0.04698
H	6.376984	-9.09153	-1.64992
H	4.918057	-8.06676	-1.54596
H	8.115207	-8.7891	0.397765

H	7.613043	-7.59702	1.628171
H	4.160549	-7.61269	0.540235
H	5.216133	-7.63828	1.971781
C	5.27509	-5.78106	0.870485
H	5.261703	-5.42618	-0.16575
H	6.21679	-5.46439	1.336079
C	4.100973	-5.16204	1.620792
H	3.143309	-5.37393	1.129308
H	4.050999	-5.49479	2.666054
S	4.242458	-3.38308	1.689985
O	2.905212	-2.92472	2.231615
O	5.36255	-3.05525	2.605316
O	4.460644	-2.89458	0.291748

$Ce_6(MOPS\text{-}depr)(H_2O)_6(OH)_5(\mu_3\text{–}OH)_4(\mu_3\text{–}O)_4(HCO_2)_6$ Terminal –OH-1

C	1.012866	-2.53307	3.82728
O	-2.50327	-0.48884	3.708979
O	-0.19888	-2.55441	3.497965
O	-1.94994	-3.62389	1.562909
Ce	-1.53582	-1.47888	1.736286
H	-1.6238	-4.20704	2.259115
O	3.61773	-2.26892	0.986592
O	0.389272	-1.70121	0.631255
O	1.940873	-1.92573	3.227379
H	3.270224	-3.18043	0.869898
H	4.235296	-2.1185	0.26033
H	-1.23617	-4.09993	0.070676
H	2.947741	-0.7496	-1.41185
C	-2.51343	0.685127	4.171859
C	1.960452	2.159483	3.745111
O	-2.0241	1.704304	3.626113
O	0.041757	0.094167	2.600539
O	0.494003	4.303417	0.89571
O	0.900797	2.702778	3.329207
O	-1.93085	0.488726	0.848933
Ce	-0.69077	2.146325	1.60148
H	-2.90762	3.677928	2.301839
O	3.560429	1.872473	-1.29204
O	3.994848	0.537774	0.932022
O	1.085904	1.409517	0.528623
O	2.529583	1.158727	3.243099
O	2.098867	-0.54881	-0.99916
Ce	2.039036	-0.36806	1.381433
H	4.397554	1.091244	1.611618
H	3.824637	1.408028	-0.4463
C	-2.31258	-1.91477	-3.70854
O	-4.07037	-1.51857	1.43467
O	-4.49591	-0.20235	-0.89993
O	-1.9739	-1.71692	-0.60818
O	-2.8649	-0.89818	-3.22342
H	-4.32705	-1.05405	0.597703
H	-4.39909	-2.42185	1.344434
H	-4.85435	-0.88179	-1.48529
O	1.437582	-1.58222	-3.56672
O	-1.25846	-2.46445	-3.28681
O	-0.81252	-4.16428	-0.82318
Ce	0.241407	-1.98837	-1.48386
H	-0.26113	-4.96731	-0.8416
C	-1.4427	2.488667	-3.85708
O	-3.79589	2.543746	-0.50159
O	-2.35547	3.574455	1.517213
O	-1.00879	2.398685	-0.76214
O	-2.38692	1.93143	-3.23425
Ce	-2.47618	0.535576	-1.2805
H	0.876195	4.209113	-0.01339
H	-0.11163	5.053624	0.851052
H	-3.35227	2.995872	0.264468
H	-4.69169	2.327999	-0.21595
H	-1.27724	3.275884	-1.0604
C	1.984587	-0.58276	-4.09678
O	1.982336	0.594	-3.64691
O	-0.39894	0.018699	-1.85096
O	1.464022	3.771127	-1.58489
O	-0.22638	2.452102	-3.54929
Ce	1.085835	1.619988	-1.65993
H	0.942464	4.344286	-2.16115
H	3.81627	2.797211	-1.19023
H	0.127921	0.099704	3.560807
H	-2.63084	-2.37998	-0.85219
H	-2.79274	-2.37802	-4.59218
H	-1.71652	3.064182	-4.76284
H	2.520195	-0.7507	-5.05125
H	-3.00423	0.82572	5.154774
H	2.43271	2.611328	4.639238
H	1.298254	-3.10512	4.731975
C	7.198292	-8.97998	-2.28866
N	5.936913	-8.69761	-1.60124
C	4.98868	-9.77115	-1.90822
C	5.428658	-7.39686	-2.03163
O	6.796523	-11.3729	-2.20386
C	7.738113	-10.3463	-1.90574
C	5.561556	-11.1284	-1.53823
H	7.984062	-10.3746	-0.82927
H	8.648295	-10.5674	-2.47784
H	4.869054	-11.9238	-1.8417
H	5.713869	-11.194	-0.44618
H	7.061824	-8.94283	-3.38928
H	7.939954	-8.21478	-2.01973
H	4.731247	-9.76536	-2.98762

H	4.059278	-9.62782	-1.34307
H	6.242507	-6.66551	-1.91922
H	5.16715	-7.42127	-3.11022
C	4.219422	-6.91593	-1.23393
H	4.477208	-6.8547	-0.16726
H	3.383801	-7.62001	-1.34016
C	3.781618	-5.5471	-1.7434
H	4.546405	-4.77357	-1.58726
H	3.508926	-5.56942	-2.80762
S	2.330482	-4.96493	-0.89551
O	1.997119	-3.63641	-1.5507
O	1.242165	-5.96075	-1.09972
O	2.670129	-4.76819	0.544644

Ce_6(MOPS-depr)$(H_2O)_6(OH)_5(\mu_3\text{–}OH)_4(\mu_3\text{–}O)_4(HCO_2)_6$
Terminal –OH-2

C	1.333782	-1.88528	4.295328
O	-2.25372	-0.25229	3.800386
O	0.187483	-2.13491	3.831517
Ce	-1.10041	-1.30227	1.973919
O	3.822921	-1.89685	1.454592
O	2.881659	-3.1878	-0.72286
O	0.877664	-1.43238	1.084081
O	2.200765	-1.13549	3.7793
H	3.563597	-2.42752	0.656927
H	4.742914	-1.6405	1.313091
H	-0.0939	-4.25939	0.852944
H	3.458733	-0.44065	-0.93597
C	-2.48798	0.960252	4.064452
C	1.759478	2.950419	3.658697
O	-2.13586	1.950992	3.379374
O	0.179522	0.518414	2.737655
O	0.205168	4.495078	0.445487
O	0.670476	3.287607	3.117738
O	-1.65197	0.389773	0.826467
Ce	-0.70546	2.317077	1.395542
H	-3.16314	3.573448	1.73636
O	3.746395	2.21747	-1.09082
O	4.236792	1.073754	1.293974
O	1.210166	1.677861	0.567021
O	2.482595	1.977322	3.330602
O	2.557144	-0.2768	-0.63393
Ce	2.301137	0.137808	1.707383
H	4.582751	1.723709	1.917737
H	4.020759	1.859767	-0.20587
C	-1.40376	-2.76291	-3.29056
O	-3.60523	-1.51193	1.625689
O	-3.96859	-0.90443	-0.99184
O	-1.30874	-1.98946	-0.284
O	-2.07668	-1.73217	-3.0477
H	-3.86144	-1.34072	0.685779
H	-4.04291	-2.33552	1.897912
H	-4.12319	-1.76809	-1.39624
O	2.213191	-1.70958	-3.1376
O	-0.31595	-3.09078	-2.74091
O	0.472249	-4.31014	0.063812
Ce	1.018368	-2.07318	-1.03158
H	3.578936	-3.07082	-1.38057
H	1.28534	-4.7525	0.338217
C	-1.08428	1.762319	-4.09979
O	-3.67411	1.909679	-0.91456
O	-2.54785	3.432062	1.006391
O	-0.88515	2.167428	-0.99213
O	-1.98429	1.140649	-3.47026
Ce	-2.02651	0.030991	-1.3461
H	0.680757	4.325351	-0.4088
H	-0.50213	5.116947	0.232792
H	-3.36181	2.517729	-0.19474
H	-4.54469	1.589412	-0.64936
H	-1.25361	2.941045	-1.43525
C	2.684824	-0.7148	-3.74052
O	2.547651	0.494108	-3.40837
O	0.106252	-0.21648	-1.71483
O	1.461905	3.765001	-1.83592
O	0.087281	1.974706	-3.70477
Ce	1.350441	1.589869	-1.63745
H	0.955087	4.193557	-2.53737
H	3.894829	3.170263	-1.06228
H	0.198903	0.661394	3.690849
H	-1.86775	-2.77768	-0.41538
H	-1.79598	-3.45209	-4.06336
H	-1.3601	2.156535	-5.09735
H	3.279451	-0.91388	-4.65336
H	-3.05948	1.162164	4.991167
H	2.10898	3.576433	4.502849
H	1.599554	-2.37704	5.251058
C	-3.59584	-10.9284	1.392507
N	-4.05008	-9.57762	1.058903
C	-4.40859	-9.54114	-0.36056
C	-2.99356	-8.61767	1.370303
O	-4.98855	-11.9046	-0.3401
C	-4.64932	-11.9639	1.042074
C	-5.44583	-10.6013	-0.688
H	-5.55822	-11.8091	1.650473
H	-4.26644	-12.9738	1.237442
H	-5.64834	-10.6083	-1.76661
H	-6.39032	-10.3898	-0.15575
H	-2.65796	-11.1719	0.851633
H	-3.38274	-10.9859	2.469228
H	-3.51088	-9.69957	-0.99339

H	-4.8301	-8.56151	-0.61873
H	-2.68164	-8.79169	2.410689
H	-2.10249	-8.79963	0.733925
C	-3.42574	-7.16113	1.222713
H	-4.32878	-6.97909	1.821505
H	-3.66235	-6.93223	0.174913
C	-2.30399	-6.24092	1.694458
H	-2.1003	-6.34361	2.769004
H	-1.37112	-6.40093	1.135385
S	-2.72543	-4.53122	1.442475
O	-1.54718	-3.73926	2.013316
O	-2.84296	-4.2892	-0.02435
O	-3.9731	-4.2366	2.195652

$Ce_6(H_3BO_3)(H_2O)_6(OH)_5(\mu_3\text{-}OH)_4(\mu_3\text{-}O)_4(HCO_2)_6$ Terminal –OH_2-2

C	1.201774	-2.40391	3.973845
O	-2.54795	-0.93037	3.460736
O	0.070385	-2.63089	3.476625
O	-1.34407	-3.88922	1.346383
Ce	-1.22774	-1.69151	1.57395
H	-1.05762	-4.43419	2.088915
O	3.706372	-2.06904	1.089383
O	2.813984	-3.04384	-1.25717
O	0.784263	-1.57868	0.707993
O	2.08563	-1.63436	3.508896
H	3.440775	-2.48926	0.228533
H	4.607313	-1.74871	0.957035
H	-0.32406	-4.19981	0.01426
H	3.397776	-0.27523	-1.0305
C	-2.76005	0.212991	3.954184
C	1.498229	2.354332	4.032453
O	-2.3697	1.310385	3.489561
O	-0.00935	0.053366	2.663801
O	-0.11637	4.446761	1.190597
O	0.396233	2.731774	3.547448
O	-1.79165	0.216482	0.68456
Ce	-0.86762	2.010253	1.594522
H	-3.38211	3.074546	2.128079
O	4.172137	0.772698	1.557267
O	1.094566	1.623294	0.810016
O	2.260494	1.474097	3.562149
O	2.488721	-0.12508	-0.74466
Ce	2.128713	-0.08905	1.617012
H	4.358416	1.525977	2.12964
C	-1.37815	-2.14234	-3.93263
O	-3.71498	-2.03481	0.98655
O	-4.03462	-0.77084	-1.37713
O	-1.39065	-1.95262	-0.78886
O	-2.09772	-1.20136	-3.51805
H	-3.91768	-1.59359	0.119228
H	-3.91833	-2.96945	0.857599
H	-4.18197	-1.49796	-1.99492
O	2.2711	-1.2227	-3.45692
O	-0.31971	-2.5716	-3.39738
O	0.268313	-4.21818	-0.78332
Ce	0.958633	-1.843	-1.47142
H	3.525797	-2.83552	-1.87515
H	1.034777	-4.75232	-0.54331
C	-1.20305	2.524088	-3.90749
O	-3.80847	1.998003	-0.78507
O	-2.76339	3.15861	1.392703
O	-0.99831	2.334473	-0.75654
O	-2.05828	1.738169	-3.41022
Ce	-2.06999	0.25059	-1.5044
H	0.362129	4.573309	0.348315
H	-0.89408	5.016677	1.135576
H	-3.50803	2.470557	0.038154
H	-4.65417	1.585501	-0.57258
H	-1.42017	3.141967	-1.0714
C	2.755754	-0.15871	-3.92311
O	2.567752	1.002846	-3.4824
O	0.012396	0.030965	-1.90241
O	1.227799	4.528952	-1.28914
O	-0.02813	2.69643	-3.50647
Ce	1.315457	1.903003	-1.53858
H	0.632555	4.95077	-1.9245
H	-0.01571	0.045882	3.62676
H	-1.92472	-2.68366	-1.11921
H	-1.7015	-2.65054	-4.86286
H	-1.53274	3.114089	-4.78625
H	3.409634	-0.25985	-4.81286
H	-3.354	0.242279	4.889849
H	1.823213	2.853618	4.967408
H	1.451794	-2.93418	4.914879
H	2.015882	5.08704	-1.25908
H	3.807263	3.65427	-1.80365
B	4.943762	2.068903	-1.57448
O	5.662918	2.412179	-2.69273
H	6.449723	1.866922	-2.8144
O	5.296997	1.027702	-0.78298
H	4.831103	0.972194	0.106289
O	3.813841	2.83894	-1.28275

$Ce_6(Tris)(H_2O)_6(OH)_5(\mu_3\text{-}OH)_4(\mu_3\text{-}O)_4(HCO_2)_6$ Terminal –OH_2-1

C	1.257668	-2.33482	4.14091
O	-2.67901	-0.4977	3.665307
O	0.10251	-2.56463	3.714719
O	-1.89894	-4.15111	2.120591
Ce	-1.36409	-1.60618	1.8523
H	-1.15096	-4.75193	2.235006
O	3.598437	-2.26973	1.068486
O	2.521879	-3.25887	-1.19592
O	0.742332	-1.65243	0.798796
O	2.138234	-1.61867	3.582958
H	3.270449	-2.69217	0.23043
H	4.504379	-1.99186	0.88537
H	-0.8857	-4.44722	-0.20539
H	3.276139	-0.48783	-1.12679
C	-2.71351	0.675203	4.116679
C	1.788892	2.425042	3.876147
O	-2.18556	1.69538	3.599801
O	0.061519	0.172251	2.741046
O	0.304039	4.386333	0.93713
O	0.698941	2.85999	3.413728
O	-1.85227	0.448639	0.813996
Ce	-0.77448	2.110918	1.6141
H	-3.11103	3.569036	2.123373
O	3.688329	2.164292	-0.88082
O	4.147987	0.657368	1.282117
O	1.130311	1.516418	0.673651
O	2.467674	1.471048	3.422865
O	2.391904	-0.31652	-0.78412
Ce	2.123934	-0.2208	1.602862
H	4.487429	1.20615	1.999177
H	3.94456	1.65597	-0.06267
C	-1.74733	-2.18919	-3.67487
O	-4.21657	-0.45619	-1.05444
O	-1.5893	-1.88508	-0.58198
O	-2.3804	-1.18484	-3.26928
H	-4.551	-1.31071	-1.38394
O	1.949271	-1.41273	-3.42026
O	-0.67104	-2.64242	-3.1974
O	-0.05512	-4.32944	-0.68213
Ce	0.725691	-1.96373	-1.33083
H	3.19924	-3.14831	-1.87454
H	0.592614	-4.89418	-0.24234
C	-1.18849	2.327921	-3.89846
O	-3.73683	2.323131	-0.70911
O	-2.50916	3.492291	1.373665
O	-0.9312	2.357718	-0.77426
O	-2.11832	1.704673	-3.31997
Ce	-2.21006	0.33712	-1.3054
H	0.755699	4.295771	0.05974
H	-0.40549	5.025799	0.798338
H	-3.364	2.806027	0.077602
H	-4.60548	1.998578	-0.44356
H	-1.25398	3.197236	-1.11954
C	2.469652	-0.37826	-3.90263
O	2.39252	0.790296	-3.43314
O	-0.0786	0.012207	-1.77644
O	1.494356	3.904039	-1.46062
O	0.002828	2.422264	-3.51194
Ce	1.253619	1.707071	-1.50279
H	0.955147	4.413628	-2.07846
H	3.869578	3.094127	-0.69994
H	0.10773	0.232675	3.701214
H	-2.20314	-2.56179	-0.89016
H	-2.17138	-2.73606	-4.54091
H	-1.4504	2.843214	-4.84423
H	3.055754	-0.49736	-4.8357
H	-3.26783	0.83255	5.06467
H	2.185547	2.942819	4.773023
H	1.549909	-2.80522	5.102161
C	-5.71729	-3.64312	1.111143
C	-4.91299	-2.5651	1.868513
C	-4.94371	-4.96446	0.958193
C	-6.22807	-3.16145	-0.25909
N	-6.90904	-3.96596	1.906037
H	-4.52686	-3.00285	2.800514
H	-5.60131	-1.74863	2.138356
O	-3.79625	-2.0302	1.172547
H	-5.60746	-5.70283	0.488193
H	-4.6673	-5.34054	1.957149
O	-3.78179	-4.86296	0.136252
H	-6.99341	-3.87772	-0.59908
H	-6.71134	-2.17783	-0.14915
O	-5.21612	-3.03493	-1.25772

H	-7.48962	-3.13485	2.002746
H	-6.62913	-4.22882	2.84965
H	-3.05386	-4.52061	0.681947
H	-4.07532	-1.42984	0.447325
H	-4.49992	-3.65774	-1.03625
H	-2.51791	-4.36758	2.831259

H_3BO_3

B	-0.09058	0.243138	-0.8068
O	-0.20912	1.089655	0.26818
H	-1.10631	1.127209	0.62195
O	-1.18801	-0.44603	-1.26232
H	-0.9915	-1.00831	-2.02183
O	1.125167	0.084763	-1.42595
H	1.824381	0.617033	-1.02678

$H_2BO_3^-$

B	-0.00988	0.240918	-0.83924
O	-0.21945	1.094518	0.275723
H	-1.13652	1.076052	0.571833
O	-1.20628	-0.42414	-1.22881
H	-1.03717	-0.99372	-1.98709
O	1.159791	0.101818	-1.42013

HBO_3^{2-}

B	-0.56784	-0.16201	0.452282
O	0.129272	0.809205	1.081746
O	-1.85402	-0.51043	1.089628
O	-0.24893	-0.84842	-0.67213
H	-2.27632	-1.20158	0.568745

Tris

C	-0.83474	-0.03155	0.345514
C	-1.45517	0.088464	-1.06035
H	-0.64172	0.179138	-1.79427
H	-2.06028	1.010647	-1.1052
C	0.015483	-1.30513	0.473783
H	0.532069	-1.29035	1.442824
H	0.780644	-1.31087	-0.3223
C	-1.90984	0.031681	1.442172
H	-1.43171	-0.10957	2.425279
H	-2.37295	1.029026	1.428073
N	0.065565	1.100365	0.58347
H	-0.43389	1.97436	0.429747
H	0.828134	1.081329	-0.09108
O	-0.74561	-2.50992	0.434882
H	-1.23199	-2.51537	-0.40288
O	-2.23946	-1.03473	-1.46311
H	-2.95047	-1.13018	-0.81216
O	-2.96995	-0.90615	1.266536
H	-2.57776	-1.79087	1.290582

Tris-depr

C	-0.8325	-0.04504	0.356755
C	-1.45619	0.000019	-1.06817
H	-0.62602	0.300326	-1.74687
H	-2.17826	0.847521	-1.0824
C	-0.02441	-1.34346	0.508567
H	0.388907	-1.41771	1.527509
H	0.829169	-1.31743	-0.19613
C	-1.88489	0.059506	1.471248
H	-1.36065	0.068635	2.443235
H	-2.42333	1.015225	1.371257
N	0.081222	1.083948	0.567997
H	-0.42654	1.95659	0.431205
H	0.803236	1.065862	-0.14976
O	-0.84887	-2.47525	0.253592
H	-1.4129	-2.13816	-0.55664
O	-2.04774	-1.19987	-1.49316
O	-2.86419	-0.97436	1.458535
O	-2.39921	-1.79588	1.22689

TrisH$^+$

C	-0.85237	-0.0613	0.349306
C	-1.45964	0.11787	-1.0545
H	-0.64802	0.305891	-1.77171
H	-2.13284	0.991329	-1.05616
C	0.016299	-1.32944	0.450864
H	0.626141	-1.27172	1.363675
H	0.69641	-1.37775	-0.41591
C	-1.91698	0.01256	1.460265
H	-1.44877	-0.20888	2.433866
H	-2.32127	1.034394	1.496766
N	0.069874	1.102415	0.567481
H	-0.41368	1.996221	0.418082
H	0.871083	1.072793	-0.07487
O	-0.76088	-2.50875	0.55282
H	-1.22813	-2.62484	-0.28678
O	-2.13598	-1.04513	-1.49702
H	-2.90266	-1.17368	-0.91999
O	-3.0115	-0.85181	1.214393
H	-2.68958	-1.76172	1.28538
H	0.443069	1.109547	1.524607

MOPS

N	-0.20701	0.418332	-2.15219
O	-0.34778	-0.37677	0.621195
C	-1.50595	-0.50412	-0.19015
H	-1.60358	-1.54279	-0.54052
H	-2.37688	-0.26888	0.433476
C	0.816077	-0.68926	-0.12993
H	1.678785	-0.60196	0.541281
H	0.760142	-1.72886	-0.48679
C	-1.47567	0.486039	-1.34229
H	-1.52106	1.507239	-0.94541
H	-2.31877	0.329845	-2.02447
C	1.007431	0.297283	-1.27058
H	1.182297	1.299169	-0.86058
H	1.856423	0.016373	-1.90398
C	-0.23553	-0.49251	-3.34841
H	-1.11838	-0.18721	-3.92466
H	0.660291	-0.23293	-3.92755
C	-0.26699	-1.98581	-3.06253
H	-1.14108	-2.25057	-2.45627
H	0.636229	-2.29403	-2.52208
C	-0.33612	-2.73013	-4.39226
H	-1.25899	-2.49823	-4.94139
H	0.525358	-2.50254	-5.03513
S	-0.32664	-4.50743	-4.16484
O	-0.44315	-5.09542	-5.53488
O	0.973654	-4.84761	-3.50691
O	-1.50275	-4.83349	-3.29919
H	-0.12777	1.346087	-2.57804

MOPS-depr

N	-0.21116	0.413816	-2.14275
O	-0.2832	-0.39193	0.630612
C	-1.451	-0.53876	-0.17702
H	-1.51504	-1.58009	-0.53376
H	-2.31431	-0.3417	0.472696
C	0.895005	-0.62723	-0.13961
H	1.750043	-0.49104	0.536162
H	0.894361	-1.67205	-0.49167
C	-1.43027	0.452768	-1.33733
H	-1.53897	1.464088	-0.90994
H	-2.29992	0.277437	-1.98608
C	0.983552	0.358285	-1.30208
H	1.159144	1.359598	-0.87325
H	1.854756	0.112802	-1.92538
C	-0.2301	-0.46991	-3.30204
H	-1.07914	-0.16472	-3.93426
H	0.681722	-0.26381	-3.88459
C	-0.32377	-1.98575	-3.07343
H	-1.2573	-2.23441	-2.55144
H	0.512314	-2.32989	-2.45148
C	-0.28351	-2.70808	-4.41444
H	-1.11666	-2.41006	-5.06648
H	0.660089	-2.52359	-4.94699
S	-0.40684	-4.48586	-4.24994
O	-0.30295	-5.03927	-5.63758
O	0.731738	-4.91992	-3.37927
O	-1.73667	-4.77407	-3.62392

7 References

1. Howarth, A. J.; Peters, A. W.; Vermeulen, N. A.; Wang, T. C.; Hupp, J. T.; Farha, O. K., Best Practices for the Synthesis, Activation, and Characterization of Metal–Organic Frameworks. *Chem. Mater.* **2017,** *29* (1), 26-39.
2. Islamoglu, T.; Idrees, K. B.; Son, F. A.; Chen, Z.; Lee, S.-J.; Li, P.; Farha, O. K., Are you using the right probe molecules for assessing the textural properties of metal–organic frameworks? *J. Mater. Chem. A* **2022,** *10* (1), 157-173.
3. Ingram, Z. J.; Lander, C. W.; Oliver, M. C.; Altınçekiç, N. G.; Huang, L.; Shao, Y.; Noh, H., Hydrogen-Atom Binding Energy of Structurally Well-defined Cerium Oxide Nodes at the Metal−Organic Framework-Liquid Interfaces. *J. Phys. Chem. C* **2024,** *128* (23), 9556–9565.
4. Ho, W. H.; Li, S.-C.; Wang, Y.-C.; Chang, T.-E.; Chiang, Y.-T.; Li, Y.-P.; Kung, C.-W., Proton-Conductive Cerium-Based Metal–Organic Frameworks. *ACS Appl. Mater. Interfaces* **2021,** *13* (46), 55358-55366.
5. Lammert, M.; Wharmby, M. T.; Smolders, S.; Bueken, B.; Lieb, A.; Lomachenko, K. A.; Vos, D. D.; Stock, N., Cerium-based metal organic frameworks with UiO-66 architecture: synthesis, properties and redox catalytic activity. *Chem. Commun.* **2015,** *51* (63), 12578-12581.
6. Shen, C.-H.; Chuang, C.-H.; Gu, Y.-J.; Ho, W. H.; Song, Y.-D.; Chen, Y.-C.; Wang, Y.-C.; Kung, C.-W., Cerium-Based Metal–Organic Framework Nanocrystals Interconnected by Carbon Nanotubes for Boosting Electrochemical Capacitor Performance. *ACS Appl. Mater. Interfaces* **2021,** *13* (14), 16418-16426.
7. Agarwal, R., The Nicholson Method of Determination of the Standard Rate Constant of a Quasireversible Redox Couple Employing Cyclic Voltammetry: Everything One Needs to Know! *ACS Electrochemistry* **2025,** *1* (10), 1885-1894.
8. Verma, S.; Singh, A.; Shukla, A.; Kaswan, J.; Arora, K.; Ramirez-Vick, J.; Singh, P.; Singh, S. P., Anti-IL8/AuNPs-rGO/ITO as an Immunosensing Platform for Noninvasive Electrochemical Detection of Oral Cancer. *ACS Appl. Mater. Interfaces* **2017,** *9* (33), 27462-27474.
9. Randviir, E. P., A cross examination of electron transfer rate constants for carbon screen-printed electrodes using Electrochemical Impedance Spectroscopy and cyclic voltammetry. *Electrochim. Acta* **2018,** *286*, 179-186.
10. Altınçekiç, N. G.; Lander, C. W.; Roslend, A.; Yu, J.; Shao, Y.; Noh, H., Electrochemically Determined and Structurally Justified Thermochemistry of H atom Transfer on Ti-Oxo Nodes of the Colloidal Metal–Organic Framework Ti-MIL-125. *J. Am. Chem. Soc.* **2024,** *146* (49), 33485–33498.
11. Altınçekiç, N. G.; Lander, C. W.; Yu, J.; Roslend, A.; Shao, Y.; Noh, H., Proton, Electron, and Hydrogen-Atom Transfer Thermodynamics of the Metal–Organic Framework, Ti-MIL-125, Are Intrinsically Correlated to the Structural Disorder. *J. Am. Chem. Soc.* **2025,** *147* (38), 34777-34790.
12. Neese, F., Software update: The ORCA program system—Version 5.0. *WIREs Comput Mol Sci.* **2022,** *12* (5), e1606.
13. Grimme, S.; Hansen, A., A Practicable Real-Space Measure and Visualization of Static Electron-Correlation Effects. *Angew. Chem. Int. Ed.* **2015,** *54* (42), 12308-12313.

14. Mardirossian, N.; Head-Gordon, M., ωB97M-V: A combinatorially optimized, range-separated hybrid, meta-GGA density functional with VV10 nonlocal correlation. *J. Chem. Phys.* **2016,** *144* (21), 214110.
15. Weigend, F.; Ahlrichs, R., Balanced basis sets of split valence, triple zeta valence and quadruple zeta valence quality for H to Rn: Design and assessment of accuracy. *Phys. Chem. Chem. Phys.* **2005,** *7* (18), 3297-3305.
16. Marenich, A. V.; Cramer, C. J.; Truhlar, D. G., Universal Solvation Model Based on Solute Electron Density and on a Continuum Model of the Solvent Defined by the Bulk Dielectric Constant and Atomic Surface Tensions. *J. Phys. Chem. B* **2009,** *113* (18), 6378-6396.
17. Dutra, F. R.; Silva, C. d. S.; Custodio, R., On the Accuracy of the Direct Method to Calculate pKa from Electronic Structure Calculations. *J. Phys. Chem. A* **2021,** *125* (1), 65-73.
